# Identification and Assessment of Systematic Reviews for Evidence‐Based Guideline Recommendations on Follow‐Up of Preterm Born Children: A Mapping Review

**DOI:** 10.1111/apa.70507

**Published:** 2026-04-24

**Authors:** Gabriel Kaiser, Marie Kayser, Marie Babel, Judith Wohlleben, Stephanie Weibel, Juliane Spiegler

**Affiliations:** ^1^ Department of Pediatrics University Hospital Würzburg Würzburg Germany; ^2^ Department of Anesthesiology, Intensive Care, Emergency and Pain Medicine University Hospital Würzburg Würzburg Germany

**Keywords:** developmental outcomes, evidence mapping, guideline development, preliminary screening, preterm birth

## Abstract

**Aim:**

This mapping review presents a systematic approach identifying aggregated evidence and assessing methodological quality.

**Methods:**

MEDLINE (via Ovid), Epistemonikos and the Cochrane database were searched for systematic reviews, with or without meta‐analyses, published from 2010 to May 6, 2025, addressing any interventions, prognosis, risks, incidence, prevalence, or diagnosis in preterm or very low birth weight children. To ensure quality, we included only those that searched at least two databases, assessed risk of bias, and defined a clear research question according to the PICO framework. Systematic reviews of interventions were assessed for methodological quality using AMSTAR 2. Those on risks, prognosis, prevalence, diagnostics, and qualitative research were assessed with ROBIS following a preliminary quality screening.

**Results:**

Of 8000 references, 643 full texts were assessed, 239 systematic reviews met inclusion criteria: 42 interventional, 183 prognosis, risks, incidence and prevalence, 11 diagnostic, and 3 qualitative. Among interventional systematic reviews, seven showed high‐ or moderate‐quality. Out of 197 systematic reviews, preliminary quality screening excluded 192 systematic reviews; ROBIS identified two high‐quality prognosis and risk reviews out of five.

**Conclusion:**

Quality of data on post discharge follow‐up in preterm‐born children is poor. Preliminary screening improves efficiency by limiting assessment time spent on low‐quality reviews.

## Summary

- This mapping review assessed systematic reviews on post‐discharge follow‐up of preterm‐born children to inform the corresponding German AWMF evidence‐based guideline.
- From 8000 references, 239 systematic reviews were assessed using AMSTAR 2 for interventional reviews and an adapted preliminary screening for risk, prognosis, prevalence, diagnostic, and qualitative reviews before ROBIS.
- High‐quality evidence was limited, highlighting the value of preliminary quality screening to timely identify reviews suitable for information of evidence‐based guidelines.

**Abbreviations**
AMSTAR: a measurement tool to assess systematic reviews
AWMF: Association of the Scientific Medical Societies in Germany
BPD: bronchopulmonary dysplasia
EEG: electroencephalography
ENT: ear, nose, and throat
IVH: intraventricular haemorrhage
MRI: magnetic resonance imaging
NICU: neonatal intensive care unit
PVL: periventricular leukomalacia
ROBIS: Risk of Bias in Systematic Reviews
ROP: retinopathy of prematurity
RSV: respiratory syncytial virus
VLBW: very low birth weight

## 1 Introduction

Approximately 15 million babies each year are born preterm globally, accounting for about 11% of all births worldwide [1]. Preterm birth, defined as birth before the 37th week of gestation, presents challenges for the affected children and their families but also for healthcare professionals and systems [2].

Compared to full‐term born children, those born preterm have an increased risk of organic illnesses, developmental delays, for example, neurodevelopmental, motor or language, developmental and behavioural disorders such as autism spectrum disorder or attention deficit disorder [3, 4, 5].

These elevated risks lead to the need for structured, long‐term follow‐up care that extends beyond the immediate neonatal period in hospital, aiming to improve the quality of life and participation and mitigating long‐term health effects of premature‐born children and their families [6].

As part of updating and upgrading a German guideline on post‐discharge follow‐up care for preterm‐born children (Registry Number 071–013), we conducted a systematic literature search in accordance with the standards of the Association of Scientific Medical Societies in Germany (AWMF) [7].

As the guideline aimed to comprehensively address all aspects of follow‐up care, detailed PICO questions were not predefined, as it is frequently done in systematic guideline development. Instead, we applied a broad search strategy to identify all available evidence across the entire topic, which resulted in a large amount of systematic reviews.

Given the common limitations in time, personnel, and financial resources, structured approaches were essential to efficiently identify high‐quality reviews suitable for informing guideline development.

This mapping review aims to illustrate an approach to efficiently identify existing aggregated evidence of high or moderate quality and thus to support the development of evidence‐based guidelines for preterm‐born children.

## 2 Methods

### 2.1 Protocol and Registration

The new guideline has been officially registered with the registry number 071‐013 in accordance to the standards of the Association of Scientific Medical Societies in Germany (AWMF) [7]. We did not publish or register a protocol for this mapping review.

### 2.2 Eligibility Criteria

The inclusion criteria encompassed systematic reviews with or without meta‐analyses published in English or German between 2010 and May 6, 2025. Eligible systematic reviews focused on preterm children (gestational age < 37 weeks) or children with very low birth weight (VLBW), addressing the following types of systematic reviews [8]: interventional, risk, prevalence or incidence, prognostic, diagnostic, or qualitative reviews with any post‐discharge outcome assessed post‐hospital discharge.

The systematic reviews had to focus on studies investigating preterm‐born or VLBW children or include a clearly identifiable subgroup of such children. Given that VLBW infants are almost always preterm, reviews including them as full or subgroup populations were also accepted.

We used the PICO framework [8] to structure our research questions, and question formats were adapted according to the specific subtype.

Eligible systematic reviews must report outcomes belonging to any of the following broadly defined outcome categories:

- Organ structure and function.
- Participation in all dimensions of the International Classification of Function (ICF) [9].
  ○ Learning and applying knowledge.
  ○ General tasks and demands.
  ○ Communication.
  ○ Mobility.
  ○ Self‐care.
  ○ Domestic life.
  ○ Interpersonal interactions and relationships.
  ○ Major life areas.
  ○ Community, social and civic life.
- Quality of life measured in any form.
- Transition to home.

Reasons for exclusion were inappropriate study design or publication type (e.g., dissertations), absence of follow‐up beyond the neonatal hospital stay, and lack of relevant long‐term child outcomes. Systematic reviews were also excluded if they reported only in‐hospital outcomes, diagnoses without long‐term risk implications, or did not provide measurable outcomes in the children.

A more detailed list of inclusion and exclusion criteria using the PICO framework can be found in Appendix S1.

For full‐text screening, we added the following criteria considering basic methodological standards of systematic reviews: systematic reviews must have defined a clear research question according to the question framework, conducted a comprehensive search in at least two databases and reported the search strategy and/or keyword search. Furthermore, a rigorous appraisal of each included study must have been conducted using any risk of bias assessment tool.

### 2.3 Information Sources and Literature Search

We performed a comprehensive literature search in MEDLINE (Ovid) and Epistemonikos from January 2010 to the 5th of October 2023. Full search strategies are provided in Appendix S2.

We imported the retrieved citations into Covidence (Covidence systematic review software, Veritas Health Innovation, Melbourne, Australia). Two independent reviewers screened titles and abstracts according to our defined eligibility criteria [8].

A two‐step eligibility full‐text screening with extended eligibility criteria ensuring basic methodological standards of systematic reviews was then conducted by two independent reviewers. Any conflicts were resolved in discussions with experts.

A search update limited to the Cochrane Database of Systematic Reviews was conducted on May 6, 2025, based on the experience that Cochrane reviews adhere to the high methodological standards required for guideline development. Results were imported into EndNote and screened at title and abstract and full‐text levels by two independent reviewers. Conflicts were resolved as above.

### 2.4 Data Extraction

Two independent reviewers performed data extraction using Covidence. Any conflicts were solved by a third person.

Data items extracted included general systematic review information and information about participants or population, intervention or index prognostic factors or condition, outcomes and context or timing. The data extraction sheet is available in Appendix S3.

### 2.5 Methodological Quality Assessment

In a first step, we assessed the methodological quality of interventional systematic reviews using the AMSTAR‐2 tool [10].

**FIGURE 1:**
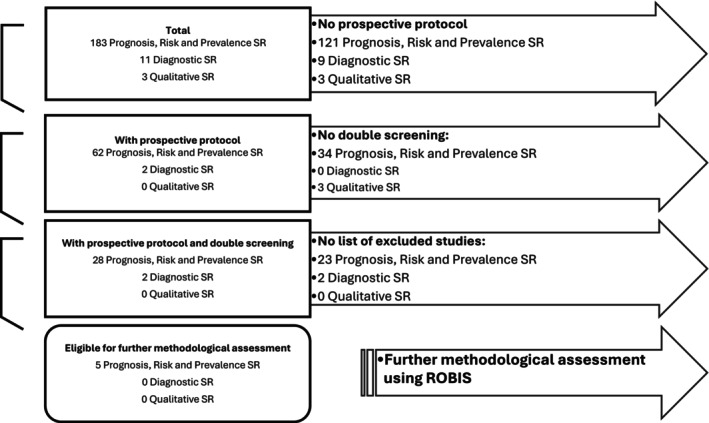
Hierarchical preliminary quality screening.

Reviews for prognosis, risk and prevalence as well as diagnostic and qualitative reviews were pre‐screened using a preliminary quality screening approach developed in‐house and informed by our experience with AMSTAR 2 used for interventional systematic reviews (Figure 1). For the extended quality assessment, we used the ROBIS tool [11], as AMSTAR 2 is designed exclusively for evaluating the quality of interventional systematic reviews. Only systematic reviews that passed the preliminary quality screening were assessed using ROBIS. Each screening was performed by two independent reviewers.

### 2.6 Mapping of Aggregated Evidence

**FIGURE 2:**
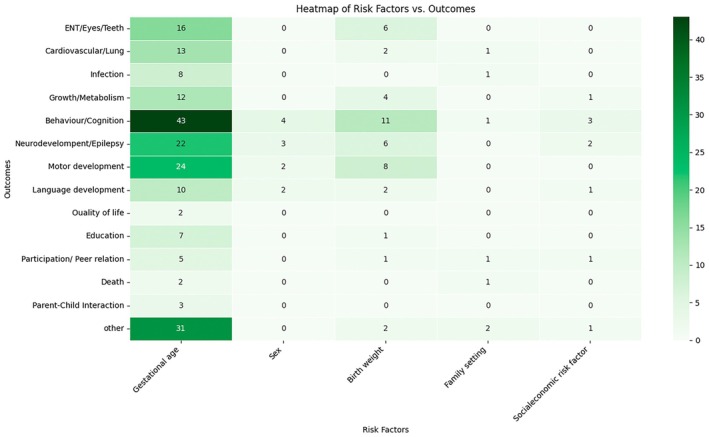
Heat‐map of outcomes vs. children‐based Risk Factors (*n* = numbers of systematic reviews regarding risk, prognosis and prevalence).

**FIGURE 3:**
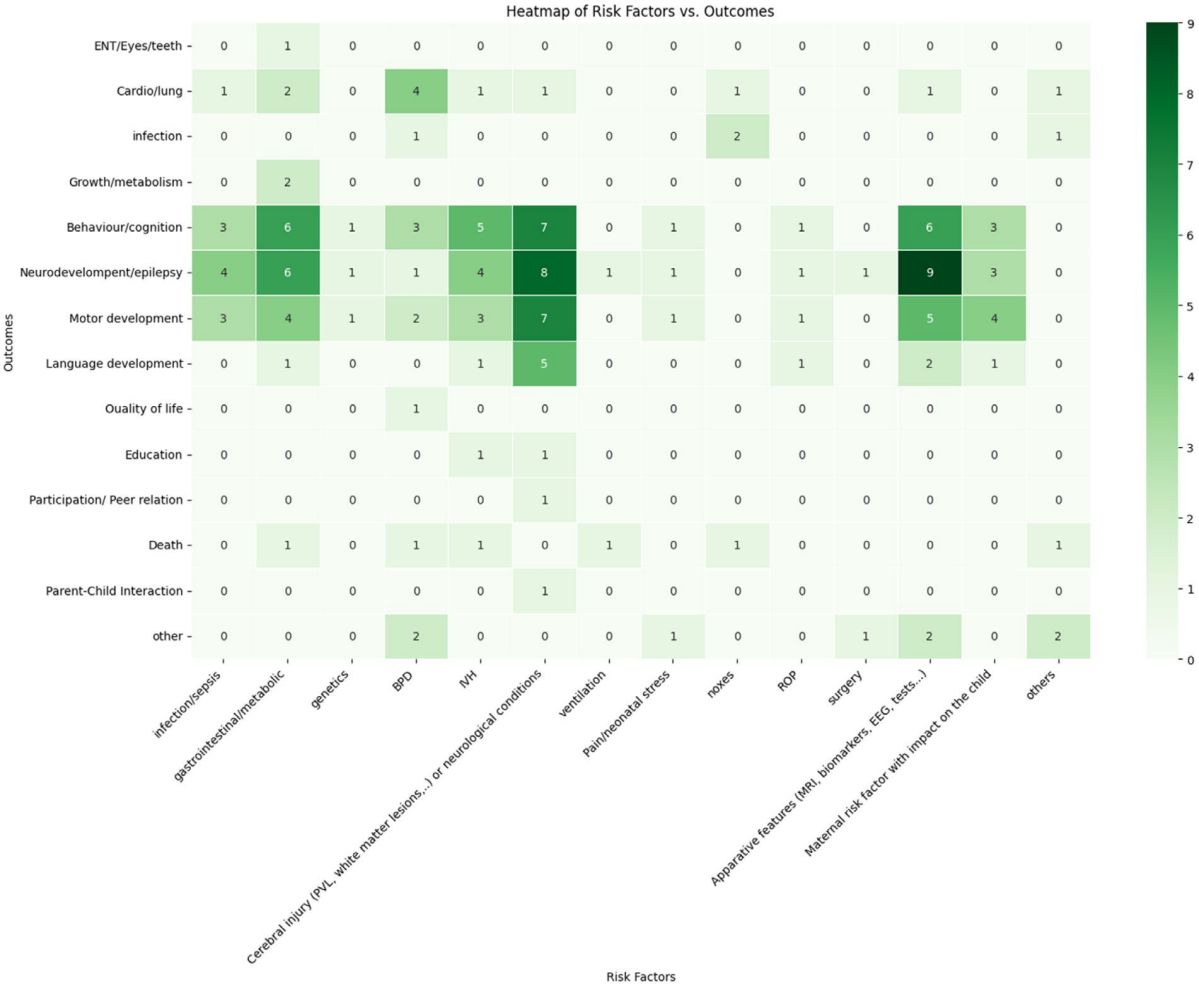
Heat‐map of outcomes vs. medical Risk Factors (*n* = numbers of systematic reviews regarding risk, prognosis and prevalence).

In a table, we mapped systematic interventional reviews and systematic prognosis, risk and prevalence reviews for thematic categories (e.g., interventions, risk factors) as well as outcome‐categories (Tables 1 and 2, Figures 2 and 3).

**TABLE 1:** AMSTAR 2 assessment of SRs on pharmaceutical interventions tabulated according to outcome categories.

Reported outcomes	Lung	Vision and hearing	Heart	Recurrence, complication	Metabolism	Motor development	Neuro development	Cognition	Behaviour	Parent–child interaction
Pharmaceutical intervention										
Medications			Manapurath et al. [12]		Manapurath et al. [12]	Moreno‐Fernandez et al. [13]	Manapurath et al. [12]	Manapurath et al. [12]	Manapurath et al. [12]	
					McCarthy et al. [14]		McCarthy et al. [14]			
					Long et al. [15]		Moreno‐Fernandez et al. [13]		Moreno‐Fernandez et al. [13]	
					Mills and Davies [16]		Mills and Davies [16]			
Vaccination										
Nutrition			Elfzzani et al. [17]		Elfzzani et al. [17]	Lin et al. [18]	Elfzzani et al. [17]	Elfzzani et al. [17]		Elfzzani et al. [17]
					Lin et al. [19]					
					Vissers et al. [20]		Lin et al. [18]	Lin et al. [18]		
					Huang et al. [21]					
					Lin et al. [18]	Lin et al. [22]	Long et al. [15]	Lin et al. [22]		
					Lin et al. [23]					
					Lin et al. [22]					
			Lin et al. [22]		Ahmed et al. [24]					
Food‐supplements		Young et al. [25]	Young et al. [25]		Young et al. [25]	Young et al. [25]	Young et al. [25]	Young et al. [25]		
					Kumar et al. [26]		Kumar et al. [26]	Young et al. [27]		
			Young et al. [27]		Young et al. [27]	Kumar et al. [28]	Young et al. [27]	Kumar et al. [28]		
					Kumar et al. [28]		Kumar et al. [28]			
Surgery/anaesthesia	Choo et al. [29]			Masoudian et al. [30]			Jones et al. [31]			
				Olesen et al. [32]						
				Pogorelic et al. [33]						

*Note:* AMSTAR 2 grading, ranging from high (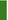) to moderate (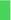), low (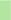), and critically low (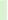).

**TABLE 2:** SRs on non‐pharmaceutical interventions tabulated according to outcome categories.

Reported outcomes	Lung	Heart	Metabolism	Motor development	Neuro development	Cognition	Behaviour	Parent–child interaction	Education	Language	Peer relation/social
Non‐pharmaceutical intervention											
Motor‐training				Hughes et al. [34]							
				Javier et al. [35]							
Physical activity		Spiegler et al. [36]									
Leisure, social activity			Santos et al. [37]	Santos et al. [37]	Santos et al. [37]						
Behavorial training	Bieleninik et al. [38]			Inamdar et al. [39]	Inamdar et al. [39]		Dell'Aversana et al. [40]				
Early developmental interventions				Orton et al. [41]	Ferreira et al. [42]	Orton et al. [41]	Patronick et al. [43]	Barlow et al. [44]		Ferreira et al. [42]	Barlow et al. [44]
								Kermani et al. [45]			
								Patronick et al. [43]			
								Zhang et al. [46]		Zhang et al. [46]	
				Barlow et al. [44]		Barlow et al. [44]	Zhang et al. [46]	Evans et al. [47]			
				Ferreira et al. [42]		Ferreira et al. [42]	Herd et al. [48]	Benzies et al. [49]			
Follow‐up care after discharge			Mohandas et al. [50]	Yang et al. [51]	Yang et al. [51]	Yang et al. [51]	Yang et al. [51]	Mohandas et al. [50]		Yang et al. [51]	
			Collins et al. [52]								
			Yang et al. [51]	Goyal et al. [53]	Goyal et al. [53]	Goyal et al. [53]	Goyal et al. [53]	Goyal et al. [53]		Goyal et al. [53]	
			Goyal et al. [53]								
Parent–child interaction											

*Note:* AMSTAR 2 grading, ranging from high (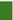) to moderate (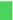), low (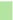), and critically low (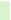).

Interventional systematic reviews were categorised as pharmaceutical (medication, vaccination, nutrition and food supplements, surgery and anaesthesia) or non‐pharmaceutical (motor training, physical activity, leisure and social activities, behavioural training, early developmental interventions, follow‐up care, parent–child interaction). Outcome categories included lung, heart, vision and hearing, metabolism, motor development, neurodevelopment, recurrence or complication, cognition, behaviour, parent–child interaction, education, language, and peer relation or social. Systematic reviews were classified according to the outcomes assessed.

The distribution of reviews by AMSTAR 2 rating, thematic category, and outcome is presented in Tables. AMSTAR 2 ratings (high, moderate, low and critically low) are visually distinguished by progressively darker shades of green.

For prognosis and risk systematic reviews, we mapped the number of systematic reviews (*n*) assigned to each category or outcome, irrespective of systematic review quality, to identify evidence gaps using a heatmap. Outcome categories included Ear‐nose‐throat (ENT), eyes and teeth, cardiovascular or lung, infection, growth or metabolism, behaviour or cognition, neurodevelopment or epilepsy, motor development, language development, quality of life, education, participation or peer relation, death, parent–child interaction and other. Child‐related risk factors included gestational age, sex, birth weight, family setting, and socioeconomic risk factors. Medical risk factors comprised infection or sepsis, gastrointestinal or metabolic, genetics, bronchopulmonary dysplasia (BPD), intraventricular haemorrhage (IVH), cerebral injury or other neurological condition, ventilation, pain or neonatal stress, noxes, retinopathy of prematurity (ROP), surgery, apparative features, maternal risk factors with impact on the child, and others.

The heatmaps (Figures 2 and 3) provide an overview of the number of systematic reviews addressing the respective risk factors and outcomes, irrespective of quality assessment.

### 2.7 Usage of AI

An AI language model (ChatGPT, OpenAI, San Francisco, CA, USA) was employed to assist with table formatting. Any content produced with the assistance of artificial intelligence was critically reviewed, and validated by the authors to ensure accuracy and integrity.

## 3 Results

We identified 8000 references from the literature search. After removing duplicates and screening titles and abstracts, we conducted a full‐text assessment on 643 publications. We excluded 381 studies during full text screening. Most studies were excluded based on our eligibility criteria ensuring basic methodological standards (two‐step eligibility screening) of systematic reviews (*n* = 133), followed by unclear or wrong time point of intervention. A list of excluded studies can be found in Appendix S4.

**FIGURE 4:**
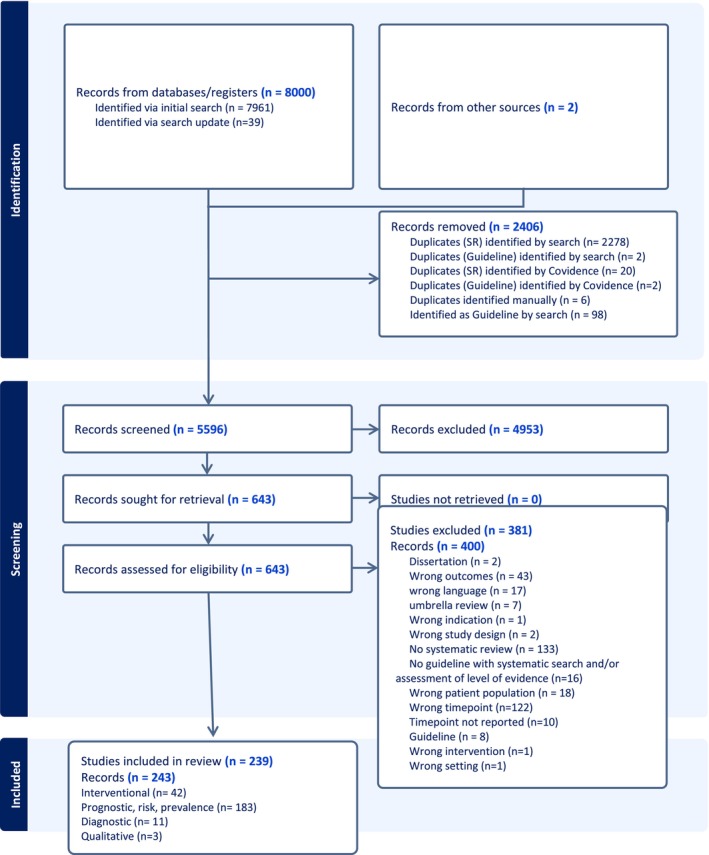
Prisma.

Finally, a total of 239 systematic reviews met the eligibility criteria and were included in this study (Figure 4, PRISMA).

### 3.1 Methodological Quality

The 239 identified systematic reviews fulfilling the basic methodological standards of systematic reviews were divided into 42 interventional systematic reviews, 183 systematic reviews covering prognosis, risk and prevalence, 11 diagnostic reviews, and 3 systematic reviews of qualitative studies.

Characteristics of included systematic reviews can be found in Appendix S5.

### 3.2 AMSTAR 2 of Interventional Systematic Reviews

Using AMSTAR 2, 4 out of 42 interventional systematic reviews were rated as high, 3 as moderate, 11 as low and 24 as critically low quality.

Among the 24 critically low systematic interventional reviews, 19/24 lacked a pre‐registered protocol (Item 2), 7/24 did not perform double screening during data extraction (Item 6), and 8/24 did not conduct double screening during study inclusion (Item 5). About 22/24 reviews did not provide a list of excluded studies (Item 7). Among the 11 low‐quality reviews, 2/11 lacked a pre‐registered protocol (Item 2), 2/11 did not conduct double screening for data extraction (Item 6), and 8/11 did not provide a list of excluded studies (Item 7). Table 3 shows an overview of AMSTAR‐Items and grading.

**TABLE 3:** Amstar 2 rating of interventional systematic reviews.

Review	Item I	Item 2	Item 3	Item 4	Item 5	Item 6	Item 7	Item 8	Item 9		Item 10	Item 11		Item 12	Item 13	Item 14	Item 15	Item 16	Overall Confidence
Manapurath et al. [12]	Yes	Yes	No	Partial yes	Yes	Yes	Yes	Yes	Yes	Yes	No	No	Includes only RCTs	Yes	Yes	Yes	Yes	Yes	Low
McCarthy et al. [14]	Yes	Yes	No	Yes	Yes	Yes	No	No	Yes	No	No	No meta‐analysis conducted	No meta‐analysis conducted	No meta‐analysis conducted	No	Yes	No meta‐analysis conducted	Yes	Critically low
Elfzzani et al. [17]	Yes	Yes	No	Yes	Yes	Yes	Yes	No studies found	No studies found	No studies found	Yes	No meta‐analysis conducted	No meta‐analysis conducted	No meta‐analysis conducted	Yes	Yes	No meta‐analysis conducted	Yes	High
Vissers et al. [20]	Yes	Yes	No	Yes	Yes	No	No	Yes	Yes	Yes	No	No meta‐analysis conducted	No meta‐analysis conducted	No meta‐analysis conducted	Yes	Yes	No meta‐analysis conducted	Yes	Low
Moreno‐Fernandez et al. [13]	Yes	No	No	Partial yes	Yes	Yes	No	Yes	Partial yes	No	No	No meta‐analysis conducted	No meta‐analysis conducted	No meta‐analysis conducted	Yes	No	No meta‐analysis conducted	Yes	Critically low
Young et al. [25]	Yes	Yes	No	Yes	Yes	Yes	Yes	Yes	Yes	Yes	Yes	Yes	Includes only RCTs	Yes	Yes	Yes	Yes	Yes	High
Kumar et al. [26]	Yes	Yes	No	Partial yes	Yes	Yes	No	Yes	Yes	Includes only RCTs	Yes	Yes	Includes only RCTs	Yes	Yes	Yes	Yes	Yes	Low
Mills and Davies [16]	Yes	Partial yes	No	Yes	Yes	Yes	Yes	Partial yes	Yes	Yes	No	No	No	No	Yes	Yes	No	Yes	Critically low
Long et al. [15]	Yes	No	No	Partial yes	Yes	Yes	No	No	Partial yes	No	No	No meta‐analysis conducted	No meta‐analysis conducted	No meta‐analysis conducted	Yes	Yes	No meta‐analysis conducted	Yes	Critically low
Choo et al. [29]	Yes	Yes	No	Partial yes	Yes	Yes	No	Yes	Includes only NRSI	Yes	No	Includes only NRSIs	Yes	Yes	Yes	Yes	Yes	Yes	Low
Jones et al. [31]	Yes	Yes	No	Yes	Yes	Yes	Yes	Partial yes	Yes	Includes only RCTs	No	Yes	Includes only RCTs	Yes	Yes	Yes	Yes	Yes	Moderate
Olesen et al. [32]	Yes	Partial yes	No	Partial yes	Yes	No	No	Partial yes	Yes	Partial yes	No	No meta‐analysis conducted	No meta‐analysis conducted	No meta‐analysis conducted	Yes	Yes	No meta‐analysis conducted	Yes	Low
Pogorelic et al. [33]	Yes	No	No	Partial yes	Yes	Yes	No	No	Includes only NRSI	No	No	Includes only NRSIs	Yes	No	Yes	Yes	Yes	Yes	Critically low
Inamdar et al. [39]	Yes	Partial yes	No	Partial yes	Yes	Yes	No	Yes	Yes	Yes	No	No meta‐analysis conducted	No meta‐analysis conducted	No meta‐analysis conducted	Yes	Yes	No meta‐analysis conducted	Yes	Low
Dell'Aversana et al. [40]	Yes	No	No	Partial yes	No	No	No	Partial yes	Partial yes	Includes only RCTs	No	No meta‐analysis conducted	No meta‐analysis conducted	No meta‐analysis conducted	Yes	No	No meta‐analysis conducted	Yes	Critically low
Orton et al. [41]	Yes	Yes	No	Yes	Yes	Yes	Yes	Yes	Yes	Yes	Yes	Yes	Yes	Yes	Yes	Yes	Yes	Yes	High
Santos et al. [37]	Yes	Yes	No	Partial yes	No	Yes	No	Partial yes	Includes only NRSI	Yes	No	No meta‐analysis conducted	No meta‐analysis conducted	No meta‐analysis conducted	No	No	No meta‐analysis conducted	Yes	Critically low
Patronick et al. [43]	Yes	No	No	Partial yes	No	No	No	Yes	Yes	No	No	No meta‐analysis conducted	No meta‐analysis conducted	No meta‐analysis conducted	Yes	Yes	No meta‐analysis conducted	Yes	Critically low
Kermani et al. [45]	Yes	No	No	Partial yes	Yes	Yes	No	Yes	No	No	No	No meta‐analysis conducted	No meta‐analysis conducted	No meta‐analysis conducted	No	Yes	No meta‐analysis conducted	Yes	Critically low
Mohandas et al. [50]	Yes	Yes	No	Partial yes	Yes	Yes	Yes	Yes	Yes	Yes	No	No meta‐analysis conducted	No meta‐analysis conducted	No meta‐analysis conducted	Yes	Yes	No meta‐analysis conducted	Yes	Moderate
Benzies et al. [49]	Yes	No	No	Partial yes	Yes	No	No	Partial yes	Yes	Includes only RCTs	No	Yes	Includes only RCTs	No	No	No	Yes	Yes	Critically low
Ferreira et al. [42]	Yes	Partial yes	No	Partial yes	Yes	Yes	No	Yes	Yes	Includes only RCTs	No	Yes	Includes only RCTs	No	Yes	Yes	No	Yes	Critically low
Hughes et al. [34]	Yes	No	No	Partial yes	No	No	No	Partial yes	Yes	No	No	No	No	No	No	No	No	Yes	Critically low
Javier et al. [35]	Yes	No	No	Partial yes	No	No	No	Partial yes	Partial yes	Partial yes	No	No meta‐analysis conducted	No meta‐analysis conducted	No meta‐analysis conducted	Yes	Yes	No meta‐analysis conducted	No	Critically low
Masoudian et al. [30]	Yes	Yes	No	Partial yes	Yes	Yes	No	Partial yes	Includes only NRSI	Partial yes	No	Includes only NRSIs	Yes	No	Yes	No	Yes	Yes	Low
Colllins et al. [52]	Yes	Yes	No	Partial yes	No	Yes	Yes	Yes	Includes only NRSI	Yes	No	Includes only NRSIs	Yes	Yes	Yes	Yes	No meta‐analysis conducted	Yes	Moderate
Goyal et al. [53]	Yes	No	No	Partial yes	Yes	Yes	No	Yes	No	No	No	Yes	No	No	No	Yes	Yes	Yes	Critically low
Lin et al. [18]	Yes	No	No	Partial yes	Yes	0	No	No	Yes	Yes	No	0	0	Yes	No	Yes	No	Yes	Critically low
Lin et al. [23]	Yes	No	No	Partial yes	Yes	0	Yes	No	Yes	Yes	No	0	0	Yes	No	Yes	No	Yes	Critically low
Lin et al. [22]	Yes	No	No	Partial yes	Yes	Yes	No	Yes	Yes	Yes	Yes	Yes	Includes only RCTs	Yes	Yes	Yes	Yes	Yes	Critically low
Lin et al. [19]	Yes	Partial yes	No	Partial yes	Yes	Yes	No	Yes	Yes	Yes	Yes	Yes	Yes	Yes	Yes	Yes	Yes	Yes	Low
Kumar et al. [28]	Yes	No	No	Partial yes	Yes	Yes	No	Yes	Yes	Includes only RCTs	Yes	Yes	Includes only RCTs	Yes	Yes	Yes	Yes	Yes	Critically low
Spiegler et al. [36]	Yes	Partial yes	No	Partial yes	Yes	Yes	No	Yes	No	Partial yes	No	No meta‐analysis conducted	No meta‐analysis conducted	No meta‐analysis conducted	No	No	No meta‐analysis conducted	Yes	Critically low
Yang et al. [51]	Yes	Yes	No	Partial yes	Yes	Yes	No	Yes	Yes	Includes only RCTs	No	No meta‐analysis conducted	No meta‐analysis conducted	No meta‐analysis conducted	Yes	Yes	No meta‐analysis conducted	Yes	Low
Barlow et al. [44]	Yes	Yes	No	Yes	Yes	Yes	Yes	Yes	Yes	Includes only RCTs	Yes	Yes	Includes only RCTs	Yes	Yes	Yes	Yes	Yes	High
Herd et al. [48]	Yes	No	No	Partial yes	Yes	No	No	Partial yes	Partial yes	Includes only RCTs	No	Yes	Includes only RCTs	Yes	No	Yes	No	Yes	Critically low
Evans et al. [47]	Yes	No	No	Partial yes	No	Yes	No	Partial yes	Partial yes	No	No	Includes only NRSIs	Yes	Yes	No	Yes	No	No	Critically low
Young et al. [27]	Yes	Yes	No	Yes	Yes	Yes	Yes	Partial yes	Yes	Includes only RCTs	Yes	No	Includes only RCTs	Yes	Yes	Yes	Yes	Yes	Low
Zhang et al. [46]	Yes	No	No	Partial yes	Yes	Yes	No	Yes	Partial yes	Includes only RCTs	No	No meta‐analysis conducted	Includes only RCTs	No meta‐analysis conducted	No	No	No meta‐analysis conducted	Yes	Critically low
Huang et al. [21]	Yes	Partial yes	No	Partial yes	Yes	Yes	Yes	Yes	Includes only NRSI	Partial yes	No	Includes only NRSIs	Yes	No	No	Yes	Yes	Yes	Low
Ahmed et al. [24]	Yes	No	Yes	Partial yes	No	No	No	Yes	Yes	Includes only RCTs	No	No meta‐analysis conducted	No meta‐analysis conducted	No meta‐analysis conducted	No	No	No meta‐analysis conducted	No	Critically low
Bieleninik et al. [38]	Yes	No	Yes	Partial yes	No	Yes	No	Partial yes	Yes	Includes only RCTs	No	Yes	Includes only RCTs	No	No	Yes	No	Yes	Critically low

*Note:* Amstar 2 rating of interventional systematic reviews (extra file).

The absence of a pre‐registered protocol, double‐screening and extraction, and an excluded studies list contributed most to the downgrading of the overall quality rating.

### 3.3 Preliminary Quality Screening of Prognosis, Risk, Prevalence, Diagnostic and Qualitative Systematic Reviews

To enhance efficiency of quality assessment for all other types of systematic reviews (prognosis, risk and prevalence systematic reviews as well as diagnostic reviews and reviews of qualitative studies, *n* = 197), a hierarchical preliminary quality screening was developed based on the items leading most often to downrating of the overall confidence identified during AMSTAR 2 assessment of interventional systematic reviews (Figure 1, preliminary quality screening).

If double screening during data extraction or double screening during study inclusion was not performed, this was an exclusion criterion.

### 3.4 Prognosis, Risk and Prevalence Systematic Reviews

We identified 183 systematic reviews on prognosis, risk, and prevalence.

The hierarchical preliminary quality screening excluded 121/183 reviews on prognosis, risk and prevalence due to the absence of a protocol, 34/62 reviews because screening was not conducted in duplicate, and 23/28 reviews because they did not include a list of excluded studies. After preliminary quality screening, five systematic reviews on prognosis, risk and prevalence were retained for ROBIS assessment [54, 55, 56, 57, 58]. Of these, two were rated as high: Fenton et al. [55], which evaluated the risk of development of blood pressure in preterm small‐for‐gestational‐age versus preterm non‐SGA infants, and Guo et al. [58], which examined the prevalence of autism spectrum disorder. The other three were rated as low.

### 3.5 Diagnostic Reviews and Systematic Reviews of Qualitative Studies

None of the 11 diagnostic reviews and systematic reviews of the three qualitative reviews met the preliminary quality screening criteria, as shown in Figure 1.

### 3.6 Mapping of Interventional Systematic Reviews

The seven interventional systematic reviews rated as moderate or high quality addressed nutrition (*n* = 4, [17, 25, 50, 52]), early intervention (*n* = 2, [41, 44]), and surgery (*n* = 1, [31]).

Of 42 interventional systematic reviews in total, 23 reviews evaluated pharmaceutical/nutritional interventions whereas 19 examined non‐pharmaceutical interventions.

Within the pharmaceutical interventional reviews, we predominantly identified systematic reviews that focused on metabolic and neurodevelopmental outcomes. These reviews addressed interventions related to nutrition, supplementation, and medication (*n* = 17). A particular focus was placed on infant formula feeding, the introduction of complementary foods, and iron supplementation.

Among systematic interventional reviews of non‐pharmaceutical interventions, the main focus was on early developmental interventions and follow‐up care (*n* = 13). No reviews addressing parent–child interaction were identified. Furthermore, no high‐quality reviews were found examining interventions related to leisure and social activities or physical activity.

Outcomes addressed were mostly developmental outcomes, including behaviour and cognition, neurodevelopment and epilepsy, motor skills, and language development. Very few reviews have investigated participation‐related outcomes, including peer relationships, quality of life, educational attainment, or parent–child interactions.

### 3.7 Mapping of Prognosis, Risk and Prevalence Systematic Reviews

Only a very small number of low‐quality systematic reviews on risk, prognosis, and prevalence are available with respect to outcomes such as quality of life, peer relationships, and education. There is only a small number of systematic reviews addressing outcomes related to the cardiovascular, pulmonary, ear‐nose‐throat, and ophthalmologic domains, as well as limited evidence regarding ventilation and surgical interventions.

The investigated risk factors primarily include gestational age, birth weight, neonatal health conditions such as cerebral injuries, intraventricular haemorrhage, bronchopulmonary disease and factors relating to diagnostic devices. There is only a little number of systematic reviews targeting psychosocial influences, such as socioeconomic status or family circumstances.

### 3.8 Mapping of Diagnostic Systematic Reviews

No reviews of high methodological quality were identified. Among the reviews of low methodological quality, *n* = 5 focused on clinical assessment scales (such as the Bayley Scales of Infant and Toddler Development or the General Movements Assessment), while *n* = 4 examined diagnostic tools as, for example, magnetic resonance imaging and electroencephalography.

## 4 Discussion

When addressing a broad research question, as in our guideline project, a large number of systematic reviews can be expected. We deliberately refrained from formulating precise PICO questions in advance and instead kept the research questions intentionally broad. This approach was chosen to ensure that our search captured all literature potentially relevant to a follow‐up care guideline, given that the topic of follow‐up itself is inherently broad and complex.

To accommodate this volume of evidence within the limits of available financial and human resources, we adapted the eligibility criteria, the screening strategy and the quality assessment process of systematic reviews. While this introduces certain limitations, because reviews were not considered further due to their rapid downgrading resulting from methodological limitations, it enables the efficient identification of high‐quality systematic reviews suitable for formulating evidence‐based recommendations.

To manage the number of included systematic reviews, we implemented a structured two‐step screening strategy with specific eligibility criteria regarding the study type of systematic reviews during full text screening.

After screening at full‐text level with our specific eligibility criteria, we conducted a preliminary quality screening afterwards for systematic risk, prognosis, prevalence, diagnostic, and qualitative reviews to determine eligibility for detailed quality assessment. Both AMSTAR 2 and ROBIS apply very strict criteria when assessing methodological quality. Even single missing elements, such as a list of excluded studies or incomplete reporting of search strategies, can lead to a substantial downgrading of overall confidence in the results of the review. Previous research has shown that, despite authors' efforts to adhere to AMSTAR 2 guidance, many reviews still fail to meet all requirements [59].

We found that even when critical items such as screening of at least two databases and the use of a risk of bias assessment tool were fulfilled in advance of the AMSTAR 2 appraisal, the majority of reviews were still rated as low or critically low after full evaluation.

Criticisms about the strong emphasis on reporting quality which may result in downgrading despite otherwise sound methodological conduct have been reported by other authors [60, 61, 62, 63, 64, 65].

Burda et al. criticise that AMSTAR places a substantial focus on reporting quality (e.g., items 5 and 6) and on the assessment of risk of bias (e.g., items 8 and 9) [63]. The authors also emphasise that AMSTAR was originally conceived on the premise that all items contribute comparably to overall methodological quality. AMSTAR 2 partially addresses this by identifying critical domains that can downgrade the overall confidence rating. As Burda et al. describes, other investigators have approached this differently, classifying reviews as high quality when specific key items were fulfilled [63].

ROBIS requires user training [60, 61] and has frequently been applied and interpreted inconsistently. Gates et al. therefore recommend providing additional guidance to ensure more consistent assessments [62]. AMSTAR fits within these criticisms due to its lack of clarity on certain items, which may lead to inconsistent interpretations and applications as reported by various authors [63, 64, 65].

As a suggestion for improvement, simple measures could be implemented and strictly followed, such as providing lists of excluded studies to enhance methodological quality and prevent important findings from being lost due to basic methodological errors.

We identified substantial gaps in research on the follow‐up care of preterm‐born infants, children, and adolescents at the level of aggregated evidence. Only a few systematic interventional reviews or systematic reviews on risk, prognosis, and prevalence met methodological standards. Regarding the search date, it is possible that additional systematic reviews which could fill research gaps have been published in databases other than Cochrane since 5 October 2023 and were therefore not captured in our search update.

Evidence on diagnostic tools during follow‐up was generally of poor quality and insufficient for guideline recommendations. Research on outcomes for children beyond preschool age is scarce, leaving questions relevant to school enrolment, social interactions, or leisure activities unaddressed. Pharmaceutical interventions primarily focus on nutrition and medication, while non‐pharmaceutical interventions mostly target early developmental support and post‐discharge care.

Because very few reviews have investigated participation‐related outcomes, including peer relationships, quality of life, educational attainment, or parent–child interactions, a more robust evidence base is particularly needed to inform participation‐focused follow‐up care.

Overall, only a very small number of reviews were suitable for inclusion in the guideline. Among the interventional systematic reviews, only 7/42 met the criteria for adequate methodological quality. Of these, two (Young et al. [25] on nutrition and Jones et al. [31] on anaesthesia in inguinal hernia surgery) were usable for recommendations in the organ‐specific section. Both reviews addressing early intervention were included in the non–organ‐specific section [41, 44]. During the preliminary quality screening, 178 of 183 reviews focusing on risk, prognosis, or prevalence were excluded. Of the remaining five, two high‐quality reviews were considered thematically relevant and suitable for inclusion. No diagnostic reviews or systematic reviews of qualitative studies were deemed suitable for recommendations.

Following the identification of evidence gaps either due to the absence of systematic reviews or the presence of only low quality systematic reviews we prioritised specific PICOs for the guideline. These PICOs were then addressed through targeted searches of primary literature. The interventions we investigated included: physiotherapy in infancy and childhood, occupational therapy in childhood, early intervention programmes, early institutional support and post‐discharge medical aftercare.

We restricted our search to publications in English and German to support the development of a guideline for German‐speaking countries. This may have reduced comprehensiveness, as 16 reviews published in Spanish, Portuguese, French, Russian, or Chinese were excluded.

Four of the nine eligible systematic reviews addressing interventions, risk, prognosis, and prevalence were less than 5 years old, highlighting recency as an additional evidence gap.

For the search update, we relied exclusively on the Cochrane Database of Systematic Reviews. This decision was based on our prior experience that Cochrane consistently provides reviews of high methodological quality and adequate coverage of the most relevant literature. Although this approach may have limited comprehensiveness, it represented a pragmatic choice aligned with the scope and objectives of our review.

We recommend that further systematic reviews be conducted on the follow‐up care of preterm‐born infants, children, and adolescents. In particular, outcomes related to participation in daily life are important, as they provide essential evidence to inform care and treatment decisions. Special attention should be paid to robust methodological approaches to ensure that findings are not lost due to formal limitations.

## 5 Conclusion

This overview mapped systematic reviews on the follow‐up care of preterm‐born children and adolescents, revealing substantial methodological limitations. Few systematic reviews met quality and reporting standards, highlighted by AMSTAR 2 and ROBIS assessments, and often resulted in downgrading.

The use of a preliminary quality screening, combined with the application of additional criteria at the full‐text review stage, allows for a more efficient use of human and time resources. In this way, a number of reviews with insufficient methodological quality can be excluded before conducting a comprehensive quality assessment. Participation‐related and psycho‐social outcomes were rarely addressed. These findings emphasise the need for higher‐quality, transparently reported research to guide follow‐up care.

## Author Contributions

G.K. and M.K. share first authorship and contributed to data analysis, interpretation and manuscript drafting. M.B. and J.W. did data analysis, methodology support and conceptualisation. S.W. and J.S. provided conceptualisation, methodology support, review, editing and supervision. All authors approved the final manuscript.

## Funding

Funding was provided by the GBA Innovationsfonds (01VSF23009) as part of the development of an evidence‐based guideline for the follow‐up of children born preterm (FrühTEV). The funding source did not participate in the design, conduct or analysis of the review, nor in the writing or submission of this manuscript.

## Conflicts of Interest

The authors declare no conflicts of interest.

## Supporting information


**Appendix S1:** Inclusion and exclusion criteria.

**Appendix S2:** Search strategy.

**Appendix S3:** Data extraction sheet.

**Appendix S4:** Characteristics of excluded studies.

**Appendix S5:** Characteristics of included studies.

## Data Availability

The data that supports the findings of this study are available in the Supporting Information of this article.
