# Supplementary material for: Identification and Assessment of Systematic Reviews for Evidence‐Based Guideline Recommendations on Follow‐Up of Preterm Born Children: A Mapping Review
Source: Acta Paediatr. 2026 Apr 24;115(7):1384–99. doi: 10.1111/apa.70507 (PMC13250969; doi:10.1111/apa.70507)
Supplement: Supplementary file 1 — Appendix S1: Inclusion and exclusion criteria. [file APA-115-1384-s004.docx]

Appendix S1: Inclusion and Exclusion criteria

**Inclusion criteria**

*Systematic reviews of interventions*

| Patient/Population (P) | preterm or VLBW children |
| --- | --- |
| Intervention (I) | any type of pharmacological and non-pharmacological treatment or intervention in the post-discharge setting (or continuing after NICU up to age 18 years |
| Comparison (C) | no intervention or a placebo or any other active intervention |
| Outcome (O) | relevant organ-specific problems in childhood and adolescence |

For interventional systematic reviews, we identified the time-point of intervention (“neonatal intensive care unit (NICU)”, “not-reported”, “post-discharge” and “mixed”, which means both “post-discharge” and “NICU”) to ensure, that only interventions starting or continuing after discharge of the patient were included.

*Systematic reviews of prognosis*

| **Population (P)** | preterm or VLBW children |
| --- | --- |
| **Index prognostic factor (model) (I)** | any prognostic factor e.g. sex, postnatal sepsis, diagnostic tests, prognostic model,… |
| **Comparator prognostic factor (model) (C)** | not applicable (preterm infant population without the prognostic factor), an alternative prognostic factor, or a different prognostic model. |
| **Outcome (O)** | relevant organ-specific problems |
| **Timing and Setting (TS)** | timing of prognostic assessment (e.g., during the NICU stay or at later stage), time horizon of the predicted outcome; context and function of prognostic factors |

*Systematic reviews of risk*

| **Population (P)** | children or adolescence |
| --- | --- |
| **Exposure (E)** | preterm birth or any other risk factor |
| **Outcome (O)** | relevant organ-specific problems |

Maternal risk factors with measurable outcomes in the child were considered as well. Comparators included children born at term for systematic reviews of preterm birth as risk factor or preterm born children without risk factors for systematic reviews of other risks factors.

*Diagnostic systematic reviews*

| **Population (P)** | preterm or VLBW children |
| --- | --- |
| **Index test(s) (I)** | all diagnostic instruments/tests with assessments conducted post-discharge up to 18 years |
| **Reference standard (R)** | gold standard test |
| **Target condition (T)** | relevant organ-specific problems |

*Systematic reviews about prevalence and/or incidence*

| **Condition (Co)** | every specific health condition |
| --- | --- |
| **Context (Co)** | environmental factors, e.g. country, region, setting |
| **Population (Pop)** | preterm or VLBW children within a defined age range |

*Systematic reviews of qualitative studies*

| **Population (P)** | preterm or VLBW children |
| --- | --- |
| **Phenomena of Interest (I)** | experiences with prognostic factors, diagnostic evaluations, developmental assessments (e.g., Bayley at 2 years), or prognostic models |
| **Context (Co)** | post-discharge setting (or continuing after NICU up to age 18 years) |

**Exclusion criteria**

Systematic reviews without reported outcomes belonging to one of the outcome categories (mentioned in the main article) were excluded. We excluded scoping reviews, narrative reviews, dissertations, conference abstracts, study protocols, umbrella reviews and guidelines.

Studies with a topic related in any case to Respiratory Syncytial Virus (RSV) were excluded due to a parallel development of a AWMF S2k-Guideline on RSV for German-speaking countries

Publications focusing solely on children born with low birth weight without specifying gestational age were also excluded.  Maternal or prenatal treatments and risk factors were excluded if they lacked post-discharge outcomes or follow-up beyond the newborn’s hospital stay.

Additionally, outcomes measured during the hospital stay, diagnoses without long-term risk potential and outcomes without measurable outcomes in the children were excluded.
