# Supplementary material for: Identification and Assessment of Systematic Reviews for Evidence‐Based Guideline Recommendations on Follow‐Up of Preterm Born Children: A Mapping Review
Source: Acta Paediatr. 2026 Apr 24;115(7):1384–99. doi: 10.1111/apa.70507 (PMC13250969; doi:10.1111/apa.70507)
Supplement: Supplementary file 2 — Appendix S2: Search strategy. [file APA-115-1384-s002.docx]

Appendix S2: Search strategy

| FruehTEV: Electronic database searches for PICO 1-3 |
| --- |
| **Search for Evidence Syntheses**  **Search for Systematic Reviews**  Search date: 05.10.2023  **Database: Ovid MEDLINE(R) ALL**  1. exp Infant, Premature/  2. exp Infant, low birth weight/  3. Intensive Care, Neonatal/  4. ((premature or pre mature or preterm or pre term) adj3 (infant* or neonat* or neo nat* or baby or babies or newborn? or new born? or newly born? or child or children or adolescen*)).tw.  5. ((neonatal or neo natal) adj3 (prematurity or pre maturity)).tw.  6. ((neonatal or neo natal) adj3 intensive care).tw.  7. (low birth weight or low birthweight).tw.  8. (preemie or premie or preemies or premies).tw.  9. or/1-8  10. *[Filter: by Wong et al. 2006]* systematic reviews.jn. or search*.tw. or meta analysis.pt. or medline.tw. or systematic review.tw.  11. 9 and 10  12. limit 11 to yr="2010 -Current"  = 5267  *Filter: Wong SSL, Wilczynski NL, Haynes RB. (2006). "Comparison of top-performing search strategies for detecting clinically sound treatment studies and systematic reviews in MEDLINE and EMBASE." J Med Libr Assoc 94(4): 451-455.*  **Database: Epistemonikos**  #1  (title:("premature infant*" OR "pre mature infant*" OR "preterm infant*" OR "pre term infant*" OR "infant born preterm" OR "infants born preterm" OR "premature neonat*" OR "pre mature neonat*" OR "preterm neonat*" OR "pre term neonat*" OR "premature neo nat*" OR "pre mature neo nat*" OR "preterm neo nat*" OR "pre term neo nat*" OR "premature baby*" OR "pre mature baby*" OR "preterm baby*" OR "pre term baby*" OR "premature babies" OR "pre mature babies" OR "preterm babies" OR "pre term babies" OR "premature newborn*" OR "pre mature newborn*" OR "preterm newborn*" OR "pre term newborn*" OR "premature new born*" OR "pre mature new born*" OR "preterm new born*" OR "pre term new born*" OR "premature newly born*" OR "pre mature newly born*" OR "preterm newly born*" OR "pre term newly born*" OR "premature child*" OR "pre mature child*" OR "preterm child*" OR "pre term child*" OR "child born preterm" OR "children born preterm" OR "children AND young people born preterm" OR "premature adolescen*" OR "pre mature adolescen*" OR "preterm adolescen*" OR "pre term adolescen*" OR "adolescent born preterm" OR "adolescents born preterm" OR "low birth weight" OR "low birthweight" OR "neonatal prematurity" OR "neo natal prematurity" OR "neonatal pre maturity" OR "neo natal pre maturity" OR "neonatal intensive care" OR "neo natal intensive care" OR preemie OR premie OR preemies OR premies) OR abstract:("premature infant*" OR "pre mature infant*" OR "preterm infant*" OR "pre term infant*" OR "infant born preterm" OR "infants born preterm" OR "premature neonat*" OR "pre mature neonat*" OR "preterm neonat*" OR "pre term neonat*" OR "premature neo nat*" OR "pre mature neo nat*" OR "preterm neo nat*" OR "pre term neo nat*" OR "premature baby*" OR "pre mature baby*" OR "preterm baby*" OR "pre term baby*" OR "premature babies" OR "pre mature babies" OR "preterm babies" OR "pre term babies" OR "premature newborn*" OR "pre mature newborn*" OR "preterm newborn*" OR "pre term newborn*" OR "premature new born*" OR "pre mature new born*" OR "preterm new born*" OR "pre term new born*" OR "premature newly born*" OR "pre mature newly born*" OR "preterm newly born*" OR "pre term newly born*" OR "premature child*" OR "pre mature child*" OR "preterm child*" OR "pre term child*" OR "child born preterm" OR "children born preterm" OR "children AND young people born preterm" OR "premature adolescen*" OR "pre mature adolescen*" OR "preterm adolescen*" OR "pre term adolescen*" OR "adolescent born preterm" OR "adolescents born preterm" OR "low birth weight" OR "low birthweight" OR "neonatal prematurity" OR "neo natal prematurity" OR "neonatal pre maturity" OR "neo natal pre maturity" OR "neonatal intensive care" OR "neo natal intensive care" OR preemie OR premie OR preemies OR premies))  #2  Filter: Systematic Reviews  #3  Filter (Publication Years): 2010 – 2023  #4 #1 AND #2 AND #3  = 2'592  Search results (Systematic Reviews) in all sources: 7'859  Search results (Systematic Reviews) after deduplication: 5'581 |
|  |

**Database: Ovid MEDLINE(R) ALL <1946 to May 06, 2025>**
**Search Strategy:**

**1**  exp Infant, Premature/ (69619)
**2**  exp Infant, low birth weight/ (40736)
**3**  Intensive Care, Neonatal/ (6245)
**4**  ((premature or pre mature or preterm or pre term) adj3 (infan* or neonat* or neo nat* or baby or babies or born? or newborn? or new born? or newly born? or child or children or adolescen*)).tw. (83031)
**5**  ((neonatal or neo natal) adj3 (prematurity or pre maturity)).tw. (714)
**6**  ((neonatal or neo natal) adj3 intensive care).tw. (29835)
**7**  (low birth weight or low birthweight).tw. (42977)
**8**  (preemie or premie or preemies or premies).tw. (240)
**9**  neonatal intensive care.tw. (29138)
**10**  or/1-9 (166352)
**11**  systematic reviews.jn. or search*.tw. or meta analysis.pt. or medline.tw. or systematic review.tw. (951259)
**12**  10 and 11 (7674)
**13**  limit 12 to yr="2010 -Current" (6354)
**14**  cochrane database of systematic reviews.jn. (17051)
**15**  13 and 14 (678)
