# Supplementary material for: Identification and Assessment of Systematic Reviews for Evidence‐Based Guideline Recommendations on Follow‐Up of Preterm Born Children: A Mapping Review
Source: Acta Paediatr. 2026 Apr 24;115(7):1384–99. doi: 10.1111/apa.70507 (PMC13250969; doi:10.1111/apa.70507)
Supplement: Supplementary file 3 — Appendix S3: Data extraction sheet. [file APA-115-1384-s003.docx]

Appendix S3: Data extraction sheet

We extracted the following criteria using Covidence.

General information

- Covidence ID
- Study ID (Author, Year)
- Title

Classification

- Time point of intervention
- Name/Details of Intervention

General information

- Search date
- Number of databases searched
- Objective of the Systematic Review
- Inclusion criteria
- Exclusion criteria
- Eligible/included study designs
- Total number of included studies

Type of aggregated evidence

For interventional systematic reviews

- Population description
- Preterm as full population or as subgroup
- Gestational age
- Pharmacologic interventions (substances)
- Intervention classification
- Route of intervention
- None-pharmacological intervention
- Type of comparator
- Type of outcome category
- Time point of outcome assessment

For prognostic reviews

- Population description
- Preterm as full population or as subgroup
- Gestational age
- Type of prognostic factor/model
- Type of comparator prognostic factor/model
- Type of outcome category
- Time point of outcome assessment
- Define firstly at what time points the prognostic factors (index and comparators) are to be used (that is, the time point of prognostication)
- Secondly over what time period the outcomes are predicted by these factors
- Define the intended setting and role of the prognostic factors

For diagnostic reviews

- Population description
- Preterm as full population or as subgroup
- Gestational age
- Index test
- Reference standard
- Target condition
- Time point of diagnosis

For prevalence/incidence reviews

- Condition
- Context
- Population
