# Supplementary material for: Identification and Assessment of Systematic Reviews for Evidence‐Based Guideline Recommendations on Follow‐Up of Preterm Born Children: A Mapping Review
Source: Acta Paediatr. 2026 Apr 24;115(7):1384–99. doi: 10.1111/apa.70507 (PMC13250969; doi:10.1111/apa.70507)
Supplement: Supplementary file 4 — Appendix S4: Characteristics of excluded studies. [file APA-115-1384-s005.docx]

Appendix S4: Characteristics of excluded studies

| **Author (Year)** | **Title** | **DOI** | **Reason of exclusion** |
| --- | --- | --- | --- |
| Acharya (2022) | Preterm Birth, Exasperation to the South Asian Countries |  | no systematic review |
| Allen (2010) | Inhaled nitric oxide in preterm infants |  | no systematic review |
| Altafirm (2023) | Prevention of Child Maltreatment: Integrative Review of Findings From an Evidence-Based Parenting Program | https://dx.doi.org/10.1177/15248380231201811 | no systematic review |
| Alvarez (2013) | Epidemiological and genetic characteristics associated with the severity of acute viral bronchiolitis by respiratory syncytial virus | https://dx.doi.org/10.1016/j.jped.2013.02.022 | no systematic review |
| Alves (2019) | Effects of physical activity on children's growth | https://dx.doi.org/10.1016/j.jped.2018.11.003 | no systematic review |
| Anderson (2017) | Burden of Severe Respiratory Syncytial Virus Disease Among 33-35 Weeks' Gestational Age Infants Born During Multiple Respiratory Syncytial Virus Seasons | https://dx.doi.org/10.1097/INF.0000000000001377 | no systematic review |
| Anonymous (2013) | Elective high-frequency oscillatory ventilation versus conventional ventilation for acute pulmonary dysfunction in preterm infants | https://dx.doi.org/10.1159/000338553 | no systematic review |
| Anonymous (2013) | Prophylactic Systemic Antifungal Agents to Prevent Mortality and Morbidity in Very Low Birth Weight Infants | 10.1159/000353683 | no systematic review |
| Askie (2018) | Association Between Oxygen Saturation Targeting and Death or Disability in Extremely Preterm Infants in the Neonatal Oxygenation Prospective Meta-analysis Collaboration | https://dx.doi.org/10.1001/jama.2018.5725 | no systematic review |
| Astuti (2022) | Oral feeding skills in premature infants: A concept analysis | https://dx.doi.org/10.33546/bnj.2107 | no systematic review |
| Bakker (2014) | Cerebrovascular function and cognition in childhood: a systematic review of transcranial Doppler studies | https://dx.doi.org/10.1186/1471-2377-14-43 | no systematic review |
| Bansal (2018) | The Laryngeal Mask Airway and Its Use in Neonatal Resuscitation: A Critical Review of Where We Are in 2017/2018 | https://dx.doi.org/10.1159/000481979 | no systematic review |
| Barre (2011) | Language abilities in children who were very preterm and/or very low birth weight: a meta-analysis | https://dx.doi.org/10.1016/j.jpeds.2010.10.032 | no systematic review |
| Bassareo (2010) | Biomarkers of corticosteroid-induced hypertrophic cardiomyopathy in preterm babies |  | no systematic review |
| Beyerlein (2010) | Infant formula supplementation with long-chain polyunsaturated fatty acids has no effect on Bayley developmental scores at 18 months of age--IPD meta-analysis of 4 large clinical trials | https://dx.doi.org/10.1097/MPG.0b013e3181acae7d | no systematic review |
| Bilgin (2021) | Subjective Well-Being and Self-Esteem in Preterm Born Adolescents: An Individual Participant Data Meta-Analysis | https://dx.doi.org/10.1097/DBP.0000000000000947 | no systematic review |
| Birere (2014) | An integrative review of factors that influence breastfeeding duration for premature infants after NICU hospitalization | https://dx.doi.org/10.1111/1552-6909.12297 | no systematic review |
| Blencowe (2013) | Preterm birth-associated neurodevelopmental impairment estimates at regional and global levels for 2010 | https://dx.doi.org/10.1038/pr.2013.204 | no systematic review |
| Blencowe (2013) | Preterm-associated visual impairment and estimates of retinopathy of prematurity at regional and global levels for 2010 | https://dx.doi.org/10.1038/pr.2013.205 | no systematic review |
| Blencowe (2015) | Preterm birth-associated neurodevelopmental impairment estimates at regional and global levels for 2010 |  | no systematic review |
| Bonan (2015) | Sleep deprivation, pain and prematurity: a review study | https://dx.doi.org/10.1590/0004-282X20140214 | no systematic review |
| Bos (2013) | Development of fine motor skills in preterm infants | https://dx.doi.org/10.1111/dmcn.12297 | no systematic review |
| Bowles (2016) | Infants With Technology Dependence: Facilitating the Road to Home |  | no systematic review |
| Boykova (2016) | Transition From Hospital to Home in Parents of Preterm Infants: A Literature Review |  | no systematic review |
| Braga (2012) | Strategies for implementing continuity of care after discharge of premature infants: an integrative review |  | no systematic review |
| Burdall (2019) | Neonatal skin care: Developments in care to maintain neonatal barrier function and prevention of diaper dermatitis | https://dx.doi.org/10.1111/pde.13714 | no systematic review |
| Burnett (2011) | Prevalence of psychiatric diagnoses in preterm and full-term children, adolescents and young adults: a meta-analysis | https://dx.doi.org/10.1017/S003329171100081X | no systematic review |
| Byrne (2017) | Implementation of Early Diagnosis and Intervention Guidelines for Cerebral Palsy in a High-Risk Infant Follow-Up Clinic | https://dx.doi.org/10.1016/j.pediatrneurol.2017.08.002 | no systematic review |
| Caporali (2020) | A global perspective on parental stress in the neonatal intensive care unit: a meta-analytic study | https://dx.doi.org/10.1038/s41372-020-00798-6 | no systematic review |
| Carbonell-Estrany (2013) | Effects of parental and household smoking on the risk of respiratory syncytial virus (RSV) hospitalisation in late-preterm infants and the potential impact of RSV prophylaxis | https://dx.doi.org/10.3109/14767058.2013.765850 | no systematic review |
| Carpay (2021) | Barriers and Facilitators to Breastfeeding in Moderate and Late Preterm Infants: A Systematic Review | https://dx.doi.org/10.1089/bfm.2020.0379 | no systematic review |
| Cassiano (2020) | Does preterm birth affect child temperament? A meta-analytic study | https://dx.doi.org/10.1016/j.infbeh.2019.101417 | no systematic review |
| Chiale (2021) | Complementary Feeding: Recommendations for the Introduction of Allergenic Foods and Gluten in the Preterm Infant | https://dx.doi.org/10.3390/nu13072477 | no systematic review |
| Chiappini (2019) | Update on vaccination of preterm infants: a systematic review about safety and efficacy/effectiveness. Proposal for a position statement by Italian Society of Pediatric Allergology and Immunology jointly with the Italian Society of Neonatology | https://dx.doi.org/10.1080/14760584.2019.1604230 | no systematic review |
| Christian (2013) | Risk of childhood undernutrition related to small-for-gestational age and preterm birth in low- and middle-income countries | https://dx.doi.org/10.1093/ije/dyt109 | no systematic review |
| Colin (2010) | Respiratory morbidity and lung function in preterm infants of 32 to 36 weeks' gestational age | https://dx.doi.org/10.1542/peds.2009-1381 | no systematic review |
| Conrad (2019) | Understanding the Pathophysiology, Implications, and Treatment Options of Patent Ductus Arteriosus in the Neonatal Population | https://dx.doi.org/10.1097/ANC.0000000000000590 | no systematic review |
| Cook (2010) | A systematic review and meta-analysis of perinatal variables in relation to the risk of testicular cancer--experiences of the son | https://dx.doi.org/10.1093/ije/dyq120 | no systematic review |
| Corwin (2018) | Bronchopulmonary dysplasia appropriateness as a surrogate marker for long-term pulmonary outcomes: A Systematic review | https://dx.doi.org/10.3233/NPM-181756 | no systematic review |
| Course (2019) | Fractional exhaled nitric oxide in preterm-born subjects: A systematic review and meta-analysis | https://dx.doi.org/10.1002/ppul.24270 | no systematic review |
| Cutrera (2019) | Impact of the 2014 American Academy of Pediatrics recommendation and of the resulting limited financial coverage by the Italian Medicines Agency for palivizumab prophylaxis on the RSV-associated hospitalizations in preterm infants during the 2016-2017 epidemic season: a systematic review of seven Italian reports | https://dx.doi.org/10.1186/s13052-019-0736-5 | no systematic review |
| D'Agostino (2010) | An evidentiary review regarding the use of chronological and adjusted age in the assessment of preterm infants | https://dx.doi.org/10.1111/j.1744-6155.2009.00215.x | no systematic review |
| DarcyMahoney (2017) | Leveraging the Skills of Nurses and the Power of Language Nutrition to Ensure a Better Future for Children | https://dx.doi.org/10.1097/ANC.0000000000000373 | no systematic review |
| denDekker (2016) | Early growth characteristics and the risk of reduced lung function and asthma: A meta-analysis of 25,000 children | https://dx.doi.org/10.1016/j.jaci.2015.08.050 | no systematic review |
| deOliveriaPeixoto (2016) | Reviewing the use of corticosteroids in bronchopulmonary dysplasia | https://dx.doi.org/10.1016/j.jped.2015.07.007 | no systematic review |
| Doyle (2019) | Expiratory airflow in late adolescence and early adulthood in individuals born very preterm or with very low birthweight compared with controls born at term or with normal birthweight: a meta-analysis of individual participant data | https://dx.doi.org/10.1016/S2213-2600(18)30530-7 | no systematic review |
| Duess (2014) | Prevalence of Hirschsprung's disease in premature infants: a systematic review | https://dx.doi.org/10.1007/s00383-014-3540-8 | no systematic review |
| Edwards (2014) | Higher systolic blood pressure with normal vascular function measurements in preterm-born children | https://dx.doi.org/10.1111/apa.12699 | no systematic review |
| Einspieler (2016) | The General Movement Assessment Helps Us to Identify Preterm Infants at Risk for Cognitive Dysfunction | https://dx.doi.org/10.3389/fpsyg.2016.00406 | no systematic review |
| Evensen (2022) | Multidisciplinary and neuroimaging findings in preterm born very low birthweight individuals from birth to 28 years of age: A systematic review of a Norwegian prospective cohort study | https://dx.doi.org/10.1111/ppe.12890 | no systematic review |
| Farajdokht (2017) | Very low birth weight is associated with brain structure abnormalities and cognitive function impairments: A systematic review | https://dx.doi.org/10.1016/j.bandc.2017.07.006 | no systematic review |
| Finch (2015) | Review of trace mineral requirements for preterm infants: what are the current recommendations for clinical practice? | 10.1177/0884533614563353 | no systematic review |
| Fitzgerald (2017) | Body structure, function, activity and participation in preschool aged children born preterm. A systematic review and meta-analysis using the international classification of functioning, disability and health framework | 10.1111/dmcn.13-13512 | no systematic review |
| Flint (2018) | The use of overnight oximetry in neonates: A literature review | https://dx.doi.org/10.1111/jpc.13935 | no systematic review |
| FrolekClark (2013) | Systematic review of occupational therapy interventions to improve cognitive development in children ages birth-5 years | https://dx.doi.org/10.5014/ajot.2013.006163 | no systematic review |
| Fuentefria (2017) | Motor development of preterm infants assessed by the Alberta Infant Motor Scale: systematic review article | https://dx.doi.org/10.1016/j.jped.2017.03.003 | no systematic review |
| Gawlik (2020) | The Influence of DHA on Language Development: A Review of Randomized Controlled Trials of DHA Supplementation in Pregnancy, the Neonatal Period, and Infancy | https://dx.doi.org/10.3390/nu12103106 | no systematic review |
| Ghavam (2014) | Effects of placental transfusion in extremely low birthweight infants: meta-analysis of long- and short-term outcomes |  | no systematic review |
| Gilarska (2019) | Extremely Low Birth Weight Predisposes to Impaired Renal Health: A Pooled Analysis | https://dx.doi.org/10.1159/000502715 | no systematic review |
| Groot (2022) | Meta-analysis of ocular axial length in newborns and infants up to 3 years of age | https://dx.doi.org/10.1016/j.survophthal.2021.05.010 | no systematic review |
| Grunberg (2019) | NICU infant health severity and family outcomes: a systematic review of assessments and findings in psychosocial research | https://dx.doi.org/10.1038/s41372-018-0282-9 | no systematic review |
| Guiducci (2022) | Neonatal Hyperglycemia and Neurodevelopmental Outcomes in Preterm Infants: A Review | https://dx.doi.org/10.3390/children9101541 | no systematic review |
| Guillen (2012) | Relationship between attrition and neurodevelopmental impairment rates in extremely preterm infants at 18 to 24 months: a systematic review | https://dx.doi.org/10.1001/archpediatrics.2011.616 | no systematic review |
| Guimares (2013) | Reaching behavior in preterm infants during the first year of life: a systematic review |  | no systematic review |
| Ibrahim (2018) | Brain imaging in preterm infants <32 weeks gestation: a clinical review and algorithm for the use of cranial ultrasound and qualitative brain MRI | https://dx.doi.org/10.1038/s41390-018-0194-6 | no systematic review |
| Ishii (2013) | Outcomes of infants born at 22 and 23 weeks' gestation | https://dx.doi.org/10.1542/peds.2012-2857 | no systematic review |
| Jaekel (2022) | Mathematical performance in childhood and early adult outcomes after very preterm birth: an individual participant data meta-analysis | https://dx.doi.org/10.1111/dmcn.15132 | no systematic review |
| Jarjour (2015) | Neurodevelopmental outcome after extreme prematurity: a review of the literature | https://dx.doi.org/10.1016/j.pediatrneurol.2014.10.027 | no systematic review |
| Jenkinson (2023) | Systematic review of the long-term effects of postnatal corticosteroids | https://dx.doi.org/10.1515/jpm-2023-0297 | no systematic review |
| Jones (2020) | Contemporary Outcomes for Infants with Necrotizing Enterocolitis-A Systematic Review | https://dx.doi.org/10.1016/j.jpeds.2019.11.011 | no systematic review |
| Juneau (2015) | Review and Critical Analysis of Massage Studies for Term and Preterm Infants | https://dx.doi.org/10.1891/0730-0832.34.3.165 | no systematic review |
| Kandasamy (2012) | Oligonephropathy of prematurity | https://dx.doi.org/10.1055/s-0031-1295651 | no systematic review |
| Karkhaneh (2020) | Adverse events associated with paediatric massage therapy: a systematic review | https://dx.doi.org/10.1136/bmjpo-2019-000584 | no systematic review |
| Kavsek (2010) | Visual habituation and dishabituation in preterm infants: a review and meta-analysis | https://dx.doi.org/10.1016/j.ridd.2010.04.016 | no systematic review |
| Keunen (2012) | Brain tissue volumes in preterm infants: prematurity, perinatal risk factors and neurodevelopmental outcome: a systematic review | https://dx.doi.org/10.3109/14767058.2012.664343 | no systematic review |
| Korja (2012) | The effects of preterm birth on mother-infant interaction and attachment during the infant's first two years | https://dx.doi.org/10.1111/j.1600-0412.2011.01304.x | no systematic review |
| Kosciolek (2022) | Systematic review of the guidelines for retinopathy of prematurity | https://dx.doi.org/10.1177/11206721221126286 | no systematic review |
| Kouzouna (2016) | A systematic review of early life factors which adversely affect subsequent lung function | https://dx.doi.org/10.1016/j.prrv.2016.03.003 | no systematic review |
| Kovachy (2015) | Reading abilities in school-aged preterm children: a review and meta-analysis | https://dx.doi.org/10.1111/dmcn.12652 | no systematic review |
| Kraljevic (2012) | Early erythropoietin for preventing red blood cell transfusion in preterm and/or low birth weight infants | https://dx.doi.org/10.1002/14651858.CD004863.pub3 | no systematic review |
| Kurath (2010) | Transmission of cytomegalovirus via breast milk to the prematurely born infant: a systematic review | https://dx.doi.org/10.1111/j.1469-0691.2010.03140.x | no systematic review |
| Legge (2023) | Examining the impact of premature birth on parental mental health and family functioning in the years following hospital discharge: A review | https://dx.doi.org/10.3233/NPM-221107 | no systematic review |
| Liotto (2020) | Complementary Feeding in Preterm Infants: A Systematic Review | https://dx.doi.org/10.3390/nu12061843 | no systematic review |
| Lucas (2020) | Comment on "Human Milk-Derived Fortifiers Compared with Bovine Milk-Derived Fortifiers in Preterm Infants: A Systematic Review and Meta-Analysis" | https://dx.doi.org/10.1093/advances/nmaa098 | no systematic review |
| Machado (2017) | SENSORY PROCESSING DURING CHILDHOOD IN PRETERM INFANTS: A SYSTEMATIC REVIEW | https://dx.doi.org/10.1590/1984-0462/;2017;35;1;00008 | no systematic review |
| MachadoJunior (2014) | Late prematurity: a systematic review | https://dx.doi.org/10.1016/j.jped.2013.08.012 | no systematic review |
| Maitra (2014) | Difficulty in mental, neuromusculoskeletal, and movement-related school functions associated with low birthweight or preterm birth: a meta-analysis | https://dx.doi.org/10.5014/ajot.2014.009985 | no systematic review |
| Manja (2017) | Oxygen Saturation Targets in Preterm Infants and Outcomes at 18-24 Months: A Systematic Review | https://dx.doi.org/10.1542/peds.2016-1609 | no systematic review |
| Mansfield (2023) | Impact of perinatal factors on biomarkers of cardiovascular disease risk in preadolescent children | https://dx.doi.org/10.1097/HJH.0000000000003452 | no systematic review |
| Meher (2015) | Impact of cerebral redistribution on neurodevelopmental outcome in small-for-gestational-age or growth-restricted babies: a systematic review | https://dx.doi.org/10.1002/uog.14818 | no systematic review |
| Mwaniki (2012) | Long-term neurodevelopmental outcomes after intrauterine and neonatal insults: a systematic review | https://dx.doi.org/10.1016/S0140-6736(11)61577-8 | no systematic review |
| Nelson (2023) | Parental cognitive stimulation in preterm-born children's neurocognitive functioning during the preschool years: a systematic review | https://dx.doi.org/10.1038/s41390-023-02642-x | no systematic review |
| Onland (2010) | Open-label glucocorticoids modulate dexamethasone trial results in preterm infants | https://dx.doi.org/10.1542/peds.2010-0597 | no systematic review |
| Pandit (2013) | Diffusion magnetic resonance imaging in preterm brain injury | https://dx.doi.org/10.1007/s00234-013-1242-x | no systematic review |
| Parikh (2016) | Advanced neuroimaging and its role in predicting neurodevelopmental outcomes in very preterm infants | https://dx.doi.org/10.1053/j.semperi.2016.09.005 | no systematic review |
| Pino (2023) | Effect of Musical Stimulation on Placental Programming and Neurodevelopment Outcome of Preterm Infants: A Systematic Review | https://dx.doi.org/10.3390/ijerph20032718 | no systematic review |
| Pinto (2021) | Post-Hemorrhagic Hydrocephalus and Outcomes Amongst Neonates With Intraventricular Hemorrhage: A Systematic Review and Pooled Analysis | https://dx.doi.org/10.7759/cureus.18877 | no systematic review |
| Pisani (2020) | Neonatal seizures in preterm infants: A systematic review of mortality risk and neurological outcomes from studies in the 2000's | https://dx.doi.org/10.1016/j.seizure.2019.12.005 | no systematic review |
| Plaisier (2014) | Optimal timing of cerebral MRI in preterm infants to predict long-term neurodevelopmental outcome: a systematic review | https://dx.doi.org/10.3174/ajnr.A3513 | no systematic review |
| Pons (2011) | Meta-analysis of passive immunoprophylaxis in paediatric patients at risk of severe RSV infection | https://dx.doi.org/10.1111/j.1651-2227.2010.02059.x | no systematic review |
| Potsmesilova (2023) | Basal Stimulation as Developmental Support in At-Risk Newborns: A Literature Review | https://dx.doi.org/10.3390/children10020389 | no systematic review |
| Prescott (2010) | Cost effectiveness of respiratory syncytial virus prophylaxis: a critical and systematic review | https://dx.doi.org/10.2165/11531860-000000000-00000 | no systematic review |
| Radtka (2011) | The paradox of breastfeeding-associated morbidity among late preterm infants | https://dx.doi.org/10.1111/j.1552-6909.2010.01211.x | no systematic review |
| Rana (2020) | Feeding Interventions for Infants with Growth Failure in the First Six Months of Life: A Systematic Review | https://dx.doi.org/10.3390/nu12072044 | no systematic review |
| Resch (2016) | Prematurity and the burden of influenza and respiratory syncytial virus disease | https://dx.doi.org/10.1007/s12519-015-0055-x | no systematic review |
| Robinson (2017) | Lenticulostriate Vasculopathy and Neurodevelopmental Outcomes in Preterm Infants: A Systematic Review | https://dx.doi.org/10.1055/s-0037-1598245 | no systematic review |
| Romeo (2020) | Early Neurological Assessment and Long-Term Neuromotor Outcomes in Late Preterm Infants: A Critical Review | https://dx.doi.org/10.3390/medicina56090475 | no systematic review |
| Ross (2013) | Feeding Outcomes in Preterm Infants After Discharge From the Neonatal Intensive Care Unit (NICU): A Systematic Review | 10.1053/j.nainr.2013.04.003 | no systematic review |
| Sacchi (2020) | Association of Intrauterine Growth Restriction and Small for Gestational Age Status With Childhood Cognitive Outcomes: A Systematic Review and Meta-analysis | https://dx.doi.org/10.1001/jamapediatrics.2020.1097 | no systematic review |
| Salinas-Escudero (2012) | Cost-effectiveness analysis of the use of palivizumab in the prophylaxis of preterm patients in Mexico |  | no systematic review |
| Sarda (2021) | Global prevalence of long-term neurodevelopmental impairment following extremely preterm birth: a systematic literature review | https://dx.doi.org/10.1177/03000605211028026 | no systematic review |
| Shaffer (2019) | Effect of Prophylaxis for Early Adrenal Insufficiency Using Low-Dose Hydrocortisone in Very Preterm Infants: An Individual Patient Data Meta-Analysis | https://dx.doi.org/10.1016/j.jpeds.2018.10.004 | no systematic review |
| Shepherd (2017) | Antenatal and intrapartum interventions for preventing cerebral palsy: an overview of Cochrane systematic reviews | https://dx.doi.org/10.1002/14651858.CD012077.pub2 | no systematic review |
| Solaski (2014) | Contribution of socio-economic status on the prevalence of cerebral palsy: a systematic search and review | https://dx.doi.org/10.1111/dmcn.12456 | no systematic review |
| Soleimani (2014) | Long-term neurodevelopmental outcomes after preterm birth | https://dx.doi.org/10.5812/ircmj.17965 | no systematic review |
| Sonnenschein-vanderVoort (2014) | Preterm birth, infant weight gain, and childhood asthma risk: a meta-analysis of 147,000 European children | https://dx.doi.org/10.1016/j.jaci.2013.12.1082 | no systematic review |
| Stein (2017) | Respiratory syncytial virus hospitalization and mortality: Systematic review and meta-analysis | https://dx.doi.org/10.1002/ppul.23570 | no systematic review |
| Talladini (2015) | Systematic and Meta-Analytic Review: Triggering Agents of Parental Perception of Child's Vulnerability in Instances of Preterm Birth | https://dx.doi.org/10.1093/jpepsy/jsv010 | no systematic review |
| Tan (2019) | Development of refractive error in children treated for retinopathy of prematurity with anti-vascular endothelial growth factor (anti-VEGF) agents: A meta-analysis and systematic review | https://dx.doi.org/10.1371/journal.pone.0225643 | no systematic review |
| Teller (2016) | Post-discharge formula feeding in preterm infants: A systematic review mapping evidence about the role of macronutrient enrichment | https://dx.doi.org/10.1016/j.clnu.2015.08.006 | no systematic review |
| Teune (2011) | A systematic review of severe morbidity in infants born late preterm | https://dx.doi.org/10.1016/j.ajog.2011.07.015 | no systematic review |
| Toscano (2020) | Controlling Parenting Behaviors in Parents of Children Born Preterm: A Meta-Analysis | https://dx.doi.org/10.1097/DBP.0000000000000762 | no systematic review |
| Valeri (2015) | Neonatal pain and developmental outcomes in children born preterm: a systematic review | https://dx.doi.org/10.1097/AJP.0000000000000114 | no systematic review |
| Vanthienen (2023) | The QTc-Bazett Interval in Former Very Preterm Infants in Adolescence and Young Adulthood is Not Different from Term-Born Controls | https://dx.doi.org/10.1007/s40264-023-01335-y | no systematic review |
| Vieria (2011) | Developmental outcomes and quality of life in children born preterm at preschool- and school-age | https://dx.doi.org/10.2223/JPED.2096 | no systematic review |
| Vinther (2023) | Gestational age at birth and body size from infancy through adolescence: An individual participant data meta-analysis on 253,810 singletons in 16 birth cohort studies | https://dx.doi.org/10.1371/journal.pmed.1004036 | no systematic review |
| Vittner (2016) | The Neonatal Intensive Care Unit Network Neurobehavioral Scale Use with High-Risk Infants: An Integrative Review | 10.1097/NNR.0000000000000152 | no systematic review |
| Webb (2022) | Neurodevelopmental Outcomes Associated With Intravitreal Bevacizumab Injections for Retinopathy of Prematurity | https://dx.doi.org/10.1097/ANC.0000000000000881 | no systematic review |
| Williams (2010) | Prevalence of motor-skill impairment in preterm children who do not develop cerebral palsy: a systematic review | https://dx.doi.org/10.1111/j.1469-8749.2009.03544.x | no systematic review |
| Yates (2012) | Postnatal intravenous steroids and long-term neurological outcome: recommendations from meta-analyses | https://dx.doi.org/10.1136/adc.2010.208868 | no systematic review |
| Zemmgulyte (2019) | Influence of preterm birth for child's oral health status |  | no systematic review |
| Zerbeto (2015) | Association between gestational age and birth weight on the language development of Brazilian children: a systematic review | https://dx.doi.org/10.1016/j.jped.2014.11.003 | no systematic review |
| Zimmermann (2018) | Do Infants Born Very Premature and Who Have Very Low Birth Weight Catch Up With Their Full Term Peers in Their Language Abilities by Early School Age? | https://dx.doi.org/10.1044/2017_JSLHR-L-16-0150 | no systematic review |
| Appello (2014) | The newborn individualized developmental care and assessment program for preterm infants: A meta-analysis of efficacy |  | Dissertation |
| SukYin (2017) | The Effectiveness of a Guided Participation Discharge Programme on Improving Parental Outcomes for Very Premature Infants: A Systematic Review and Pilot Randomized Controlled Trial |  | Dissertation |
| Edmond (2022) | Evidence for Global Health Care Interventions for Preterm or Low Birth Weight Infants: An Overview of Systematic Reviews | https://dx.doi.org/10.1542/peds.2022-057092C | umbrella review |
| Gonzalez (2022) | Anti-vascular endothelial growth factor (VEGF) drugs compared to laser photocoagulation for treatment of type 1 retinopathy of prematurity | https://dx.doi.org/10.5867/medwave.2022.01.8507 | umbrella review |
| Haller (2016) | Neurological sequelae of healthcare-associated sepsis in very-low-birthweight infants: Umbrella review and evidence-based outcome tree | https://dx.doi.org/10.2807/1560-7917.ES.2016.21.8.30143 | umbrella review |
| Kelly (2020) | The Influence of preterm birth beyond infancy: Umbrella review of outcomes of adolescents and adults born preterm | https://dx.doi.org/10.1097/JXX.0000000000000248 | umbrella review |
| Liu (2022) | Effectiveness of feeding supplementation in preterm infants: an overview of systematic reviews | https://dx.doi.org/10.1186/s12887-021-03052-w | umbrella review |
| Puthussery (2018) | Effectiveness of early intervention programs for parents of preterm infants: a meta-review of systematic reviews | https://dx.doi.org/10.1186/s12887-018-1205-9 | umbrella review |
| Shepherd (2018) | Neonatal interventions for preventing cerebral palsy: an overview of Cochrane Systematic Reviews | https://dx.doi.org/10.1002/14651858.CD012409.pub2 | umbrella review |
| AguilarCordero (2014) | [Effect of nutrition on growth and neurodevelopment in the preterm infant: a systematic review] | https://dx.doi.org/10.3305/nh.2015.31.2.8266 | wrong language |
| AmaralMartins (2012) | INSERTION OF THE FAMILY IN THE NEONATAL INTENSIVE CARE UNIT: A SYSTEMATIC REVIEW | 10.5205/reuol.2226-17588-1-LE.0604201225 | wrong language |
| Beaujou (2019) | Efectos del masaje sobre el peso y el desarrollo motor del recién nacido prematuro: revisión sistemática | 10.1016/j.ft.2019.07.004 | wrong language |
| Custódio (2014) | Social support networks in the context of prematurity: perspective of the bioecological model of human development |  | wrong language |
| Englerova (2020) | The effects of prenatal, perinatal and neonatal factors on academic performance in primary school age children |  | wrong language |
| Fernandes (2017) | Neuropsychomotor development of premature newborns: a systematic review |  | wrong language |
| Gascoin (2013) | Long-term outcome in context of intra uterine growth restriction and/or small for gestational age newborns | https://dx.doi.org/10.1016/j.jgyn.2013.09.014 | wrong language |
| Myrhaug (2017) | Prognosis and Follow-Up of Extreme Preterm Infants: A Systematic Review |  | wrong language |
| Oliveira (2015) | Near-infrared spectroscopy as an auxiliary tool in the study of child development | https://dx.doi.org/10.1016/j.rpped.2015.03.003 | wrong language |
| Peinado-Gorlat (2020) | General movement assessment as a tool for determining the prognosis in infantile cerebral palsy in preterm infants: a systematic review | https://dx.doi.org/10.33588/rn.7104.2019460 | wrong language |
| Podporina (2022) | Eating behavior and skills of premature children in different age periods | https://dx.doi.org/10.33029/0042-8833-2022-91-1-19-26 | wrong language |
| Rechia (2016) | Effects of prematurity on language acquisition and auditory maturation: a systematic review | https://dx.doi.org/10.1590/2317-1782/20162015218 | wrong language |
| SerranoGomez (2020) | Characteristics of motor behaviour in premature infants during the first months of postnatal life. A literature review | https://dx.doi.org/10.1016/j.rh.2019.09.004 | wrong language |
| TavaresSilvaFernandes (2017) | Desenvolvimento neuropsicomotor de recém-nascidos prematuros: uma revisão sistemática | 10.5585/ConsSaude.v16n4.7835 | wrong language |
| Wachholtz (2016) | Early intervention in infants at high-risk of developing cerebral palsy: a systematic review |  | wrong language |
| Xiang (2022) | Effect of prophylactic use of hydrolyzed protein formula on gastrointestinal diseases and physical growth in preterm infants: a Meta analysis | https://dx.doi.org/10.7499/j.issn.1008-8830.2109124 | wrong language |
| Zhang (2021) | Risk factors for neonatal congenital hypothyroidism: a Meta analysis |  | wrong language |
| Badhiwala (2015) | Treatment of posthemorrhagic ventricular dilation in preterm infants: a systematic review and meta-analysis of outcomes and complications | https://dx.doi.org/10.3171/2015.3.PEDS14630 | Wrong study design |
| Hurt (2023) | What matters to families about the healthcare of preterm or low birth weight infants: A qualitative evidence synthesis | https://dx.doi.org/10.1016/j.pec.2023.107893 | Wrong study design |
| Imdad, Aamer (2021) | Effects of neonatal nutrition interventions on neonatal mortality and child health and development outcomes: A systematic review | https://dx.doi.org/10.1002/cl2.1141 | Wrong setting |
| Bauer (2011) | Meta-analysis of hemorrhagic complications from ventriculostomy placement by neurosurgeons | https://dx.doi.org/10.1227/NEU.0b013e31821a45ba | Wrong patient population |
| Carelli (2022) | Gaze-Contingent Eye-Tracking Training in Brain Disorders: A Systematic Review | https://dx.doi.org/10.3390/brainsci12070931 | Wrong patient population |
| Catania (2019) | Risk Factors for Surgical Site Infection in Neonates: A Systematic Review of the Literature and Meta-Analysis | https://dx.doi.org/10.3389/fped.2019.00101 | Wrong patient population |
| Conde-Agudelo (2016) | Kangaroo mother care to reduce morbidity and mortality in low birthweight infants | https://dx.doi.org/10.1002/14651858.CD002771.pub4 | Wrong patient population |
| Curioni, Cintia C. (2022) | Effectiveness of nutritional interventions to prevent nonprogressive congenital and perinatal brain injuries: a systematic review and meta-analysis of randomized trials | https://dx.doi.org/10.1093/nutrit/nuac028 | Wrong patient population |
| Fenton (2014) | Higher versus lower protein intake in formula-fed low birth weight infants | https://dx.doi.org/10.1002/14651858.CD003959.pub3 | Wrong patient population |
| Hunt (2019) | "They've walked the walk": A systematic review of quantitative and qualitative evidence for parent-to-parent support for parents of babies in neonatal care | 10.1016/j.jnn.2019.03.011 | Wrong patient population |
| Kraljevic (2013) | Early educational and behavioral RCT interventions to reduce maternal symptoms of psychological trauma following preterm birth: a systematic review | https://dx.doi.org/10.1097/JPN.0b013e3182a8bfe2 | Wrong patient population |
| Lucas (2016) | Interventions to improve gross motor performance in children with neurodevelopmental disorders: a meta-analysis |  | Wrong patient population |
| Martinez-Shaw (2023) | Effective stress intervention programs for parents of premature children: A systematic review | https://dx.doi.org/10.1002/smi.3194 | Wrong patient population |
| McDonald (2013) | Effect of timing of umbilical cord clamping of term infants on maternal and neonatal outcomes | https://dx.doi.org/10.1002/14651858.CD004074.pub3 | Wrong patient population |
| McDonald (2014) | Effect of timing of umbilical cord clamping of term infants on maternal and neonatal outcomes | https://dx.doi.org/10.1002/ebch.1971 | Wrong patient population |
| McKeown (2023) | The Prevalence of PTSD of Mothers and Fathers of High-Risk Infants Admitted to NICU: A Systematic Review | https://dx.doi.org/10.1007/s10567-022-00421-4 | Wrong patient population |
| Mitra (2023) | Interventions for patent ductus arteriosus (PDA) in preterm infants: an overview of Cochrane Systematic Reviews | https://dx.doi.org/10.1002/14651858.CD013588.pub2 | Wrong patient population |
| Mutua (2020) | Effects of vitamin D deficiency on neurobehavioural outcomes in children: a systematic review | https://dx.doi.org/10.12688/wellcomeopenres.15730.1 | Wrong patient population |
| Okwundu (2012) | Prophylactic phototherapy for preventing jaundice in preterm or low birth weight infants | https://dx.doi.org/10.1002/14651858.CD007966.pub2 | Wrong patient population |
| Pathak (2023) | Effects of kangaroo mother care on maternal and paternal health: systematic review and meta-analysis | https://dx.doi.org/10.2471/BLT.22.288977 | Wrong patient population |
| Wolf (2020) | Magnesium sulphate for fetal neuroprotection at imminent risk for preterm delivery: a systematic review with meta-analysis and trial sequential analysis | https://dx.doi.org/10.1111/1471-0528.16238 | Wrong patient population |
| AlEthawi (2012) | Volume-targeted versus Pressure-limited Ventilation for Preterm Infants: A Systematic Review and Meta-Analysis |  | Wrong outcomes |
| Alnuaimi (2022) | Father's Bonding With an Infant Born Prematurely: A Qualitative Meta-synthesis | https://dx.doi.org/10.1177/01939459211002909 | Wrong outcomes |
| Alves (2020) | Impact of the kangaroo method of breastfeeding of preterm newborn infants in Brazil: an integrative review |  | Wrong outcomes |
| Arvedson (2010) | Evidence-based systematic review: effects of oral motor interventions on feeding and swallowing in preterm infants | https://dx.doi.org/10.1044/1058-0360(2010/09-0067) | Wrong outcomes |
| Beltram (2022) | Sensory-based interventions in the NICU: systematic review of effects on preterm brain development | https://dx.doi.org/10.1038/s41390-021-01718-w | Wrong outcomes |
| Boswinkel (2020) | A systematic review on brain injury and altered brain development in moderate-late preterm infants | https://dx.doi.org/10.1016/j.earlhumdev.2020.105094 | Wrong outcomes |
| Brett (2011) | A systematic mapping review of effective interventions for communicating with, supporting and providing information to parents of preterm infants | https://dx.doi.org/10.1136/bmjopen-2010-000023 | Wrong outcomes |
| Buffone (2022) | Osteopathic Treatment for Gastrointestinal Disorders in Term and Preterm Infants: A Systematic Review and Meta-Analysis | https://dx.doi.org/10.3390/healthcare10081525 | Wrong outcomes |
| Checchia, Paul A. (2011) | Mortality and morbidity among infants at high risk for severe respiratory syncytial virus infection receiving prophylaxis with palivizumab: a systematic literature review and meta-analysis | https://dx.doi.org/10.1097/PCC.0b013e3182070990 | Wrong outcomes |
| deGroot (2021) | The value of cardiorespiratory parameters for sleep state classification in preterm infants: A systematic review | https://dx.doi.org/10.1016/j.smrv.2021.101462 | Wrong outcomes |
| Delanerolle (2022) | Mental health impact on Black, Asian and Minority Ethnic populations with preterm birth: A systematic review and meta-analysis | https://dx.doi.org/10.5498/wjp.v12.i9.1233 | Wrong outcomes |
| Dohms (2019) | Inguinal hernia repair in preterm neonates: is there evidence that spinal or general anaesthesia is the better option regarding intraoperative and postoperative complications? A systematic review and meta-analysis | https://dx.doi.org/10.1136/bmjopen-2018-028728 | Wrong outcomes |
| Fan (2023) | Parent-Performed Infant Massage for Improving Parental Mental State Within 18 Months Postpartum: A Systematic Review | https://dx.doi.org/10.3928/02793695-20220906-04 | Wrong outcomes |
| Feng (2021) | Psychological or educational eHealth interventions on depression, anxiety or stress following preterm birth: a systematic review | https://dx.doi.org/10.1080/02646838.2020.1750576 | Wrong outcomes |
| Freitas (2014) | Changes in physiological and behavioral parameters of preterm infants undergoing body hygiene: a systematic review | https://dx.doi.org/10.1590/S0080-623420140000600025 | Wrong outcomes |
| Fry (2018) | Systematic Review of Quality Improvement Initiatives Related to Cue-Based Feeding in Preterm Infants | https://dx.doi.org/10.1016/j.nwh.2018.07.006 | Wrong outcomes |
| Girabent-Farres (2021) | Effects of early intervention on parenting stress after preterm birth: A meta-analysis | https://dx.doi.org/10.1111/cch.12853 | Wrong outcomes |
| Gonzales, Tara (2023) | Effectiveness and Safety of Palivizumab for the Prevention of Serious Lower Respiratory Tract Infection Caused by Respiratory Syncytial Virus: A Systematic Review | https://dx.doi.org/10.1055/a-1990-2633 | Wrong outcomes |
| Grassi (2019) | Early Intervention to Improve Sucking in Preterm Newborns: A Systematic Review of Quantitative Studies | https://dx.doi.org/10.1097/ANC.0000000000000594 | Wrong outcomes |
| Grassi (2019) | Early Intervention to Improve Sucking in Preterm Newborns: A Systematic Review of Quantitative Studies | https://dx.doi.org/10.1097/ANC.0000000000000543 | Wrong outcomes |
| Grzeskowiak (2018) | Domperidone for increasing breast milk volume in mothers expressing breast milk for their preterm infants: a systematic review and meta-analysis | https://dx.doi.org/10.1111/1471-0528.15177 | Wrong outcomes |
| Hashemipour (2018) | Screening of congenital hypothyroidism in preterm, low birth weight and very low birth weight neonates: A systematic review | https://dx.doi.org/10.1016/j.pedneo.2017.04.006 | Wrong outcomes |
| Henry (2019) | Incidence, Risk Factors, and Comorbidities of Vocal Cord Paralysis After Surgical Closure of a Patent Ductus Arteriosus: A Meta-analysis | https://dx.doi.org/10.1007/s00246-018-1967-8 | Wrong outcomes |
| Homaira, Nusrat (2014) | Effectiveness of Palivizumab in Preventing RSV Hospitalization in High Risk Children: A Real-World Perspective | https://dx.doi.org/10.1155/2014/571609 | Wrong outcomes |
| IgualBlasco (2023) | Effects of Chest Physiotherapy in Preterm Infants with Respiratory Distress Syndrome: A Systematic Review | https://dx.doi.org/10.3390/healthcare11081091 | Wrong outcomes |
| Ikonen (2015) | Preterm Infants' Mothers' Experiences With Milk Expression and Breastfeeding: An Integrative Review | https://dx.doi.org/10.1097/ANC.0000000000000232 | Wrong outcomes |
| Kotb (2021) | Intussusception in preterm neonates: A systematic review of a rare condition | https://dx.doi.org/10.1186/s12887-021-03065-5 | Wrong outcomes |
| Mac, Stephen (2019) | Cost-effectiveness of Palivizumab for Respiratory Syncytial Virus: A Systematic Review | https://dx.doi.org/10.1542/peds.2018-4064 | Wrong outcomes |
| Mascarenhas (2023) | Multisystem inflammatory syndrome in neonates (MIS-N): a systematic review | https://dx.doi.org/10.1007/s00431-023-04906-4 | Wrong outcomes |
| Mirghafourvand (2017) | The Effect of Creating Opportunities for Parent Empowerment Program on Parent's Mental Health: A Systematic Review | 10.5812/ijp.5704 | Wrong outcomes |
| Nguyen (2023) | Prevalence of and factors associated with postnatal depression and anxiety among parents of preterm infants: A systematic review and meta-analysis | https://dx.doi.org/10.1016/j.jad.2022.11.015 | Wrong outcomes |
| Olchanski, Natalia (2018) | Palivizumab Prophylaxis for Respiratory Syncytial Virus: Examining the Evidence Around Value | https://dx.doi.org/10.1093/ofid/ofy031 | Wrong outcomes |
| ParnellPrevost (2019) | Manual therapy for the pediatric population: a systematic review | https://dx.doi.org/10.1186/s12906-019-2447-2 | Wrong outcomes |
| Provenzi (2018) | Preterm behavioral epigenetics: A systematic review | https://dx.doi.org/10.1016/j.neubiorev.2017.08.020 | Wrong outcomes |
| Relland (2021) | Regional anesthesia in neonates and infants outside the immediate perioperative period: A systematic review of studies with efficacy and safety considerations | https://dx.doi.org/10.1111/pan.14042 | Wrong outcomes |
| Simoes, Eric A. F. (2018) | Past, Present and Future Approaches to the Prevention and Treatment of Respiratory Syncytial Virus Infection in Children | https://dx.doi.org/10.1007/s40121-018-0188-z | Wrong outcomes |
| VanderCruyssen (2015) | The voiding pattern in healthy pre- and term infants and toddlers: a literature review | https://dx.doi.org/10.1007/s00431-015-2578-5 | Wrong outcomes |
| Visser (2012) | The validity of biochemical markers in metabolic bone disease in preterm infants: a systematic review | https://dx.doi.org/10.1111/j.1651-2227.2012.02626.x | Wrong outcomes |
| Wang (2013) | The efficacy of massage on preterm infants: a meta-analysis | https://dx.doi.org/10.1055/s-0032-1332801 | Wrong outcomes |
| Yang (2018) | Effect of different doses of vitamin D supplementation on preterm infants - an updated meta-analysis | https://dx.doi.org/10.1080/14767058.2017.1363731 | Wrong outcomes |
| Zhang (2021) | Empowerment programs for parental mental health of preterm infants: A meta-analysis | https://dx.doi.org/10.1016/j.pec.2021.01.021 | Wrong outcomes |
| Zhong (2019) | Early Intratracheal Administration of Corticosteroid and Pulmonary Surfactant for Preventing Bronchopulmonary Dysplasia in Preterm Infants with Neonatal Respiratory Distress Syndrome: A Meta-analysis | https://dx.doi.org/10.1007/s11596-019-2064-9 | Wrong outcomes |
| Badurdeen, Shiraz (2019) | Safety and Immunogenicity of Early Bacillus Calmette-Guerin Vaccination in Infants Who Are Preterm and/or Have Low Birth Weights: A Systematic Review and Meta-analysis | https://dx.doi.org/10.1001/jamapediatrics.2018.4038 | Wrong intervention |
| Humayun (2021) | Systematic review of the healthcare cost of bronchopulmonary dysplasia | https://dx.doi.org/10.1136/bmjopen-2020-045729 | Wrong indication |
| Boonstra, Frouke N. (2022) | The Multidisciplinary Guidelines for Diagnosis and Referral in Cerebral Visual Impairment | https://dx.doi.org/10.3389/fnhum.2022.727565 | Guideline |
| Cristea, A. Ioana (2021) | Outpatient Respiratory Management of Infants, Children, and Adolescents with Post-Prematurity Respiratory Disease: An Official American Thoracic Society Clinical Practice Guideline | https://dx.doi.org/10.1164/rccm.202110-2269ST | Guideline |
| Duijts, Liesbeth (2020) | European Respiratory Society guideline on long-term management of children with bronchopulmonary dysplasia | https://dx.doi.org/10.1183/13993003.00788-2019 | Guideline |
| Gu, Xin (2021) | Evidence summary of human milk fortifier in preterm infants | https://dx.doi.org/10.21037/tp-21-476 | Guideline |
| Luna, Manuel Sanchez (2020) | Expert consensus on palivizumab use for respiratory syncytial virus in developed countries | https://dx.doi.org/10.1016/j.prrv.2018.12.001 | Guideline |
| Mazzola, Catherine A. (2014) | Pediatric hydrocephalus: systematic literature review and evidence-based guidelines. Part 2: Management of posthemorrhagic hydrocephalus in premature infants | https://dx.doi.org/10.3171/2014.7.PEDS14322 | Guideline |
| Morini, Francesco (2022) | Surgical Management of Pediatric Inguinal Hernia: A Systematic Review and Guideline from the European Pediatric Surgeons' Association Evidence and Guideline Committee | https://dx.doi.org/10.1055/s-0040-1721420 | Guideline |
| Nehra, Deepika (2013) | A.S.P.E.N. clinical guidelines: nutrition support of neonatal patients at risk for metabolic bone disease | https://dx.doi.org/10.1177/0148607113487216 | Guideline |
| Ajayi (2023) | Understanding the Domains of Experiences of Black Mothers with Preterm Infants in the United States: A Systematic Literature Review | https://dx.doi.org/10.1007/s40615-022-01425-0 | No guideline with systematic search and/or level of evidence |
| Bacchetta (2022) | Vitamin D and calcium intakes in general pediatric populations: A French expert consensus paper | https://dx.doi.org/10.1016/j.arcped.2022.02.008 | No guideline with systematic search and/or level of evidence |
| Barnes (2022) | Pulmonary haemorrhage in neonates: Systematic review of management | https://dx.doi.org/10.1111/apa.16127 | No guideline with systematic search and/or level of evidence |
| Chen (2018) | Chinese clinical practice guidelines for acute infectious diarrhea in children | https://dx.doi.org/10.1007/s12519-018-0190-2 | No guideline with systematic search and/or level of evidence |
| Evensen (2020) | Long-term motor outcomes of very preterm and/or very low birth weight individuals without cerebral palsy: A review of the current evidence | 10.1016/j.siny.2020.101116 | No guideline with systematic search and/or level of evidence |
| Lechner (2017) | Neurodevelopmental Outcomes of Preterm Infants Fed Human Milk: A Systematic Review | https://dx.doi.org/10.1016/j.clp.2016.11.004 | No guideline with systematic search and/or level of evidence |
| Ong (2015) | Postnatal growth in preterm infants and later health outcomes: a systematic review | https://dx.doi.org/10.1111/apa.13128 | No guideline with systematic search and/or level of evidence |
| Park (2014) | Effects of early intervention on mental or neuromusculoskeletal and movement-related functions in children born low birthweight or preterm: a meta-analysis | https://dx.doi.org/10.5014/ajot.2014.010371 | No guideline with systematic search and/or level of evidence |
| Pfurtscheller (2023) | Insights into Neonatal Cerebral Autoregulation by Blood Pressure Monitoring and Cerebral Tissue Oxygenation: A Qualitative Systematic Review | https://dx.doi.org/10.3390/children10081304 | No guideline with systematic search and/or level of evidence |
| Raiten (2016) | Executive summary: evaluation of the evidence to support practice guidelines for nutritional care of preterm infants-the Pre-B Project | https://dx.doi.org/10.3945/ajcn.115.124222 | No guideline with systematic search and/or level of evidence |
| Raiten (2016) | Working group reports: evaluation of the evidence to support practice guidelines for nutritional care of preterm infants-the Pre-B Project | https://dx.doi.org/10.3945/ajcn.115.117309 | No guideline with systematic search and/or level of evidence |
| Reeves (2022) | A Systematic Review of European Clinical Practice Guidelines for Respiratory Syncytial Virus Prophylaxis | https://dx.doi.org/10.1093/infdis/jiac059 | No guideline with systematic search and/or level of evidence |
| Sisson (2017) | Vaccination timeliness in preterm infants: An integrative review of the literature | https://dx.doi.org/10.1111/jocn.13916 | No guideline with systematic search and/or level of evidence |
| ValdezSandoval (2019) | Intraventricular hemorrhage and posthemorrhagic hydrocephalus in preterm infants: diagnosis, classification, and treatment options | https://dx.doi.org/10.1007/s00381-019-04127-x | No guideline with systematic search and/or level of evidence |
| Wong (2016) | Developmental Assessments in Preterm Children: A Meta-analysis | https://dx.doi.org/10.1542/peds.2016-0251 | No guideline with systematic search and/or level of evidence |
| Yoneda (2021) | Pre-eclampsia Complicated With Maternal Renal Dysfunction Is Associated With Poor Neurological Development at 3 Years Old in Children Born Before 34 Weeks of Gestation | https://dx.doi.org/10.3389/fped.2021.624323 | No guideline with systematic search and/or level of evidence |
| Alshaikh, Belal (2022) | Effect of enteral zinc supplementation on growth and neurodevelopment of preterm infants: a systematic review and meta-analysis | https://dx.doi.org/10.1038/s41372-021-01094-7 | Timepoint not reported |
| Dib, Sarah (2022) | Interventions to Improve Breastfeeding Outcomes in Late Preterm and Early Term Infants | https://dx.doi.org/10.1089/bfm.2022.0118 | Timepoint not reported |
| Koo, Winston (2014) | Human milk and neurodevelopment in children with very low birth weight: a systematic review | https://dx.doi.org/10.1186/1475-2891-13-94 | Timepoint not reported |
| Mahoney, Liam (2020) | Treatment for Post-hemorrhagic Ventricular Dilatation: A Multiple-Treatment Meta-Analysis | https://dx.doi.org/10.3389/fped.2020.00238 | Timepoint not reported |
| Mimouni, Francis B. (2017) | The Use of Multinutrient Human Milk Fortifiers in Preterm Infants: A Systematic Review of Unanswered Questions | https://dx.doi.org/10.1016/j.clp.2016.11.011 | Timepoint not reported |
| Newberry, Sydne J. (2016) | Omega-3 Fatty Acids and Maternal and Child Health: An Updated Systematic Review | https://dx.doi.org/10.23970/AHRQEPCERTA224 | Timepoint not reported |
| Pimpin, Laura (2019) | Effects of animal protein supplementation of mothers, preterm infants, and term infants on growth outcomes in childhood: a systematic review and meta-analysis of randomized trials | https://dx.doi.org/10.1093/ajcn/nqy348 | Timepoint not reported |
| Shulkin, Masha (2018) | n-3 Fatty Acid Supplementation in Mothers, Preterm Infants, and Term Infants and Childhood Psychomotor and Visual Development: A Systematic Review and Meta-Analysis | https://dx.doi.org/10.1093/jn/nxx031 | Timepoint not reported |
| Wang, Qian (2016) | The Effect of Supplementation of Long-Chain Polyunsaturated Fatty Acids During Lactation on Neurodevelopmental Outcomes of Preterm Infant From Infancy to School Age: A Systematic Review and Meta-analysis | https://dx.doi.org/10.1016/j.pediatrneurol.2016.02.017 | Timepoint not reported |
| Zhang, Yu (2023) | Effects of massage therapy on preterm infants and their mothers: a systematic review and meta-analysis of randomized controlled trials | https://dx.doi.org/10.3389/fped.2023.1198730 | Timepoint not reported |
| Abdel-Latif, Mohamed E. (2021) | Surfactant therapy via thin catheter in preterm infants with or at risk of respiratory distress syndrome | https://dx.doi.org/10.1002/14651858.CD011672.pub2 | Wrong timepoint |
| Akbari, Emis (2018) | Kangaroo mother care and infant biopsychosocial outcomes in the first year: A meta-analysis | https://dx.doi.org/10.1016/j.earlhumdev.2018.05.004 | Wrong timepoint |
| Albertella M., Gentyala R. R., Paraskevas T., Ehret D., Bruschettini M. and Soll R. (2023) | Superoxide dismutase for bronchopulmonary dysplasia in preterm infants | https://dx.doi.org/10.1002/14651858.CD013232.pub2 | Wrong timepoint |

| Allen, Elizabeth (2021) | Avoidance of bottles during the establishment of breastfeeds in preterm infants | https://dx.doi.org/10.1002/14651858.CD005252.pub5 | Wrong timepoint |
| --- | --- | --- | --- |
| Collins, Carmel T. (2016) |  | https://dx.doi.org/10.1002/14651858.CD005252.pub3 |  |
| Almadhoob, Abdulraoof (2015) | Sound reduction management in the neonatal intensive care unit for preterm or very low birth weight infants | https://dx.doi.org/10.1002/14651858.CD010333.pub2 | Wrong timepoint |
| Almadhoob, Abdulraoof (2020) |  | https://dx.doi.org/10.1002/14651858.CD010333.pub3 |  |

| Alvarez, Maria Jose (2017) | The effects of massage therapy in hospitalized preterm neonates: A systematic review | https://dx.doi.org/10.1016/j.ijnurstu.2017.02.009 | Wrong timepoint |
| --- | --- | --- | --- |
| Ananthan, Anitha (2020) | Human Milk-Derived Fortifiers Compared with Bovine Milk-Derived Fortifiers in Preterm Infants: A Systematic Review and Meta-Analysis | https://dx.doi.org/10.1093/advances/nmaa039 | Wrong timepoint |
| Askie, Lisa M. (2017) | Effects of targeting lower versus higher arterial oxygen saturations on death or disability in preterm infants | https://dx.doi.org/10.1002/14651858.CD011190.pub2 | Wrong timepoint |
| Athanasopoulou, Eirini (2014) | Effects of kangaroo mother care on maternal mood and interaction patterns between parents and their preterm, low birth weight infants: a systematic review | https://dx.doi.org/10.1002/imhj.21444 | Wrong timepoint |
| Austin, Nicola (2013) | Prophylactic systemic antifungal agents to prevent mortality and morbidity in very low birth weight infants | https://dx.doi.org/10.1002/14651858.CD003850.pub4 | Wrong timepoint |
| Baiad, Abed A. (2023) | A Meta-Analysis of Neurodevelopmental Outcomes following Intravitreal Bevacizumab for the Treatment of Retinopathy of Prematurity | https://dx.doi.org/10.1159/000531541 | Wrong timepoint |
| Balasubramanian, Haribalakrishna (2020) | Umbilical cord milking in preterm infants: a systematic review and meta-analysis | https://dx.doi.org/10.1136/archdischild-2019-318627 | Wrong timepoint |

| Barrington, Keith J. (2010) | Inhaled nitric oxide for respiratory failure in preterm infants | https://dx.doi.org/10.1002/14651858.CD000509.pub4 | Wrong timepoint |
| --- | --- | --- | --- |
| Barrington, Keith J. (2017) |  | https://dx.doi.org/10.1002/14651858.CD000509.pub5 |  |
| Bellu, R. (2010) | Opioids for neonates receiving mechanical ventilation: a systematic review and meta-analysis | https://dx.doi.org/10.1136/adc.2008.150318 | Wrong timepoint |
| Bellu, Roberto (2021) | Opioids for newborn infants receiving mechanical ventilation | https://dx.doi.org/10.1002/14651858.CD013732.pub2 |  |

| Best, Kobi (2018) | Language Exposure of Preterm Infants in the Neonatal Unit: A Systematic Review | https://dx.doi.org/10.1159/000489600 | Wrong timepoint |
| --- | --- | --- | --- |
| Boggini, Tiziana (2021) | Cumulative procedural pain and brain development in very preterm infants: A systematic review of clinical and preclinical studies | https://dx.doi.org/10.1016/j.neubiorev.2020.12.016 | Wrong timepoint |
| Boundy, Ellen O. (2016) | Kangaroo Mother Care and Neonatal Outcomes: A Meta-analysis | https://dx.doi.org/10.1542/peds.2015-2238 | Wrong timepoint |

| Brown, Jennifer V. E. (2016) | Multi-nutrient fortification of human milk for preterm infants | | | https://dx.doi.org/10.1002/14651858.CD000343.pub3 | Wrong timepoint |
| --- | --- | --- | --- | --- | --- |
| Brown, Jennifer Ve (2020) |  | | | https://dx.doi.org/10.1002/14651858.CD000343.pub4 |  |
| Bruschettini, Matteo (2023) | | Caffeine dosing regimens in preterm infants with or at risk for apnea of prematurity | https://dx.doi.org/10.1002/14651858.CD013873.pub2 | | Wrong timepoint |
| Burke, Sara (2018) | | Systematic review of developmental care interventions in the neonatal intensive care unit since 2006 | https://dx.doi.org/10.1177/1367493517753085 | | Wrong timepoint |
| Chan, Stephanie H. T. (2016) | | Nutrition and neurodevelopmental outcomes in preterm infants: a systematic review | https://dx.doi.org/10.1111/apa.13344 | | Wrong timepoint |
| Chawla, Deepak (2010) | | Phenobarbitone for prevention and treatment of unconjugated hyperbilirubinemia in preterm neonates: a systematic review and meta-analysis |  | | Wrong timepoint |
| Choo Y.M., Yip K. X., Fiander M., Ahmad Kamar A., Kamalden T. A., , Tan K. et al. (2025) | | Lutein and zeaxanthin for reducing morbidity and mortality in preterm infants | https://dx.doi.org/10.1002/14651858.CD012178.pub2 | | Wrong timepoint |
| Cleminson, Jemma (2015) | | Prophylactic systemic antifungal agents to prevent mortality and morbidity in very low birth weight infants | https://dx.doi.org/10.1002/14651858.CD003850.pub5 | | Wrong timepoint |
| Cools, Filip (2015) | | Elective high frequency oscillatory ventilation versus conventional ventilation for acute pulmonary dysfunction in preterm infants | https://dx.doi.org/10.1002/14651858.CD000104.pub4 | | Wrong timepoint |
| Darlow, Brian A. (2011) | Vitamin A supplementation to prevent mortality and short- and long-term morbidity in very low birthweight infants | | | https://dx.doi.org/10.1002/14651858.CD000501.pub3 | Wrong timepoint |
| Darlow, Brian A. (2016) |  |  |  | https://dx.doi.org/10.1002/14651858.CD000501.pub4 |  |
| De Nardo, Maria Chiara (2022) | Enteral and parenteral energy intake and neurodevelopment in preterm infants: A systematic review | | | https://dx.doi.org/10.1016/j.nut.2021.111572 | Wrong timepoint |
| de Toledo, Ana Renata Pinto (2023) | What do we know about the sleep effects of caffeine used to treat apnoea of prematurity? A systematic review of the literature | | | https://dx.doi.org/10.1186/s40348-023-00166-2 | Wrong timepoint |
| Delara, Mahin (2019) | Efficacy and safety of pulmonary application of corticosteroids in preterm infants with respiratory distress syndrome: a systematic review and meta-analysis | | | https://dx.doi.org/10.1136/archdischild-2017-314046 | Wrong timepoint |
| Ding, Xiang (2019) | Effects of family-centred care interventions on preterm infants and parents in neonatal intensive care units: A systematic review and meta-analysis of randomised controlled trials | | | https://dx.doi.org/10.1016/j.aucc.2018.10.007 | Wrong timepoint |
| Donohue, Pamela K. (2011) | Inhaled nitric oxide in preterm infants: a systematic review | | | https://dx.doi.org/10.1542/peds.2010-3428 | Wrong timepoint |
| Doyle, Lex W. (2010) | Dexamethasone treatment after the first week of life for bronchopulmonary dysplasia in preterm infants: a systematic review | | | https://dx.doi.org/10.1159/000286212 | Wrong timepoint |
| Doyle, Lex W. (2010) | Dexamethasone treatment in the first week of life for preventing bronchopulmonary dysplasia in preterm infants: a systematic review | | | https://dx.doi.org/10.1159/000286210 | Wrong timepoint |
| Doyle, Lex W. (2017) | Late (> 7 days) systemic postnatal corticosteroids for prevention of bronchopulmonary dysplasia in preterm infants | | | https://dx.doi.org/10.1002/14651858.CD001145.pub4 | Wrong timepoint |
| Doyle, Lex W. (2014) | Late (> 7 days) postnatal corticosteroids for chronic lung disease in preterm infants | | | https://dx.doi.org/10.1002/14651858.CD001145.pub3 |  |
| Doyle, Lex W. (2021) | Early (< 7 days) systemic postnatal corticosteroids for prevention of bronchopulmonary dysplasia in preterm infants | | | https://dx.doi.org/10.1002/14651858.CD001146.pub6 | Wrong timepoint |
| Doyle, Lex W. (2017) | Early (< 8 days) systemic postnatal corticosteroids for prevention of bronchopulmonary dysplasia in preterm infants | | | https://dx.doi.org/10.1002/14651858.CD001146.pub5 |  |
| Doyle, Lex W. (2014) | Early (< 8 days) postnatal corticosteroids for preventing chronic lung disease in preterm infants | | | https://dx.doi.org/10.1002/14651858.CD001146.pub4 |  |

| Embarek-Hernandez, Miriam (2022) | Multisensory stimulation to promote feeding and psychomotor development in preterm infants: A systematic review | https://dx.doi.org/10.1016/j.pedneo.2022.07.001 | Wrong timepoint |
| --- | --- | --- | --- |
| Fabrizio, Veronica (2020) | Individualized versus standard diet fortification for growth and development in preterm infants receiving human milk | https://dx.doi.org/10.1002/14651858.CD013465.pub2 | Wrong timepoint |
| Fenton, Tanis R. (2020) | Higher versus lower protein intake in formula-fed low birth weight infants | https://dx.doi.org/10.1002/14651858.CD003959.pub4 | Wrong timepoint |
| Fischer, Hendrik S. (2017) | Prophylactic Early Erythropoietin for Neuroprotection in Preterm Infants: A Meta-analysis | https://dx.doi.org/10.1542/peds.2016-4317 | Wrong timepoint |
| Flint, Anndrea (2016) | Cup feeding versus other forms of supplemental enteral feeding for newborn infants unable to fully breastfeed | https://dx.doi.org/10.1002/14651858.CD005092.pub3 | Wrong timepoint |
| Fowlie, Peter W. (2010) | Prophylactic intravenous indomethacin for preventing mortality and morbidity in preterm infants | https://dx.doi.org/10.1002/14651858.CD000174.pub2 | Wrong timepoint |
| Gao, Shuqiang (2021) | Systematic review and meta-analysis: the effect of bronchopulmonary dysplasia on neurodevelopment in very low birth weight premature infants | https://dx.doi.org/10.21037/tp-21-449 | Wrong timepoint |
| Guo, Wanying (2023) | Evaluation of the impact of kangaroo mother care on neonatal mortality and hospitalization: A meta-analysis | https://dx.doi.org/10.17219/acem/153417 | Wrong timepoint |
| Haslbeck, Friederike B. (2023) | Musical and vocal interventions to improve neurodevelopmental outcomes for preterm infants | https://dx.doi.org/10.1002/14651858.CD013472.pub2 | Wrong timepoint |
| Hay S., Ovelman C., Zupancic J. A., Doyle L. W., Onland W., Konstantinidis M. et al. (2023) | Systemic corticosteroids for the prevention of bronchopulmonary dysplasia, a network meta-analysis | https://dx.doi.org/10.1002/14651858.CD013730.pub2 | Wrong timepoint |
| Hay, Susanne (2023) | Systemic corticosteroids for the prevention of bronchopulmonary dysplasia, a network meta-analysis | https://dx.doi.org/10.1002/14651858.CD013730.pub2 | Wrong timepoint |
| Henderson-Smart, David J. (2010) | Prophylactic methylxanthine for prevention of apnoea in preterm infants | https://dx.doi.org/10.1002/14651858.CD000432.pub2 | Wrong timepoint |
| Henderson-Smart, David J. (2010) | Prophylactic methylxanthines for endotracheal extubation in preterm infants | https://dx.doi.org/10.1002/14651858.CD000139.pub2 | Wrong timepoint |
| Ho, Jacqueline J. (2015) | Continuous distending pressure for respiratory distress in preterm infants | https://dx.doi.org/10.1002/14651858.CD002271.pub2 | Wrong timepoint |
| Hortensius, Lisa M. (2019) | Postnatal Nutrition to Improve Brain Development in the Preterm Infant: A Systematic Review From Bench to Bedside | https://dx.doi.org/10.3389/fphys.2019.00961 | Wrong timepoint |
| Hunt, Rod (2010) | Ethamsylate for the prevention of morbidity and mortality in preterm or very low birth weight infants | https://dx.doi.org/10.1002/14651858.CD004343.pub2 | Wrong timepoint |
| Ibrahim, Hafis (2011) | Corticosteroids for treating hypotension in preterm infants | https://dx.doi.org/10.1002/14651858.CD003662.pub4 | Wrong timepoint |
| Jackson, Wesley (2018) | Association between furosemide in premature infants and sensorineural hearing loss and nephrocalcinosis: a systematic review | https://dx.doi.org/10.1186/s40748-018-0092-2 | Wrong timepoint |
| Jeevan, Amrit (2022) | Umbilical cord milking versus delayed cord clamping in term and late-preterm infants: a systematic review and meta-analysis | https://dx.doi.org/10.1080/14767058.2021.1884676 | Wrong timepoint |
| Kalteren, Willemien S. (2021) | Anemia and Red Blood Cell Transfusions, Cerebral Oxygenation, Brain Injury and Development, and Neurodevelopmental Outcome in Preterm Infants: A Systematic Review | https://dx.doi.org/10.3389/fped.2021.644462 | Wrong timepoint |
| Kandula, Viswajit (2022) | The role of blood product removal in intraventricular hemorrhage of prematurity: a meta-analysis of the clinical evidence | https://dx.doi.org/10.1007/s00381-021-05400-8 | Wrong timepoint |
| Kaushal, Monika (2021) | Neurodevelopmental outcomes following bevacizumab treatment for retinopathy of prematurity: a systematic review and meta-analysis | https://dx.doi.org/10.1038/s41372-020-00884-9 | Wrong timepoint |
| Khurana, Sonia (2020) | Effect of neonatal therapy on the motor, cognitive, and behavioral development of infants born preterm: a systematic review | https://dx.doi.org/10.1111/dmcn.14485 | Wrong timepoint |
| Kim, Soo-Yeon (2022) | Attachment- and Relationship-Based Interventions during NICU Hospitalization for Families with Preterm/Low-Birth Weight Infants: A Systematic Review of RCT Data | https://dx.doi.org/10.3390/ijerph19031126 | Wrong timepoint |
| King E., Horn D., Gluchowski N., O'Reilly, D., Bruschettini M., Cooper C. et al. (2025) | Safety and efficacy of proton pump inhibitors in preterm infants with gastroesophageal reflux disease | https://dx.doi.org/10.1002/14651858.CD015127.pub2 | Wrong timepoint |
| Kumar, Mohan (2022) | Enteral Vitamin D Supplementation in Preterm or Low Birth Weight Infants: A Systematic Review and Meta-analysis | https://dx.doi.org/10.1542/peds.2022-057092K | Wrong timepoint |
| Lai, Grace Y. (2021) | Timing of Temporizing Neurosurgical Treatment in Relation to Shunting and Neurodevelopmental Outcomes in Posthemorrhagic Ventricular Dilatation of Prematurity: A Meta-analysis | https://dx.doi.org/10.1016/j.jpeds.2021.01.030 | Wrong timepoint |
| Legendre, Valerie (2011) | The evolving practice of developmental care in the neonatal unit: a systematic review | https://dx.doi.org/10.3109/01942638.2011.556697 | Wrong timepoint |
| Liang, Liang (2021) | Sustained low-dose prophylactic early erythropoietin for improvement of neurological outcomes in preterm infants:A systematic review and meta-analysis | https://dx.doi.org/10.1016/j.jad.2021.01.018 | Wrong timepoint |
| Lui, Kei (2018) | Lower versus higher oxygen concentrations titrated to target oxygen saturations during resuscitation of preterm infants at birth | https://dx.doi.org/10.1002/14651858.CD010239.pub2 | Wrong timepoint |
| Ma, Jianglin (2016) | Effects of permissive hypercapnia on pulmonary and neurodevelopmental sequelae in extremely low birth weight infants: a meta-analysis | https://dx.doi.org/10.1186/s40064-016-2437-5 | Wrong timepoint |
| Manja, Veena (2015) | Oxygen saturation target range for extremely preterm infants: a systematic review and meta-analysis | https://dx.doi.org/10.1001/jamapediatrics.2014.3307 | Wrong timepoint |
| Miller, Jacqueline (2018) | A Systematic Review and Meta-Analysis of Human Milk Feeding and Morbidity in Very Low Birth Weight Infants | https://dx.doi.org/10.3390/nu10060707 | Wrong timepoint |
| Mitchell, Kevin (2014) | Arginine supplementation in prevention of necrotizing enterocolitis in the premature infant: an updated systematic review | https://dx.doi.org/10.1186/1471-2431-14-226 | Wrong timepoint |
| Mitra, Souvik (2020) | Early treatment versus expectant management of hemodynamically significant patent ductus arteriosus for preterm infants | https://dx.doi.org/10.1002/14651858.CD013278.pub2 | Wrong timepoint |
| Mitra, Souvik (2022) | Prophylactic cyclo-oxygenase inhibitor drugs for the prevention of morbidity and mortality in preterm infants: a network meta-analysis | https://dx.doi.org/10.1002/14651858.CD013846.pub2 | Wrong timepoint |

| Moe-Byrne, Thirimon (2012) | Glutamine supplementation to prevent morbidity and mortality in preterm infants | https://dx.doi.org/10.1002/14651858.CD001457.pub4 | Wrong timepoint |
| --- | --- | --- | --- |
| Moe-Byrne, Thirimon (2016) |  | https://dx.doi.org/10.1002/14651858.CD001457.pub6 |  |
| Moon, Kwi (2016) | Longchain polyunsaturated fatty acid supplementation in preterm infants | https://dx.doi.org/10.1002/14651858.CD000375.pub5 | Wrong timepoint |
| Schulzke, Sven M. (2011) | Long-chain polyunsaturated fatty acid supplementation in preterm infants | https://dx.doi.org/10.1002/14651858.CD000375.pub4 |  |

| Morag, Iris (2011) | Cycled light in the intensive care unit for preterm and low birth weight infants | https://dx.doi.org/10.1002/14651858.CD006982.pub2 | Wrong timepoint |
| --- | --- | --- | --- |
| More, Kiran (2016) | Endothelin receptor antagonists for persistent pulmonary hypertension in term and late preterm infants | https://dx.doi.org/10.1002/14651858.CD010531.pub2 | Wrong timepoint |
| Moresco, Luca (2023) | Caffeine versus other methylxanthines for the prevention and treatment of apnea in preterm infants | https://dx.doi.org/10.1002/14651858.CD015462.pub2 | Wrong timepoint |
| Morris, Ian Paul (2019) | Efficacy and safety of systemic hydrocortisone for the prevention of bronchopulmonary dysplasia in preterm infants: a systematic review and meta-analysis | https://dx.doi.org/10.1007/s00431-019-03398-5 | Wrong timepoint |
| Nagano, Nobuhiko (2018) | Benefits of umbilical cord milking versus delayed cord clamping on neonatal outcomes in preterm infants: A systematic review and meta-analysis | https://dx.doi.org/10.1371/journal.pone.0201528 | Wrong timepoint |
| Ng G., Bruschettini M., Ibrahim J. and da Silva O. (2024) | Inhaled bronchodilators for the prevention and treatment of chronic lung disease in preterm infants | https://dx.doi.org/10.1002/14651858.CD003214.pub4 | Wrong timepoint |
| Oei, Ju Lee (2022) | Neurodevelopmental outcomes of preterm infants after randomisation to initial resuscitation with lower (FiO2 <0.3) or higher (FiO2 >0.6) initial oxygen levels. An individual patient meta-analysis | https://dx.doi.org/10.1136/archdischild-2021-321565 | Wrong timepoint |
| Ohlsson, Arne (2013) | NIDCAP: a systematic review and meta-analyses of randomized controlled trials | https://dx.doi.org/10.1542/peds.2012-2121 | Wrong timepoint |

| Ohlsson, Arne (2014) | Early erythropoietin for preventing red blood cell transfusion in preterm and/or low birth weight infants | https://dx.doi.org/10.1002/14651858.CD004863.pub4 | Wrong timepoint |
| --- | --- | --- | --- |
| Ohlsson, Arne (2017) | Early erythropoiesis-stimulating agents in preterm or low birth weight infants | https://dx.doi.org/10.1002/14651858.CD004863.pub5 |  |
| Ohlsson, Arne (2020) |  | https://dx.doi.org/10.1002/14651858.CD004863.pub6 |  |

| Ohlsson, Arne (2018) | Paracetamol (acetaminophen) for patent ductus arteriosus in preterm or low birth weight infants | https://dx.doi.org/10.1002/14651858.CD010061.pub3 | Wrong timepoint |
| --- | --- | --- | --- |
| Olaloye, Oluwabunmi (2020) | Role of Nutrition in Prevention of Neonatal Spontaneous Intestinal Perforation and Its Complications: A Systematic Review | https://dx.doi.org/10.3390/nu12051347 | Wrong timepoint |
| Onland W., van de Loo M., Offringa M. and van Kaarm A. (2023) | Systemic corticosteroid regimens for prevention of bronchopulmonary dysplasia in preterm infants | https://dx.doi.org/10.1002/14651858.CD010941.pub3 | Wrong timepoint |
| Onland, Wes (2022) | Late (>= 7 days) inhaled corticosteroids to reduce bronchopulmonary dysplasia in preterm infants | https://dx.doi.org/10.1002/14651858.CD002311.pub5 | Wrong timepoint |

| Onland, Wes (2023) | Systemic corticosteroid regimens for prevention of bronchopulmonary dysplasia in preterm infants | https://dx.doi.org/10.1002/14651858.CD010941.pub3 | Wrong timepoint |
| --- | --- | --- | --- |
| Onland, Wes (2017) |  | https://dx.doi.org/10.1002/14651858.CD010941.pub2 |  |

| Orovou, Eirini (2022) | Correlation between Pacifier Use in Preterm Neonates and Breastfeeding in Infancy: A Systematic Review | https://dx.doi.org/10.3390/children9101585 | Wrong timepoint |
| --- | --- | --- | --- |
| Osborn, David A. (2018) | Higher versus lower amino acid intake in parenteral nutrition for newborn infants | https://dx.doi.org/10.1002/14651858.CD005949.pub2 | Wrong timepoint |
| Ottolini, Katherine M. (2020) | Nutrition and the developing brain: the road to optimizing early neurodevelopment: a systematic review | https://dx.doi.org/10.1038/s41390-019-0508-3 | Wrong timepoint |
| Pammi, Mohan (2020) | Enteral lactoferrin supplementation for prevention of sepsis and necrotizing enterocolitis in preterm infants | https://dx.doi.org/10.1002/14651858.CD007137.pub6 | Wrong timepoint |
| Panchal, Harshad (2023) | Growth and neuro-developmental outcomes of probiotic supplemented preterm infants-a systematic review and meta-analysis | https://dx.doi.org/10.1038/s41430-023-01270-2 | Wrong timepoint |
| Provenzi, Livio (2018) | Do mothers sound good? A systematic review of the effects of maternal voice exposure on preterm infants' development | https://dx.doi.org/10.1016/j.neubiorev.2018.03.009 | Wrong timepoint |
| Qin, Na (2021) | Efficacy and safety of high and low dose recombinant human erythropoietin on neurodevelopment of premature infants: A meta-analysis | https://dx.doi.org/10.1097/MD.0000000000025805 | Wrong timepoint |

| Quigley, Maria (2018) | Formula versus donor breast milk for feeding preterm or low birth weight infants | https://dx.doi.org/10.1002/14651858.CD002971.pub4 | Wrong timepoint |
| --- | --- | --- | --- |
| Quigley, Maria (2019) |  | https://dx.doi.org/10.1002/14651858.CD002971.pub5 |  |

| Qureshi, Mosarrat J. (2013) | D-Penicillamine for preventing retinopathy of prematurity in preterm infants | https://dx.doi.org/10.1002/14651858.CD001073.pub2 | Wrong timepoint |
| --- | --- | --- | --- |

| Rabe, Heike (2012) | Effect of timing of umbilical cord clamping and other strategies to influence placental transfusion at preterm birth on maternal and infant outcomes | https://dx.doi.org/10.1002/14651858.CD003248.pub3 | Wrong timepoint |
| --- | --- | --- | --- |
| Rabe, Heike (2019) |  | https://dx.doi.org/10.1002/14651858.CD003248.pub4 |  |

| Rakshasbhuvankar, Abhijeet A. (2021) | Vitamin A supplementation in very-preterm or very-low-birth-weight infants to prevent morbidity and mortality: a systematic review and meta-analysis of randomized trials | https://dx.doi.org/10.1093/ajcn/nqab294 | Wrong timepoint |
| --- | --- | --- | --- |
| Sankar, Mari Jeeva (2016) | Anti-vascular endothelial growth factor (VEGF) drugs for treatment of retinopathy of prematurity | https://dx.doi.org/10.1002/14651858.CD009734.pub2 | Wrong timepoint |
| Schulzke, Sven M. (2014) | Physical activity programs for promoting bone mineralization and growth in preterm infants | https://dx.doi.org/10.1002/14651858.CD005387.pub3 | Wrong timepoint |
| Shah, Sachin S. (2017) | Inhaled versus systemic corticosteroids for preventing bronchopulmonary dysplasia in ventilated very low birth weight preterm neonates | https://dx.doi.org/10.1002/14651858.CD002058.pub3 | Wrong timepoint |
| Shah, Vibhuti S. (2017) | Early administration of inhaled corticosteroids for preventing chronic lung disease in very low birth weight preterm neonates | https://dx.doi.org/10.1002/14651858.CD001969.pub4 | Wrong timepoint |

| Sharif, Sahar (2020) | Probiotics to prevent necrotising enterocolitis in very preterm or very low birth weight infants | https://dx.doi.org/10.1002/14651858.CD005496.pub5 | Wrong timepoint |
| --- | --- | --- | --- |
| Sharif, Sahar (2023) |  | https://dx.doi.org/10.1002/14651858.CD005496.pub6 |  |

| Sharif, Sahar (2023) | Prebiotics to prevent necrotising enterocolitis in very preterm or very low birth weight infants | https://dx.doi.org/10.1002/14651858.CD015133.pub2 | Wrong timepoint |
| --- | --- | --- | --- |
| Sinha, Bireshwar (2022) | Enteral Zinc Supplementation in Preterm or Low Birth Weight Infants: A Systematic Review and Meta-analysis | https://dx.doi.org/10.1542/peds.2022-057092J | Wrong timepoint |
| Soleimani, Farin (2020) | Do NICU developmental care improve cognitive and motor outcomes for preterm infants? A systematic review and meta-analysis | https://dx.doi.org/10.1186/s12887-020-1953-1 | Wrong timepoint |

| Spittle, Alicia (2015) | Early developmental intervention programmes provided post hospital discharge to prevent motor and cognitive impairment in preterm infants | https://dx.doi.org/10.1002/14651858.CD005495.pub4 | Wrong timepoint |
| --- | --- | --- | --- |
| Spittle, Alicia (2012) |  | https://dx.doi.org/10.1002/14651858.CD005495.pub3 |  |

| Strobel, Natalie A. (2022) | Mother's Own Milk Compared With Formula Milk for Feeding Preterm or Low Birth Weight Infants: Systematic Review and Meta-analysis | https://dx.doi.org/10.1542/peds.2022-057092D | Wrong timepoint |
| --- | --- | --- | --- |
| Subramaniam, Prema (2021) | Prophylactic or very early initiation of continuous positive airway pressure (CPAP) for preterm infants | https://dx.doi.org/10.1002/14651858.CD001243.pub4 | Wrong timepoint |
| Upadhyay, Ravi Prakash (2020) | Effect of prebiotic and probiotic supplementation on neurodevelopment in preterm very low birth weight infants: findings from a meta-analysis | https://dx.doi.org/10.1038/s41390-018-0211-9 | Wrong timepoint |
| Van de Loo M., van Kaarm A., Offringa M., Doyle L. W., Cooper C. and Onland W. (2024) | Corticosteroids for the prevention and treatment of bronchopulmonary dysplasia: an overview of systematic reviews | https://dx.doi.org/10.1002/14651858.CD013271.pub2 | Wrong timepoint |
| van Veenendaal, Nicole R. (2019) | Hospitalising preterm infants in single family rooms versus open bay units: a systematic review and meta-analysis | https://dx.doi.org/10.1016/S2352-4642(18)30375-4 | Wrong timepoint |
| Venkatesh, Vidheya (2012) | The safety and efficacy of red cell transfusions in neonates: a systematic review of randomized controlled trials | https://dx.doi.org/10.1111/j.1365-2141.2012.09180.x | Wrong timepoint |
| Walsh, Verena (2019) | Iodine supplementation for the prevention of mortality and adverse neurodevelopmental outcomes in preterm infants | https://dx.doi.org/10.1002/14651858.CD005253.pub3 | Wrong timepoint |
| Walsh, Verena (2019) | Nutrient-enriched formula versus standard formula for preterm infants | https://dx.doi.org/10.1002/14651858.CD004204.pub3 | Wrong timepoint |
| Wang, Huiping (2015) | A meta-analysis of the protective effect of recombinant human erythropoietin (rhEPO) for neurodevelopment in preterm infants | https://dx.doi.org/10.1007/s12013-014-0265-1 | Wrong timepoint |
| Wang, Peng (2021) | Restrictive versus liberal transfusion thresholds in very low birth weight infants: A systematic review with meta-analysis | https://dx.doi.org/10.1371/journal.pone.0256810 | Wrong timepoint |
| Weisz, Dany E. (2014) | PDA ligation and health outcomes: a meta-analysis | https://dx.doi.org/10.1542/peds.2013-3431 | Wrong timepoint |
| Wolf, H. T. (2012) | Treatment with magnesium sulphate in pre-term birth: a systematic review and meta-analysis of observational studies | https://dx.doi.org/10.3109/01443615.2011.638999 | Wrong timepoint |
| Yang, Wen-Chien (2022) | Fast Feed Advancement for Preterm and Low Birth Weight Infants: A Systematic Review and Meta-analysis | https://dx.doi.org/10.1542/peds.2022-057092G | Wrong timepoint |
| Zeng, Linan (2018) | Corticosteroids for the prevention of bronchopulmonary dysplasia in preterm infants: a network meta-analysis | https://dx.doi.org/10.1136/archdischild-2017-313759 | Wrong timepoint |
| Zhang, Jie (2014) | Neuroprotection with erythropoietin in preterm and/or low birth weight infants | https://dx.doi.org/10.1016/j.jocn.2013.10.040 | Wrong timepoint |
| Zhang, Ruolin (2014) | Effect of dexamethasone on intelligence and hearing in preterm infants: a meta-analysis | https://dx.doi.org/10.4103/1673-5374.130085 | Wrong timepoint |
| Zheng, Yirong (2019) | Long-term effects of the intratracheal administration of corticosteroids for the prevention of bronchopulmonary dysplasia: A meta-analysis | https://dx.doi.org/10.1002/ppul.24452 | Wrong timepoint |
