# Supplementary material for: Identification and Assessment of Systematic Reviews for Evidence‐Based Guideline Recommendations on Follow‐Up of Preterm Born Children: A Mapping Review
Source: Acta Paediatr. 2026 Apr 24;115(7):1384–99. doi: 10.1111/apa.70507 (PMC13250969; doi:10.1111/apa.70507)
Supplement: Supplementary file 5 — Appendix S5: Characteristics of included studies. [file APA-115-1384-s001.docx]

Appendix S5: Characteristics of included studies

| **Author, Year** | **DOI** | **Title** | **Details** | **Methodological Quality** |
| --- | --- | --- | --- | --- |
| Craciunoiu, Oana (2017) | https://dx.doi.org/10.1080/01942638.2016.1185501 | A Systematic Review of the Predictive Validity of Neurobehavioral Assessments During the Preterm Period | Review Design: Prognostic  Framework: PICOTS  P (Population): preterm / very preterm, preterms as full population, "preterm" (<37 weeks)  I / Index / Condition: Neurobehavioral assessments used before term age: General Movements, Test of Infant Motor Performance, Neonatal Behavioral Assessment Scale, Neurobehavioral Assessment of the Preterm Infant, Neonatal Oral Motor Assessment Scale  Comparator / Ref.: Later neurodevelopmental outcome measures such as: Bayley Scales of Infant Development, Alberta Infant Motor Scale, Pediatric Evaluation of Disability Inventory, Neurological diagnoses (e.g., cerebral palsy)  Outcome(s): Neurodevelopmental outcomes including: motor and cognitive development, developmental delay, functional outcomes, neurological impairment  Timing: Prognostic factor timing: 25-38 weeks gestational age (preterm); Outcome timing: 6 months to 10 years corrected age  Setting: NICU during preterm hospitalization | Precheck Critically low |
| Gibbons, James (2023) | 10.1183/23120541.00046-2023 | Increasing airway obstruction through life following bronchopulmonary dysplasia: a meta-analysis | Review Design: Prognostic  Framework: PICOTS  P (Population): Preterm (<37 weeks gestation), including subgroups: <28 weeks (extremely preterm), 28-32 weeks (very preterm), and >32 weeks (moderate-late preterm)  I / Index / Condition: Preterm birth, BPD, Gestational age category, Birth year (proxy for era of neonatal care)  Comparator / Ref.: Term-born individuals (>=37 weeks) primary comparator, Preterm without BPD, Across gestational age bands.   Outcome(s): Lung function measured via: Primary: FEV1/FVC ratio (airway obstruction), Secondary: FEV1, FVC, FEF25-75 (spirometry-based pulmonary measures).  Timing: Prognostic factors measured at birth/neonatal period. Outcomes assessed at cross-sectional time points from childhood to adulthood (up to 53 years).  Setting: Perinatal/neonatal clinical care setting, with follow-up spirometry data collected in research or clinical outpatient settings (childhood, adolescence, adulthood). | Precheck Critically low |
| Heo, Ju S. (2021) | 10.3390/ijerph18062951 | The Long-Term Effect of Preterm Birth on Renal Function: A Meta-Analysis | Review Design: Prognostic  Framework: PICOTS  P (Population): Preterm(>37 weeks), Subgroups: SGA, AGA, very preterm (<32 weeks), extremely preterm (<28 weeks)  I / Index / Condition: Preterm birth (<37 weeks gestation), including SGA and AGA subgroups  Comparator / Ref.: Full-term birth (gestational age >= 37 weeks). This serves as the reference group.   Outcome(s): Long-term renal and cardiovascular outcomes, including: renal function biomarkers (GFR; serum creatinine, BUN, cystatin C, ERPF), Kidney morphology (kidney length, volume, relative volume), Urinary markers (albumin-to-creatinine ratio), Blood pressure (systolic and diastolic, including ambulatory monitoring)  Timing: Prgnostic facotr measured at birth, Putcomes assessd >=2 years postnatal age (range 2-49 years)  Setting: Observational studies conducted in hospital-based or community-based cohorts, often in clinical follow-up or public health monitoring settings. Mostly from high- and middle-income countries. | Precheck Critically low |
| Baker, Stephanie (2023) | https://dx.doi.org/10.1038/s41390-022-02120-w | Machine learning for understanding and predicting neurodevelopmental outcomes in premature infants: a systematic review | Review Design: Diagnostic  Framework: PIRTS  P (Population): preterm <=37 weeks; preterms as full population; "preterm" (<37 weeks)  I / Index / Condition: machine learning techniques applied to various data types, including imaging and connectomes  Comparator / Ref.: standardized neurodevelopmental assessments like the Bayley Scales of Infant Development, Neuro-Sensory Motor Developmental Assessment, and the Mullen Scales of Early Learning  Outcome(s): neurodevelopmental outcomes in preterm infants, specifically predicting cognitive, language, and motor development delays or deficiencies; 12-36 month | Precheck Critically low |
| Beswick, Rachael (2012) | https://dx.doi.org/10.1097/AUD.0b013e31825b1cd9 | Monitoring for postnatal hearing loss using risk factors: a systematic literature review | Review Design: Diagnostic  Framework: PIRTS  P (Population): Children identified with a permanent bilateral or unilateral  hearing loss after they had passed hearing screening/audiologic  assessment at birth; preterms as subgroup; Other: < 38 weeks  I / Index / Condition: targeted surveillance programs using a risk- factor registry to detect postnatal hearing loss  Comparator / Ref.: other programs available that may be more effective than targeted surveillance.  Outcome(s): postnatal hearing loss; 0-12 month; 12-36 month; preschool age (4-6 years) | Precheck Critically low |
| Darsaklis, Vasiliki (2011) | https://dx.doi.org/10.1111/j.1469-8749.2011.04017.x | Predictive validity of Prechtl's Method on the Qualitative Assessment of General Movements: a systematic review of the evidence | Review Design: Diagnostic  Framework: PIRTS  P (Population): high-risk neonates, all diagnoses (e.g. term, preterm, small for gestational age) were considered; different ages at GMsA, preterms as subgroup; "preterm" (<37 weeks); VLBW (<1500gr)  I / Index / Condition: General Movements Assessment (GMA)  Comparator / Ref.: neurodevelopmental outcomes at different ages   Outcome(s): neurodevelopmental outcomes of high-risk neonates; 12-36 month; preschool age (4-6 years); school age (5-12 years); adolescence (13-18) | Precheck Critically low |
| de Albuquerque, Plinio Luna (2015) | https://dx.doi.org/10.3109/17518423.2014.955213 | Accuracy of the Alberta Infant Motor Scale (AIMS) to detect developmental delay of gross motor skills in preterm infants: a systematic review | Review Design: Diagnostic  Framework: PIRTS  P (Population): preterms; preterms as full population; "preterm" (<37 weeks)  I / Index / Condition: Alberta Infant Motor Scale (AIMS)  Comparator / Ref.: Bayley, Griffiths, Peabody  Outcome(s): developmental delay of gross motor skills; 0-3 month; 0-12 month; 12-36 month | Precheck Critically low |
| Fogtmann, Emilie Pi (2017) | https://dx.doi.org/10.1542/peds.2016-1951 | Prognostic Accuracy of Electroencephalograms in Preterm Infants: A Systematic Review | Review Design: Diagnostic  Framework: PIRTS  P (Population): preterm infants; preterms as full population; "preterm" (<37 weeks)  I / Index / Condition: background activity of the EEG, recorded as amplitude-integrated EEG (aEEG) or conventional EEG  Comparator / Ref.: psychomotor developmental tests or neurologic examinations  Outcome(s): neurodevelopmental outcome: developmental delays, cerebral palsy, or death; 0-12 month; 12-36 month; preschool age (4-6 years); school age (5-12 years) | Precheck Critically low |
| George, Joanne M. (2018) | https://dx.doi.org/10.1111/dmcn.13611 | Diagnostic accuracy of early magnetic resonance imaging to determine motor outcomes in infants born preterm: a systematic review and meta-analysis | Review Design: Diagnostic  Framework: PIRTS  P (Population): infants born preterm; preterms as full population; "preterm" (<37 weeks)  I / Index / Condition: early MRI (structural, diffusion, spectroscopic, functional) at <36 weeks PMA  Comparator / Ref.: quantitative motor outcome data from validated tools and/or a confirmed diagnosis of CP at or beyond 12 months corrected age  Outcome(s): adverse motor outcomes and cerebral palsy (CP) in infants born preterm; 12-36 month | Precheck Critically low |
| Guillot, Mireille (2021) | https://dx.doi.org/10.1111/apa.15670 | Comparative performance of head ultrasound and MRI in detecting preterm brain injury and predicting outcomes: A systematic review | Review Design: Diagnostic  Framework: PIRTS  P (Population): Preterm neonates; preterms as full population; "preterm" (<37 weeks)  I / Index / Condition: head ultrasound  Comparator / Ref.: conventional MRI (ie T1- and T2-weighted images)  Outcome(s): detection of preterm brain injury or  the predictive value of neonatal brain imaging for developmental outcomes.; 12-36 month; preschool age (4-6 years); school age (5-12 years) | Precheck Critically low |
| Gunawardana, Shannon (2022) | https://dx.doi.org/10.3390/children9081267 | Pseudorandom Noise Forced Oscillation Technique to Assess Lung Function in Prematurely Born Children | Review Design: Diagnostic  Framework: PIRTS  P (Population): children and young people less than 18 years of age; preterms as full population; "preterm" (<37 weeks)  I / Index / Condition: Pseudorandom Noise Forced Oscillation Technique  Comparator / Ref.: Spirometry,  Intra-Breath Oscillometry,  Interrupter Technique (Rint)  Outcome(s): Lung Function; preschool age (4-6 years); school age (5-12 years); adolescence (13-18) | Precheck Critically low |
| Luttikhuizen dos Santos, Elsa S. (2013) | https://dx.doi.org/10.1016/j.earlhumdev.2013.03.008 | Predictive value of the Bayley scales of infant development on development of very preterm/very low birth weight children: a meta-analysis | Review Design: Diagnostic  Framework: PIRTS  P (Population): children born very preterm and/or with very low birth weight; preterms as full population; <28 weeks; 28 - <32 weeks; VLBW (<1500gr); ELBW (<1000gr)  I / Index / Condition: Bayley Scales of Infant Development (BSID), including BSID-I, BSID-II, and Bayley-IIIâ€‹  Comparator / Ref.: other stan- dardized tests of any aspect of development  Outcome(s): later cognitive, motor, and language functioning in very preterm/VLBW childrenâ€‹; 0-12 month; 12-36 month | Precheck Critically low |
| Noble, Yolande (2012) | https://dx.doi.org/10.1111/j.1469-8749.2010.03903.x | Neonatal assessments for the preterm infant up to 4 months corrected age: a systematic review | Review Design: Diagnostic  Framework: PIRTS  P (Population): preterm infant up to 4 months corrected age; preterms as full population; "preterm" (<37 weeks)  I / Index / Condition: Assessment of Preterm Infants Behaviour (APIB), Neonatal Intensive Care Unit Network Neurobehavioural Scale (NNNS),  Test of Infant Motor Performance (TIMP), Prechtls Assessment of General Movements (GMs), Neurobehavioural Assessment of the Preterm Infant (NAPI), Dubowitz Neurological Assessment of the Preterm and Full-term Infant (Dubowitz), Neuromotor Behavioural Assessment (NMBA), and the  Brazelton Neonatal Behavioural Assessment Scale (NBAS)  primary purposes included prediction (TIMP, GMs, Dubowitz), discrimination (all assessments), and evaluation of change (TIMP, NAPI)  Comparator / Ref.: In the absence of consensus on a criterion standard for neonatal assessment for at-risk preterm infants and with few preterm norms available, all assessments are criterion referenced  Outcome(s): longitudinal neonatal neurobehavioural and neuromotor assessments; Other: up to 4 months corrected age | Precheck Critically low |
| Pires, Camila da Silva (2020) | https://dx.doi.org/10.1590/1984-0462/2020/38/2018286 | PREDICTIVE VALUE OF THE GENERAL MOVEMENTS ASSESSMENT IN PRETERM INFANTS: A META-ANALYSIS | Review Design: Diagnostic  Framework: PIRTS  P (Population): preterm infants; preterms as full population; "preterm" (<37 weeks)  I / Index / Condition: General Movements Assessment (GMA)  (corrected 40-week period up to 20 weeks post-term)  Comparator / Ref.: at final evaluation AIMS: Alberta Infant Motor Scale;  NSMDA: Neurological, Sensory, Motor, Developmental Assessment;  TINE: Touwen Infant Neurological Examination;  MABC-2: Movement Assessment Battery for Children-Second Edition;  DAS-II: Differential Ability Scale-Second Edition;  PDMS-II: Peabody Developmental Motor Scale II;  BSID-II: Bayley Scales of Infant Development II.  Outcome(s): predictive value for minor neurological dysfunctions, cerebral palsy; 12-36 month; preschool age (4-6 years); Other: for final evaluation | Precheck Critically low |
| Caesar, Rebecca (2021) | https://dx.doi.org/10.1111/dmcn.14730 | Clinical tools used in young infants born very preterm to predict motor and cognitive delay (not cerebral palsy): a systematic review | Review Design: Prognostic  Framework: PICOTS  P (Population): Infants born ≤32 weeks gestation and/or ≤1500 g  I / Index / Condition: Early clinical tools (e.g. GMA, HINE, Bayley-III) used ≤6 months  Comparator / Ref.: Standardized developmental tests at 24 months corrected age  Outcome(s): Motor and cognitive delay (non-CP)  Timing: Prognostic factors at ≤6 months → Outcomes at 24 months  Setting: NICUs, hospital follow-up clinics, occasional home/community settings | Precheck Critically low |
| Ahmed, Azza H. (2010) | https://dx.doi.org/10.1111/j.1552-6909.2009.01088.x | Effect of pre- and postdischarge interventions on breastfeeding outcomes and weight gain among premature infants | Review Design: Interventional  Framework: PICO  P (Population): preterm infants; preterms as full population; "preterm" (<37 weeks)  I / Index / Condition: 08b=breastmilk fortification/breast feeding post discharge; parental intervention during hospital stay (breastfeeding, skin-to-skin, bonding etc.); Other: Peer counseling, in-home measurement of milk intake, and early discharge with daily lactation support  Comparator / Ref.: Standard care/placebo  Outcome(s): growth/metabolism; Other: breastfeeding; 0-12 month | AMSTAR Critically low |
| Barlow, Jane (2018) | https://dx.doi.org/10.1002/14651858.CD011754.pub2 | The Neonatal Behavioral Assessment Scale (NBAS) and Newborn Behavioral Observations (NBO) system for supporting caregivers and improving outcomes in caregivers and their infants | Review Design: Interventional  Framework: PICO  P (Population): caregiver-infant (premature or newborn) dyads  in hospitals, clinics, home; preterms as subgroup; not reported  I / Index / Condition: 02c=parent centered/education programs; parent-child-interaction-training/education  Comparator / Ref.: Standard care/placebo  Outcome(s): cognition; motor-development (including CP, DCD); Parent-Child-Interaction; Peer relation/social interaction; Other: Caregiver mental health, knowledge of infant behavior, stress Infant emotional development; not reported | AMSTAR High |
| Benzies, Karen M. (2013) | https://dx.doi.org/10.1186/1471-2393-13-S1-S10 | Key components of early intervention programs for preterm infants and their parents: a systematic review and meta-analysis | Review Design: Interventional  Framework: PICO  P (Population): children born preterm (gestational age less than 37 weeks) and their parents; preterms as full population; "preterm" (<37 weeks)  I / Index / Condition: 02a=parent-child centered; parent-child-interaction-training/education; Other: therapeutic child development support  Comparator / Ref.: Standard care/placebo  Outcome(s): Parent-Child-Interaction; Other: child development (not specified); Other: <1 yr or >1yr | AMSTAR Critically low |
| Bieleninik, Lucja (2016) | https://dx.doi.org/10.1542/peds.2016-0971 | Music Therapy for Preterm Infants and Their Parents: A Meta-analysis | Review Design: Interventional  Framework: PICO  P (Population): Parents/caregivers of/and 24-27weeks GA medically and clinically stable preterm neonates; preterms with all grades of PVL;; preterms as full population; "preterm" (<37 weeks)  I / Index / Condition: 05c=sensory interventions NICU; Other: music therapy  Comparator / Ref.: Standard care/placebo; Other; standard care with placebo (headphones to deliver silence or nonmusic auditory stimuli  Outcome(s): Lung; Other: Breast feeding post discharge; 0-3 month; Other: Long-term effects: One study 29 aimed to  assess infant development, but did  not analyze these data because of  high attrition | AMSTAR Critically low |
| Choo, Candy Sc (2022) | https://dx.doi.org/10.1016/j.jpedsurg.2022.07.001 | Delayed versus early repair of inguinal hernia in preterm infants: A systematic review and meta-analysis | Review Design: Interventional  Framework: PICO  P (Population): preterm infants who were diagnosed with inguinal hernia during the first hospitalisation admission; preterms as full population; "preterm" (<37 weeks)  I / Index / Condition: 10c=hernia; surgery  Comparator / Ref.: Any other "active" intervention; early (before discharge from first hospitalization) versus delayed (after first hospitalization discharge)  Outcome(s): Lung; readmission to hospital; Other: recurrence rate, reoperation, spontaneous resolution; not reported | AMSTAR Low |
| Collins, Carmel T. (2015) | https://dx.doi.org/10.1002/14651858.CD003743.pub2 | Early discharge with home support of gavage feeding for stable preterm infants who have not established full oral feeds | Review Design: Interventional  Framework: PICO  P (Population): preterms requiring no intravenous supplementation at the point of discharge; preterms as full population; "preterm" (<37 weeks)  I / Index / Condition: 02d=home visits; Other: early discharge home with gavage feeds and healthcare support  Comparator / Ref.: Standard care/placebo  Outcome(s): growth/metabolism; infections (RSV, ...); post-discharge death; readmission to hospital; Other: breast feeding; 0-3 month; 0-12 month | AMSTAR Moderate |
| Dell'Aversana, Vincenza (2023) | https://dx.doi.org/10.3390/children10030603 | Emotional Regulation Interventions on Developmental Course for Preterm Children: A Systematic Review of Randomized Control Trials | Review Design: Interventional  Framework: PICO  P (Population): children born prematurely and aged over 3 years; preterms as full population; "preterm" (<37 weeks)  I / Index / Condition: 05b=contingency; rehabilitation; parent-child-interaction-training/education; Other: educational interventions (Group-based physiotherapy intervention, Executive Function training, Mindfulness-based intervention)  Comparator / Ref.: Standard care/placebo; Other: waiting lists and any other therapies or protocols  Outcome(s): behaviour/mental health (include ADHD, autism); Other: emotional and behavioral regulation improvements,  further functional modifications; Other: >3 years,  follow up: 1-12months | AMSTAR Critically low |
| Elfzzani, Zenab (2019) | https://dx.doi.org/10.1002/14651858.CD012240.pub2 | Education of family members to support weaning to solids and nutrition in infants born preterm | Review Design: Interventional  Framework: PICO  P (Population): Parents and families of infants born preterm (at less than 37 weeks of gestation), up to the age of one year of corrected gestational age (CGA).; preterms as full population; "preterm" (<37 weeks)  I / Index / Condition: 08d=Beikost; Other: nutrition education  Comparator / Ref.: Standard care/placebo  Outcome(s): blood pressure/cardiovascular; growth/metabolism; cognition; neurosensory/neurodevelopmental; Parent-Child-Interaction; Quality of Life; post-discharge death; Other: feeding (duration of exclusive breast feeding, adherence to wheaning advice), serum ferritin and haemoglobin levels; Other: 6 months- 5 years | AMSTAR High |
| Evans, Tracey (2014) | https://dx.doi.org/10.1016/j.infbeh.2013.12.009 | Are parenting interventions effective in improving the relationship between mothers and their preterm infants? | Review Design: Interventional  Framework: PICO  P (Population): preterm infants born <37 weeks gestation with no major congenital abnormalities, and the mothers of these infants; preterms as full population; "preterm" (<37 weeks)  I / Index / Condition: 02c=parent centered/education programs; parent-child-interaction-training/education; parental intervention during hospital stay (breastfeeding, skin-to-skin, bonding etc.)  Comparator / Ref.: Standard care/placebo  Outcome(s): Parent-Child-Interaction; 0-3 month; 0-12 month; 12-36 month | AMSTAR Critically low |
| Ferreira, Rachel de Carvalho (2020) | https://dx.doi.org/10.1016/j.jped.2019.05.002 | Effects of early interventions focused on the family in the development of children born preterm and/or at social risk: a meta-analysis | Review Design: Interventional  Framework: PICO  P (Population): premature children with gestational age < 37 weeks, with- out neuromotor abnormalities or congenital abnormalities, and/or children at social risk; preterms as full population; "preterm" (<37 weeks)  I / Index / Condition: 02a=parent-child centered; early intervention programm; parent-child-interaction-training/education  Comparator / Ref.: Standard care/placebo  Outcome(s): cognition; neurosensory/neurodevelopmental; motor-development (including CP, DCD); language development; 0-3 month; 0-12 month; 12-36 month | AMSTAR Critically low |
| Goyal, Neera K. (2013) | https://dx.doi.org/10.1542/peds.2013-0077 | Home visiting and outcomes of preterm infants: a systematic review | Review Design: Interventional  Framework: PICO  P (Population): The targeted preterm infant populations varied in terms of severity of prematurity and LBW status, resulting in ranges in mean gestational age of 30 to 35 weeks, and mean birth weight of 1200 to 2400 g across studies  All of the included programs enrolled infants either during birth hospitalization or soon after discharge.; preterms as full population; "preterm" (<37 weeks)  I / Index / Condition: 02d=home visits; Other: home visiting (nurses, development specialists, trained paraprofessionals or graduate students)  Comparator / Ref.: Standard care/placebo; Any other "active" intervention; also home visiting, but not enhanced or modified like the intervention groups  Outcome(s): growth/metabolism; behaviour/mental health (include ADHD, autism); cognition; neurosensory/neurodevelopmental; motor-development (including CP, DCD); language development; Parent-Child-Interaction; readmission to hospital; 0-3 month; 0-12 month; 12-36 month; preschool age (4-6 years); school age (5-12 years); adolescence (13-18) | AMSTAR Critically low |
| Herd, Michael (2014) | https://dx.doi.org/10.1002/imhj.21480 | Efficacy of preventative parenting interventions for parents of preterm infants on later child behavior: a systematic review and meta-analysis | Review Design: Interventional  Framework: PICO  P (Population): (parents of) infants born premature (<37 weeks); preterms as full population; "preterm" (<37 weeks)  I / Index / Condition: 02c=parent centered/education programs; parent-child-interaction-training/education  Comparator / Ref.: Standard care/placebo  Outcome(s): behaviour/mental health (include ADHD, autism); 12-36 month; preschool age (4-6 years); school age (5-12 years); adolescence (13-18) | AMSTAR Critically low |
| Huang, Pan (2016) | https://dx.doi.org/10.1017/S0007114516001720 | Effects of breast-feeding compared with formula-feeding on preterm infant body composition: a systematic review and meta-analysis | Review Design: Interventional  Framework: PICO  P (Population): preterm infants (â‰¤37 weeks of gestation at birth and/or 2500 g) without congenital malformations or complications affecting body composition; preterms as subgroup; "preterm" (<37 weeks); Other: 2500 g birthweight  I / Index / Condition: 08b=breastmilk fortification/breast feeding post discharge; food supplementation; systemic (oral/iv/im/sc)  Comparator / Ref.: Any other "active" intervention; exclusive or predominant breast-feeding and formula-feeding  Outcome(s): growth/metabolism; 0-3 month; 0-12 month | AMSTAR Low |
| Hughes, Anita J. (2016) | nan | Motor Development Interventions for Preterm Infants: A Systematic Review and Meta-analysis | Review Design: Interventional  Framework: PICO  P (Population): Premature infants born at <37 weeks gestation; preterms as full population; "preterm" (<37 weeks)  I / Index / Condition: 04b=physical therapy; occupational, physical, speech-language therapy; early intervention programm; parent-child-interaction-training/education  Comparator / Ref.: Standard care/placebo  Outcome(s): motor-development (including CP, DCD); Other: at 5 years (3, 6, 12, 24 months) | AMSTAR Critically low |
| Inamdar, Ketaki (2022) | https://dx.doi.org/10.1097/PEP.0000000000000873 | Effect of Contingency Paradigm-Based Interventions on Developmental Outcomes in Young Infants: A Systematic Review | Review Design: Interventional  Framework: PICO  P (Population): Term or preterm infants with a mean age of 12 months and younger and no restriction on medical diagnosis; preterms as subgroup; "preterm" (<37 weeks)  I / Index / Condition: 05a=emotional training; occupational, physical, speech-language therapy  Comparator / Ref.: Standard care/placebo  Outcome(s): neurosensory/neurodevelopmental; motor-development (including CP, DCD); Other: feeding; 0-3 month; 0-12 month | AMSTAR Low |
| Javier, FernÁNdez Rego Francisco (2012) | nan | Efficacy of Early Physiotherapy Intervention in Preterm Infant Motor Development -- A Systematic Review | Review Design: Interventional  Framework: PICO  P (Population): premature infants  of a gestational age inferior to 37 weeks (P) and motor high  risk infants (MHR); preterms as full population; "preterm" (<37 weeks)  I / Index / Condition: 04b=physical therapy; occupational, physical, speech-language therapy; parental intervention during hospital stay (breastfeeding, skin-to-skin, bonding etc.)  Comparator / Ref.: not reported  Outcome(s): motor-development (including CP, DCD); 0-12 month; 12-36 month; preschool age (4-6 years) | AMSTAR Critically low |
| Jones, Lisa J. (2015) | https://dx.doi.org/10.1002/14651858.CD003669.pub2 | Regional (spinal, epidural, caudal) versus general anaesthesia in preterm infants undergoing inguinal herniorrhaphy in early infancy | Review Design: Interventional  Framework: PICO  P (Population): Preterm infants born at less than 37 weeks' gestation, undergoing inguinal herniorrhaphy before 60 weeks' postmenstrual age.; preterms as full population; "preterm" (<37 weeks)  I / Index / Condition: 10c=hernia; Other: anaesthesia; Other: regional (epidural, spinal or caudal) vs. general  Comparator / Ref.: Other: regional vs. general anesthesia; regional vs. general anaesthesia  Outcome(s): recurrence of hernia, Apnoe, incarceration; 12-36 month | AMSTAR Moderate |
| Kermani, Farzaneh (2023) | https://dx.doi.org/10.18502/ijph.v52i8.13402 | Outcome's Classification in Mobile Applications Tailored to Parents of Premature Infants: A Systematic Review | Review Design: Interventional  Framework: PICO  P (Population): fami- lies with preterm newborns and pregnant women  who are at risk of preterm birth; preterms as subgroup; "preterm" (<37 weeks)  I / Index / Condition: 02c=parent centered/education programs; Other: Mobile applications  Comparator / Ref.: Standard care/placebo; Any other "active" intervention; tele-homecare groupsâ€‹  Outcome(s): Parent-Child-Interaction; Other: parental outcomes: maternal stress/stress coping, parenting self-efficacy, satisfaction, anxiety, partnership advocacy/improved parent-infant relationship, feeling of being safe, reassurance and confidence, increase awareness, as well as discharge preparedness,  application outcomes: application usage, ease of use/user-friendly, and usability of the designed application neonatal outcomes: health and clinical items; not reported | AMSTAR Critically low |
| Kumar, Mohan (2022) | https://dx.doi.org/10.1542/peds.2022-057092N | Enteral Multiple Micronutrient Supplementation in Preterm and Low Birth Weight Infants: A Systematic Review and Meta-analysis | Review Design: Interventional  Framework: PICO  P (Population): preterm infants (born before 37 weeks of gestation) and low birth weight (LBW) infants (birth weight less than 2.5 kg); preterms as subgroup; "preterm" (<37 weeks); Other: low birth weight (LBW) infants (birth weight less than 2.5 kg)  I / Index / Condition: 09f=multinutrient; food supplementation; systemic (oral/iv/im/sc)  Comparator / Ref.: Standard care/placebo  Outcome(s): growth/metabolism; cognition; neurosensory/neurodevelopmental; motor-development (including CP, DCD); 0-3 month; 12-36 month | AMSTAR Critically low |
| Kumar, Mohan (2022) | https://dx.doi.org/10.1542/peds.2022-057092K | Enteral Vitamin D Supplementation in Preterm or Low Birth Weight Infants: A Systematic Review and Meta-analysis | Review Design: Interventional  Framework: PICO  P (Population): preterm or LBW infants; preterms as full population; "preterm" (<37 weeks); Other: LBW  I / Index / Condition: 09b=Vit.D; food supplementation; systemic (oral/iv/im/sc)  Comparator / Ref.: Standard care/placebo  Outcome(s): growth/metabolism; neurosensory/neurodevelopmental; post-discharge death; readmission to hospital; Other: -serious morbidity; 0-12 month; 12-36 month | AMSTAR Low |
| Lin, Luling (2019) | https://dx.doi.org/10.1371/journal.pmed.1002952 | Impact of macronutrient supplements for children born preterm or small for gestational age on developmental and metabolic outcomes: A systematic review and meta-analysis | Review Design: Interventional  Framework: PICO  P (Population): infants born preterm or SGA; preterms as full population; "preterm" (<37 weeks); Other: Small for gestational age (SGA)  I / Index / Condition: 08f=early energy/nutrient intake NICU; food supplementation; systemic (oral/iv/im/sc)  Comparator / Ref.: Standard care/placebo  Outcome(s): blood pressure/cardiovascular; growth/metabolism; cognition; neurosensory/neurodevelopmental; motor-development (including CP, DCD); Other: -triglycerides, LDL and HDL concentrations, fasting blood glucose, insulin resistance, and fasting insulin concentrations; 12-36 month; preschool age (4-6 years); school age (5-12 years); adolescence (13-18) | AMSTAR Critically low |
| Lin, Luling (2020) | https://dx.doi.org/10.1371/journal.pmed.1003122 | Impact of macronutrient supplements on later growth of children born preterm or small for gestational age: A systematic review and meta-analysis of randomised and quasirandomised controlled trials | Review Design: Interventional  Framework: PICO  P (Population): infants born preterm or small for gestational age (SGA); preterms as full population; "preterm" (<37 weeks); Other: small for gestational age (SGA)  I / Index / Condition: 08f=early energy/nutrient intake NICU; food supplementation; systemic (oral/iv/im/sc)  Comparator / Ref.: Standard care/placebo  Outcome(s): growth/metabolism; 12-36 month; preschool age (4-6 years); school age (5-12 years); adolescence (13-18) | AMSTAR Low |
| Lin, Luling (2022) | https://dx.doi.org/10.3390/nu14030418 | Sex-Specific Effects of Nutritional Supplements for Infants Born Early or Small: An Individual Participant Data Meta-Analysis (ESSENCE IPD-MA) I-Cognitive Function and Metabolic Risk | Review Design: Interventional  Framework: PICO  P (Population): infants born preterm (before 37 weeks' gestation) or born small for gestational age (SGA, birthweight less than the 10th centile for gestational age); preterms as subgroup; "preterm" (<37 weeks); Other: preterm or SGA  I / Index / Condition: 08f=early energy/nutrient intake NICU; food supplementation; systemic (oral/iv/im/sc)  Comparator / Ref.: Standard care/placebo  Outcome(s): growth/metabolism; cognition; neurosensory/neurodevelopmental; motor-development (including CP, DCD); Quality of Life; post-discharge death; 0-3 month; 0-12 month; 12-36 month; preschool age (4-6 years); school age (5-12 years); adolescence (13-18); Other: adulthood | AMSTAR Critically low |
| Lin, Luling (2022) | https://dx.doi.org/10.3390/nu14020392 | Sex-Specific Effects of Nutritional Supplements for Infants Born Early or Small: An Individual Participant Data Meta-Analysis (ESSENCE IPD-MA) II: Growth | Review Design: Interventional  Framework: PICO  P (Population): infants born preterm (<37 weeks gestation) or born small for gestational age (SGA); preterms as subgroup; "preterm" (<37 weeks); Other: preterm or SGA  I / Index / Condition: 08f=early energy/nutrient intake NICU; food supplementation; systemic (oral/iv/im/sc)  Comparator / Ref.: Standard care/placebo  Outcome(s): growth/metabolism; 0-3 month; 0-12 month; 12-36 month; preschool age (4-6 years); school age (5-12 years); adolescence (13-18); Other: adulthood (>18 years) | AMSTAR Critically low |
| Long, Hui (2012) | https://dx.doi.org/10.1186/1471-2431-12-99 | Benefits of iron supplementation for low birth weight infants: a systematic review | Review Design: Interventional  Framework: PICO  P (Population): infants who were of low birth weight (< 2500 g) or premature (gestational age < 35 weeks); preterms as subgroup; Other: < 2500 g or < 35 week  I / Index / Condition: 11i=Iron; food supplementation; systemic (oral/iv/im/sc)  Comparator / Ref.: Standard care/placebo  Outcome(s): growth/metabolism; neurosensory/neurodevelopmental; Other: Hematologic Parameters, Prevalence of Iron Deficiency and Iron-Deficiency Anemia, Adverse Effects; 0-12 month; 12-36 month; preschool age (4-6 years) | AMSTAR Critically low |
| Manapurath, Rukman M. (2022) | https://dx.doi.org/10.1542/peds.2022-057092I | Enteral Iron Supplementation in Preterm or Low Birth Weight Infants: A Systematic Review and Meta-analysis | Review Design: Interventional  Framework: PICO  P (Population): preterm or low birth weight (LBW) infants who were fed mother's own milk or donor's human milk; preterms as subgroup; 28 - <32 weeks; "preterm" (<37 weeks); Other: LBW  I / Index / Condition: 11i=Iron; food supplementation; systemic (oral/iv/im/sc)  Comparator / Ref.: Standard care/placebo  Outcome(s): blood pressure/cardiovascular; growth/metabolism; behaviour/mental health (include ADHD, autism); cognition; neurosensory/neurodevelopmental; 0-3 month; 0-12 month; 12-36 month; preschool age (4-6 years); school age (5-12 years) | AMSTAR Low |
| Masoudian, Pourya (2019) | https://dx.doi.org/10.1016/j.jpedsurg.2018.11.002 | Optimal timing for inguinal hernia repair in premature infants: a systematic review and meta-analysis | Review Design: Interventional  Framework: PICO  P (Population): preterms with inguinal hernia; preterms as full population; "preterm" (<37 weeks)  I / Index / Condition: 10c=hernia; surgery  Comparator / Ref.: Any other "active" intervention; inguinal hernia repair performed before versus after neonatal intensive care unit (NICU) discharge  Outcome(s): Other: Incarceration, Recurrence, Reoperation; school age (5-12 years); not reported | AMSTAR Low |
| McCarthy, Elaine K. (2019) | https://dx.doi.org/10.1093/nutrit/nuz051 | Iron supplementation in preterm and low-birth-weight infants: a systematic review of intervention studies | Review Design: Interventional  Framework: PICO  P (Population): infants born premature (<37 weeks gestation) or with a birth weight <2500 g; preterms as subgroup; "preterm" (<37 weeks); Other: LBW (<2500gr)  I / Index / Condition: 11i=Iron; 9c=Iron; food supplementation; systemic (oral/iv/im/sc)  Comparator / Ref.: Any other "active" intervention; different regimens of enteral iron supplementation with respect to dose, duration, and timing of initiation  Outcome(s): growth/metabolism; neurosensory/neurodevelopmental; Other: iron status; 0-3 month; 0-12 month; 12-36 month; preschool age (4-6 years); school age (5-12 years) | AMSTAR Critically low |
| Mills, Ryan John (2012) | https://dx.doi.org/10.1002/14651858.CD005095.pub2 | Enteral iron supplementation in preterm and low birth weight infants | Review Design: Interventional  Framework: PICO  P (Population): preterm or LBW infants; preterms as full population; "preterm" (<37 weeks); Other: LBW (<2500 gr)  I / Index / Condition: 11i=Iron; Other: -iron supplementation; systemic (oral/iv/im/sc)  Comparator / Ref.: Standard care/placebo; Any other "active" intervention; 1. Enteral iron supplement of at least 1 mg/kg/day versus no supplementation (i.e. < 1 mg/kg/day). 2. Comparison of different regimens of enteral iron supplementation, in regard to the dose, duration, and timing of initiation of iron supplementation.  Outcome(s): growth/metabolism; neurosensory/neurodevelopmental; Other: blood haemoglobin concentration and mean corpuscular volume (MCV), serum ferritin concentration, transferrin saturation, total iron binding capacity (TIBC); 0-12 month; 12-36 month; preschool age (4-6 years) | AMSTAR Critically low |
| Mohandas, Saranya (2023) | https://dx.doi.org/10.3390/nu15040988 | Effectiveness of Interventions to Manage Difficulties with Breastfeeding for Mothers of Infants under Six Months with Growth Faltering: A Systematic Review Update | Review Design: Interventional  Framework: PICO  P (Population): mothers or caregivers of infants under six months with growth faltering who were experiencing difficulties with breastmilk intake. Infants considered at potential risk of experiencing difficulties were those small or at nutrition-related risk, including those with low birth weight (<2500 g) and those with weight loss or feeding difficulties; preterms as full population; "preterm" (<37 weeks)  I / Index / Condition: 02c=parent centered/education programs; parental intervention during hospital stay (breastfeeding, skin-to-skin, bonding etc.)  Comparator / Ref.: Standard care/placebo  Outcome(s): growth/metabolism; Parent-Child-Interaction; Other: <6 months  (breastfeeding (<6mo) and anthropometric outcomes (<14d, discharge)) | AMSTAR Moderate |
| Moreno-Fernandez, Jorge (2019) | https://dx.doi.org/10.3390/nu11051090 | Iron Deficiency and Iron Homeostasis in Low Birth Weight Preterm Infants: A Systematic Review | Review Design: Interventional  Framework: PICO  P (Population): premature (gestational age <37 weeks) or with a low birth weight (<2500 g); preterms as subgroup; "preterm" (<37 weeks); Other: LBW  I / Index / Condition: 11i=Iron; 09c=Iron; food supplementation; systemic (oral/iv/im/sc)  Comparator / Ref.: Standard care/placebo  Outcome(s): behaviour/mental health (include ADHD, autism); neurosensory/neurodevelopmental; motor-development (including CP, DCD); 0-3 month; 0-12 month; school age (5-12 years) | AMSTAR Critically low |
| Olesen, C. S. (2019) | https://dx.doi.org/10.1007/s10029-019-01877-0 | Risk of incarceration in children with inguinal hernia: a systematic review | Review Design: Interventional  Framework: PICO  P (Population): children with ingui- nal hernias who were treated with no surgery or non-acute  surgery; preterms as subgroup; "preterm" (<37 weeks)  I / Index / Condition: 10c=hernia; surgery  Comparator / Ref.: Any other "active" intervention; different waiting times until surgery was performed  Outcome(s): Other: incarceration rate, Recurrence rate of inguinal hernia, Testicular atrophy; Other: at 10 years | AMSTAR Low |
| Patronick, Jamie (2023) | https://dx.doi.org/10.1093/jpepsy/jsad031 | Parenting Interventions Targeting Behavior for Children Born Preterm or Low Birth Weight: A Systematic Review | Review Design: Interventional  Framework: PICO  P (Population): children born preterm and/or with LBW and their caregivers; preterms as subgroup; "preterm" (<37 weeks); Other: LBW  I / Index / Condition: 02c=parent centered/education programs; early intervention programm; parent-child-interaction-training/education  Comparator / Ref.: Any other "active" intervention; other developmental intervention groups  Outcome(s): behaviour/mental health (include ADHD, autism); Parent-Child-Interaction; Other: after discharge | AMSTAR High |
| Pogorelic, Zenon (2021) | https://dx.doi.org/10.3390/children8100853 | Comparison of Recurrence and Complication Rates Following Laparoscopic Inguinal Hernia Repair among Preterm versus Full-Term Newborns: A Systematic Review and Meta-Analysis | Review Design: Interventional  Framework: PICO  P (Population): preterm newborns with unilateral or bilateral inguinal hernia undergoing laparoscopic inguinal hernia repair (LHR); preterms as full population; "preterm" (<37 weeks)  I / Index / Condition: 10c=hernia; surgery  Comparator / Ref.: Other: full-term newborns undergoing laparoscopic inguinal hernia repair  Outcome(s): Other: recurrence of hernia and the proportion of children developing postoperative complications; not reported | AMSTAR Critically low |
| Santos, Carlos (2023) | https://dx.doi.org/10.3390/ijerph20085610 | Effects of Exposure to Formal Aquatic Activities on Babies Younger Than 36 Months: A Systematic Review | Review Design: Interventional  Framework: PICO  P (Population): 0 to 36 months of age infants; not reported; not reported  I / Index / Condition: 07a=sports; Other: formal aquatic activities, specifically baby swimming programs and aquatic therapy programs for infants  Comparator / Ref.: Any other "active" intervention; Other: same condition of aquatic exposure with the control or/and before and after exposure; Table 5: home-based early intervention only, no intervention besides the daycare center participation  Outcome(s): growth/metabolism; infections (RSV, ...); neurosensory/neurodevelopmental; motor-development (including CP, DCD); Other: aquatic therapy: newborns: clinical parameters during hospitalization preterm infants: pain, sleep cycle and wakefulness, physiological parameters (timepoint of outcome during hospitalization???); 0-3 month; 0-12 month; 12-36 month | AMSTAR Critically low |
| Spiegler, Juliane (2019) | https://dx.doi.org/10.1111/apa.14726 | Association of physical activity and cardiorespiratory function or BMI and body composition in preterm-born individuals: a systematic review | Review Design: Interventional  Framework: PICO  P (Population): children (age 2 to17) or adults (age 18 and above) born at a gestational age of less than 37 weeks; preterms as full population; "preterm" (<37 weeks)  I / Index / Condition: Other: physical activity  Comparator / Ref.: Other: no or less physical activity  Outcome(s): Other: BMI, body composition, cardiorespiratory fitness; 12-36 month; preschool age (4-6 years); school age (5-12 years); adolescence (13-18); Other: above 18 years old | AMSTAR Critically low |
| Vissers, Karin M. (2018) | https://dx.doi.org/10.1159/000488732 | The Timing of Initiating Complementary Feeding in Preterm Infants and Its Effect on Overweight: A Systematic Review | Review Design: Interventional  Framework: PICO  P (Population): preterm infants; preterms as full population; "preterm" (<37 weeks)  I / Index / Condition: 08d=Beikost; Other: initiating complementary feeding  Comparator / Ref.: Other: different time points of starting complementary feeding  Outcome(s): growth/metabolism; 0-3 month; 0-12 month; 12-36 month | AMSTAR Critically low |
| Yang, Wen-Chien (2022) | https://dx.doi.org/10.1542/peds.2022-057092H | Duration of Exclusive Breastfeeding for Preterm or Low Birth Weight Infants: A Systematic Review and Meta-analysis | Review Design: Interventional  Framework: PICO  P (Population): preterm infants <37weeks or LBW <2500g; preterms as subgroup; "preterm" (<37 weeks); Other: LBW infants  I / Index / Condition: 02c=parent centered/education programs; parental intervention during hospital stay (breastfeeding, skin-to-skin, bonding etc.); Other: exclusive breastfeeding for less than 6 months  Comparator / Ref.: Any other "active" intervention; duration of exclusive breastfeeding for less than 6 months compared with 6 months  Outcome(s): growth/metabolism; infections (RSV, ...); behaviour/mental health (include ADHD, autism); cognition; neurosensory/neurodevelopmental; motor-development (including CP, DCD); language development; post-discharge death; readmission to hospital; Other: allergic diseases; 0-12 month; 12-36 month | AMSTAR Low |
| Young, Lauren (2013) | https://dx.doi.org/10.1002/14651858.CD004866.pub4 | Multinutrient fortification of human breast milk for preterm infants following hospital discharge | Review Design: Interventional  Framework: PICO  P (Population): Preterm infants (< 37 weeks' gestation at birth) and low birth weight infants (< 2.5 kg) receiving human breast milk following discharge from hospital.; preterms as full population; "preterm" (<37 weeks); Other: LBW  I / Index / Condition: 09f=multinutrient; food supplementation; Other: Supplementation of human breast milk with more than one nutrient; systemic (oral/iv/im/sc)  Comparator / Ref.: Standard care/placebo  Outcome(s): blood pressure/cardiovascular; growth/metabolism; cognition; neurosensory/neurodevelopmental; Education; Other: Duration of breast milk-feeding  bone mineralisation, bone mineral content, rickets  blood pressure, rickets on long-term follow-up not reported; 0-12 month; not reported; Other: 3-4months,12 months Neurodevelopmental outcomes at >12 months corrected age Cognitive and educational outcomes at > five years | AMSTAR Low |
| McCormick, Felicia M. (2010) | https://dx.doi.org/10.1002/14651858.CD004866.pub3 | Multinutrient fortification of human breast milk for preterm infants following hospital discharge |  |  |
| Young, Lauren (2016) | https://dx.doi.org/10.1002/14651858.CD004696.pub5 | Nutrient-enriched formula versus standard formula for preterm infants following hospital discharge | Review Design: Interventional  Framework: PICO  P (Population): We included preterm infants fed formula (exclusively or as a supplement to human breast milk) after discharge from hospital. The intervention may have commenced up to one week before planned discharge from hospital; preterms as full population; "preterm" (<37 weeks)  I / Index / Condition: 09a=nutrient encriched post-discharge formula; food supplementation; systemic (oral/iv/im/sc)  Comparator / Ref.: Any other "active" intervention; Postdischarge formula: energy content > 72 kcal/100 mL (but ≤ 75 kcal/100 mL) and protein content > 1.7 g /100 mL; or Preterm formula: energy content > 75 kcal/100 mL and protein content > 2.0 g/100 mL.  Outcome(s): blood pressure/cardiovascular; growth/metabolism; ear-nose-throat; eyes; cognition; neurosensory/neurodevelopmental; motor-development (including CP, DCD); Other: -feed intolerance  -measures of bone mineralization -blood pressure -BMI; 0-12 month; 12-36 month; school age (5-12 years) | AMSTAR High |
| Young, Lauren (2012) | https://dx.doi.org/10.1002/14651858.CD004696.pub4 | Nutrient-enriched formula versus standard term formula for preterm infants following hospital discharge |  |  |
| Zhang, Xin (2021) | https://dx.doi.org/10.1097/JPN.0000000000000065 | Early Intervention for Preterm Infants and Their Mothers: A Systematic Review | Review Design: Interventional  Framework: PICO  P (Population): mothers and their infants (before 37- week gestation and/or low birth weight <2500 g); preterms as subgroup; "preterm" (<37 weeks); Other: low birth weight <2500 g  I / Index / Condition: 02a=parent-child centered; parent-child-interaction-training/education; Other: infant developmental support  Comparator / Ref.: Standard care/placebo  Outcome(s): behaviour/mental health (include ADHD, autism); language development; Parent-Child-Interaction; 0-12 month; 12-36 month | AMSTAR Critically low |
| Agrawal, Sachin (2018) | https://dx.doi.org/10.1542/peds.2018-0134 | Prevalence of Autism Spectrum Disorder in Preterm Infants: A Meta-analysis | Review Design: Prevalence/incidence  Framework: CoCoPop  P (Population): ; children born preterm and with different gestational age  Context (CoCoPop): not reported | Precheck Critically low |
| Chen, Yi (2022) | https://dx.doi.org/10.1007/s00383-021-05054-2 | The prevalence and clinical presentation of Hirschsprung's disease in preterm infants: a systematic review and meta-analysis | Review Design: Prevalence/incidence  Framework: CoCoPop  P (Population): ; children diagnosed with HD -> incidence and prevalence of preterm infants  Context (CoCoPop): Therefore, the current quantitative study was performed to determine the prevalence and clinical presentation of HD in preterm infants. | Precheck Critically low |
| Fitzallen, Grace C. (2021) | https://dx.doi.org/10.1097/DBP.0000000000000898 | Anxiety and Depressive Disorders in Children Born Preterm: A Meta-Analysis | Review Design: Prevalence/incidence  Framework: CoCoPop  P (Population): ; children between 3 and 19 years of age born preterm  Context (CoCoPop): Preterm birth is associated with a high prevalence of psychiatric disorders including internalizing problems, particularly anxiety and depression the higher prevalence of anxiety found in this high-risk population is likely to have considerable impacts on educational, social, and occupational functioning | Precheck Critically low |
| Mauskopf, Josephine (2016) | https://dx.doi.org/10.1097/INF.0000000000001163 | Respiratory Syncytial Virus Hospitalizations in Healthy Preterm Infants: Systematic Review | Review Design: Prevalence/incidence  Framework: CoCoPop  P (Population): ; preterm infants born between 29 and 35 weeks of gestational age (WGA) who do not have chronic lung disease or other major coexisting conditions and who did not receive RSV immunoprophylaxisâ€‹  Context (CoCoPop): Healthy preterm infants not given RSV immunoprophylaxis | Precheck Critically low |
| Milner, K. M. (2015) | https://dx.doi.org/10.1179/2046905515Y.0000000043 | Long-term neurodevelopmental outcome in high-risk newborns in resource-limited settings: a systematic review of the literature | Review Design: Prevalence/incidence  Framework: CoCoPop  P (Population): ; infants who were premature (v37 weeks gestation), of low birthweight (v2500 g), very low birthweight (v1500 g) or extremely low birthweight (v1000g) or who experienced intrapartum-related events (at birth asphyxia) or serious infections (i.e. sepsis, pneumonia or meningitis)  Context (CoCoPop): low- and middle income countries | Precheck Critically low |
| Niutanen, Ulla (2020) | https://dx.doi.org/10.1111/apa.14953 | Systematic review of sensory processing in preterm children reveals abnormal sensory modulation, somatosensory processing and sensory-based motor processing | Review Design: Prevalence/incidence  Framework: CoCoPop  P (Population): ; preterm-born children born before 37 weeks of gestation  Context (CoCoPop): The focus was broadly on preterm-born children regardless of their specific environmental conditions. The studies revealed wide variation of atypical sensory processing: 28%-87% in sensory modulation, 9%-70% in somatosensory processing and 20%-70% in sensory-based motor processing | Precheck Critically low |
| Oskoui, Maryam (2013) | https://dx.doi.org/10.1111/dmcn.12080 | An update on the prevalence of cerebral palsy: a systematic review and meta-analysis | Review Design: Prevalence/incidence  Framework: CoCoPop  P (Population): ; (1) the overall prevalence of CP in children at certain ages and in all live births;  (2) the prevalence of CP in relation to four birthweight categories for live births (<1000g, 1000-1499g, 1500-2499g, and >2500g) and using three birthweight categories for neonatal survivors (<1500g, 1500-2499g, and >2500g); and  (3) the prevalence of CP in extremely preterm newborn infants in relation to five gestational weeks (23wks, 24wks, 25wks, 26wks, and 27wks) and the prevalence of CP in relation to four gestational age categories (<28wks, 28-31wks, 32-36wks, and >36wks).  Context (CoCoPop): constant overall estimate per live births in recent years despite the addition of new data and increased survival of at-risk preterm infants.  Although the prevalence of disease isunchanged, future studies are needed to evaluate a change over time in the phenotypic spectrum of CP.  Studies on the prevalence of CP in adolescence and adulthood would also be important future goals as the majority of children with CP are expected to survive into adulthood. | Precheck Critically low |
| Pados, Britt Frisk (2021) | https://dx.doi.org/10.1186/s12887-021-02574-7 | Prevalence of problematic feeding in young children born prematurely: a meta-analysis | Review Design: Prevalence/incidence  Framework: CoCoPop  P (Population): ; children under 4 years of age who were born prematurely (< 37 weeks gestation)  Context (CoCoPop): Problematic feeding occurs in approximately 42% of children under 4 years of age who were born prematurely (< 37 weeks gestation). To date, the study of problematic feeding in children has been limited by a lack of definition of the problem and lack of valid and reliable measures.  improved definition of the problem, along with newly-developed, psychometrically-sound measures of feeding can be used to improve upon the research and care of problematic feeding in children born preterm and with other medical conditions.  A large epidemiological study using a comprehensive and psychometrically-sound assessment of feeding is needed to determine the true prevalence of problematic feeding in children born preterm | Precheck Critically low |
| Petrou, Stavros (2019) | https://dx.doi.org/10.1136/archdischild-2018-315778 | Economic consequences of preterm birth: a systematic review of the recent literature (2009-2017) | Review Design: Prevalence/incidence  Framework: CoCoPop  P (Population): ; The population included in the study comprises individuals born preterm (<37 weeks gestation) or with low birth weight (<2500 g). The study specifically focuses on the economic consequences of preterm birth, considering various sectors such as health services, other sectors of the economy, families, carers, and society at large.  Context (CoCoPop): The context of the study includes developed countries. | Precheck Critically low |
| Allotey, J. (2018) | https://dx.doi.org/10.1111/1471-0528.14832 | Cognitive, motor, behavioural and academic performances of children born preterm: a meta-analysis and systematic review involving 64 061 children | Review Design: Prognostic  Framework: PICOTS  P (Population): children born with different degrees of prematurity; preterms as full population; <28 weeks; 28 - <32 weeks; 32 - <37 weeks  I / Index / Condition: Gestational age  Comparator / Ref.: comparison with full term  Outcome(s): behaviour/mental health (include ADHD, autism); cognition; motor-development (including CP, DCD); Education; 12-36 month; preschool age (4-6 years); school age (5-12 years); adolescence (13-18)  Timing: birth; pre-school age, primary and secondary school ages  Setting: association between preterm birth and neurodevelopmental outcomes -> additional academic, emotional and behavioural needs | Precheck Critically low |
| Alshaikh, B. (2013) | https://dx.doi.org/10.1038/jp.2012.167 | Neurodevelopmental outcomes of very low birth weight infants with neonatal sepsis: systematic review and meta-analysis | Review Design: Prognostic  Framework: PICOTS  P (Population): VLBW infants exposed to culture-proven sepsis in the neonatal period; preterms as full population; <28 weeks; 28 - <32 weeks; VLBW (<1500gr); ELBW (<1000gr)  I / Index / Condition: neonatal complication (BPD, IVH, PVL, NEC, Sepsis)  Comparator / Ref.: Any other prognostic factor/model; similar infants without sepsis  Outcome(s): neurosensory/neurodevelopmental; motor-development (including CP, DCD); 0-12 month; 12-36 month; preschool age (4-6 years); school age (5-12 years)  Timing: neonatal period; childhood  Setting: VLBW infants surviving sepsis in the neonatal period are at higher risk for neurodevelopmental disability including CP. need for continued long-term follow-up of these infants | Precheck Critically low |
| Amin, Sanjiv B. (2011) | https://dx.doi.org/10.1007/s10803-010-1169-6 | Is neonatal jaundice associated with Autism Spectrum Disorders: a systematic review | Review Design: Prognostic  Framework: PICOTS  P (Population): term and preterm infants; preterms as subgroup; "preterm" (<37 weeks)  I / Index / Condition: Gestational age; Other: neonatal jaundice  Outcome(s): behaviour/mental health (include ADHD, autism); not reported  Timing: neonatal period; not reported  Setting: Is there an association between unconjungated hyperbilirubinemia and ASD?  If neonatal jaundice predisposes infants to ASD, neonatal interventions  aimed at preventing and correcting jaundice could be a promising strategy for reducing  jaundice associated ASD and ASD related expenditures in the future. | Precheck Critically low |
| Andraweera, Prabha H. (2021) | https://dx.doi.org/10.1017/S2040174420000914 | Cardiovascular risk factors in those born preterm - systematic review and meta-analysis | Review Design: Prognostic  Framework: PICOTS  P (Population): preterm born; preterms as full population; "preterm" (<37 weeks)  I / Index / Condition: Gestational age  Comparator / Ref.: comparison with full term  Outcome(s): blood pressure/cardiovascular; growth/metabolism; 0-3 month; 0-12 month; 12-36 month; preschool age (4-6 years); school age (5-12 years); adolescence (13-18)  Timing: birth; were predicted over varying lengths of time, depending on the individual study.  Setting: The intended setting and role of the prognostic factors was to assess the influence of gestational age on cardiovascular risk factors in offspring. The study aimed to evaluate the impact of prematurity on cardiovascular health in later life. | Precheck Critically low |
| Ang, Ju Li (2023) | https://dx.doi.org/10.1136/archdischild-2022-324157 | Mortality and neurodevelopmental outcomes of infants with spontaneous intestinal perforation: a systematic review and meta-analysis | Review Design: Prognostic  Framework: PICOTS  P (Population): very preterm infants (<32 weeks) with spontaneous intestinal perforation (SIP); preterms as full population; 28 - <32 weeks  I / Index / Condition: neonatal complication (BPD, IVH, PVL, NEC, Sepsis); Other: spontaneous intestinal perforation  Comparator / Ref.: Any other prognostic factor/model; no SIP or necrotising enterocolitis (NEC)  Outcome(s): ear-nose-throat; eyes; neurosensory/neurodevelopmental; motor-development (including CP, DCD); post-discharge death; Other: > 1 year...  Timing: before discharge; >1 year  Setting: SIP in very preterm infants is associated  with higher odds of mortality, severe disability, and  death or disability | Precheck Critically low |
| Arpi, Elena (2019) | https://dx.doi.org/10.1111/apa.14836 | Worse global intellectual and worse neuropsychological functioning in preterm-born children at preschool age: a meta-analysis | Review Design: Prognostic  Framework: PICOTS  P (Population): three to five years old children born at <32 weeks gestational age or < 1500 g birth weight; preterms as full population; <28 weeks; 28 - <32 weeks; VLBW (<1500gr); ELBW (<1000gr)  I / Index / Condition: Gestational age  Comparator / Ref.: comparison with full term; Any other prognostic factor/model; comparison EPT/ ELBW and VPT/ VLBW  Outcome(s): cognition; preschool age (4-6 years)  Timing: birth; preschool age  Setting: The purpose of this meta analysis was to evaluate the long-term effects of preterm birth on early childhood cognitive development and function emerging in preschool age, while most follow-up programs last until age 2 years and would miss out on these problems. | Precheck Critically low |
| Backes, Carl H. (2021) | https://dx.doi.org/10.1016/j.ajog.2020.07.051 | Proactive neonatal treatment at 22 weeks of gestation: a systematic review and meta-analysis | Review Design: Prognostic  Framework: PICOTS  P (Population): infants who were born at 22 weeks (22 0/7 to 22 6/7 weeks)of gestation and provided proactive treatment; preterms as full population; Other: <22 weeks  I / Index / Condition: Gestational age; Other: proactive neonatal treatment (endotracheal intubation, face mask ventilation, nasal continuous positive airway pressure, chest compressions, or epinephrine administration)  Outcome(s): neurosensory/neurodevelopmental; Other: survival,  survival without major morbidity (using definition #1 that included any BPD),  survival without moderate or severe NDI.; not reported  Timing: birth; not reported  Setting: Although survival is an objective endpoint and remains of greatimportance to families with threatened preterm birth at 22 weeks of gestation, the paucity of data on longer-term sequelae, including quality of life, socialization, and school achievement, hinders the practice of evidence-based shared decision making with families. survival without moderate or severe disability is not equivalent to survival without impairment, wherein even those with no impairment on BSID assessments remain at risk for poor longer-term outcomes at school age. | Precheck Critically low |
| Been, Jasper V. (2014) | https://dx.doi.org/10.1371/journal.pmed.1001596 | Preterm birth and childhood wheezing disorders: a systematic review and meta-analysis | Review Design: Prognostic  Framework: PICOTS  P (Population): children (0,5-18 years) born preterm; preterms as full population; 28 - <32 weeks; 32 - <37 weeks; "preterm" (<37 weeks)  I / Index / Condition: Gestational age  Comparator / Ref.: comparison with full term  Outcome(s): Lung; 0-12 month; 12-36 month; preschool age (4-6 years); school age (5-12 years); adolescence (13-18)  Timing: birth; 0,5 to 18 years  Setting: There is compelling evidence that preterm birth, particularly very preterm birth, increases the risk of asthma. | Precheck Critically low |
| Bensi, Caterina (2020) | https://dx.doi.org/10.1111/ipd.12646 | Relationship between preterm birth and developmental defects of enamel: A systematic review and meta-analysis | Review Design: Prognostic  Framework: PICOTS  P (Population): preterm children; preterms as full population; "preterm" (<37 weeks)  I / Index / Condition: Gestational age  Comparator / Ref.: comparison with full term  Outcome(s): teeth; 0-3 month; 0-12 month; 12-36 month; preschool age (4-6 years); school age (5-12 years); adolescence (13-18)  Timing: birth; early childhood to adulthood  Setting: The results of this meta-analysis showed a three times increased risk  of developing DDE in preterm children. | Precheck Critically low |
| Blair, Lisa M. (2016) | https://dx.doi.org/10.1177/1099800415605379 | Integrative Review of Genetic Factors Influencing Neurodevelopmental Outcomes in Preterm Infants | Review Design: Prognostic  Framework: PICOTS  P (Population): preterm infants who do not have a rare, genetic disease; preterms as full population; "preterm" (<37 weeks)  I / Index / Condition: Other: genetic factors potentially influencing neurodevelopmental outcomes in preterm infants  Outcome(s): behaviour/mental health (include ADHD, autism); cognition; neurosensory/neurodevelopmental; motor-development (including CP, DCD); not reported  Timing: birth; not reported  Setting: Numerous genes (n = 43) and additional large deletion copy number variants were associated with neurodevelopmental outcomes, including cognition, attention, perception, psychiatric disease, autism spectrum disorder, cerebral palsy, infant behavior, and alterations in brain architecture. [...] several avenues of investigation offer promise, including large (>100 kb) copy number variants and the candidate genes MET, NRG3, and SLC6A4, each of which were reported to have associations with neurodevelopmental outcomes in multiple, high-quality studies. | Precheck Critically low |
| Boonzaaijer, Marike (2021) | https://dx.doi.org/10.1111/cch.12830 | Factors associated with gross motor development from birth to independent walking: A systematic review of longitudinal research | Review Design: Prognostic  Framework: PICOTS  P (Population): healthy PT or full-term (FT) infants; preterms as subgroup; "preterm" (<37 weeks)  I / Index / Condition: Gestational age  Comparator / Ref.: comparison with full term  Outcome(s): motor-development (including CP, DCD); 0-12 month  Timing: Birth; 0-60 months  Setting: GA: Four studies with high RoB moderate evidence that a shorter GA for infants is negatively associated with GMD in the age range 0-18 months because GA and BW were highly correlated, it is not clear whether these differences are primarily due to GA or BW  Birthweight: evidence that low birthweight (LBW) (<2,500 g) in both PT and FT infants is associated with a more delayed GMD in the age range 4 to24 months. All studies that included PT infants accounted their outcomes to GA  In a mixed population of infants (GA 27-46.5 weeks), Flensborg-Madsen and Mortensen (2017) showed that BW in addition to GA explained most of the variance in motor milestone attainment. | Precheck Critically low |
| Bosanquet, Margot (2013) | https://dx.doi.org/10.1111/dmcn.12140 | A systematic review of tests to predict cerebral palsy in young children | Review Design: Prognostic  Framework: PICOTS  P (Population): infants at risk of cerebral palsy; preterms as subgroup; "preterm" (<37 weeks)  I / Index / Condition: Gestational age  Outcome(s): motor-development (including CP, DCD); 0-3 month; 0-12 month; 12-36 month; preschool age (4-6 years); school age (5-12 years)  Timing: infancy and early childhood; childhood  Setting: The intended setting was largely clinical, involving high-risk populations in hospital settings. The role of the prognostic factors was to aid in the early diagnosis and management of cerebral palsy in young children. | Precheck Critically low |
| Broring, Tinka (2017) | https://dx.doi.org/10.1371/journal.pone.0170828 | Sensory modulation in preterm children: Theoretical perspective and systematic review | Review Design: Prognostic  Framework: PICOTS  P (Population): preterm infants and children (<37 weeks of gestation); preterms as full population; "preterm" (<37 weeks)  I / Index / Condition: Gestational age  Comparator / Ref.: comparison with full term  Outcome(s): behaviour/mental health (include ADHD, autism); cognition; neurosensory/neurodevelopmental; Other: Temperament; 0-12 month; 12-36 month; preschool age (4-6 years); school age (5-12 years); adolescence (13-18)  Timing: birth; childhood and adolescence  Setting: Sensory modulation problems may play a key role in understanding neurocognitive and behavioral sequelae in preterm children.  Some support is found for a dose-responserelationship between both white matter brain injury and length of NICU stay and sensory modulation problems. | Precheck Critically low |
| Brossard-Racine, Marie (2015) | https://dx.doi.org/10.1007/s12311-014-0597-9 | Developmental cerebellar cognitive affective syndrome in ex-preterm survivors following cerebellar injury | Review Design: Prognostic  Framework: PICOTS  P (Population): preterms with direct Cerebellar injury; preterms as full population; "preterm" (<37 weeks)  I / Index / Condition: neonatal complication (BPD, IVH, PVL, NEC, Sepsis)  Comparator / Ref.: comparison with full term; Any other prognostic factor/model; ex-preterm infants with a normal structural cerebrum and cerebellum  Outcome(s): behaviour/mental health (include ADHD, autism); cognition; neurosensory/neurodevelopmental; motor-development (including CP, DCD); language development; Peer relation/social interaction; 12-36 month; preschool age (4-6 years); school age (5-12 years); adolescence (13-18)  Timing: neonatal period; infancy, childhood and adolescence  Setting: clinical and developmental research setting;  Available data suggests that both direct and indirect mechanisms of cerebellar injury appear to stunt cerebellar growth and adversely affect neurodevelopment | Precheck Critically low |
| Brydges, Christopher R. (2018) | https://dx.doi.org/10.1111/dmcn.13685 | Cognitive outcomes in children and adolescents born very preterm: a meta-analysis | Review Design: Prognostic  Framework: PICOTS  P (Population): children born very preterm; preterms as full population; <28 weeks; 28 - <32 weeks  I / Index / Condition: Gestational age  Comparator / Ref.: comparison with full term  Outcome(s): cognition; preschool age (4-6 years); school age (5-12 years); adolescence (13-18)  Timing: birth; childhood and adolescence  Setting: The intended setting is a clinical and research environment, focusing on understanding the long-term cognitive outcomes of children and adolescents born very preterm. The role of the prognostic factor (very preterm birth) is to assess its impact on cognitive development throughout childhood and adolescence. | Precheck Critically low |
| Buhamer, Shaima Nasser (2021) | https://dx.doi.org/10.1371/journal.pone.0259293 | What is the effect of preterm birth on permanent tooth crown dimensions? A systematic review and meta-analysis | Review Design: Prognostic  Framework: PICOTS  P (Population): preterm; preterms as full population; "preterm" (<37 weeks)  I / Index / Condition: Gestational age  Comparator / Ref.: comparison with full term  Outcome(s): teeth; not reported  Timing: birth; not reported  Setting: The intended setting is likely clinical and research-based, focusing on understanding the impact of preterm birth on dental development, specifically permanent tooth crown dimensions. The role of the prognostic factor (preterm birth) is to assess its association with dental developmental outcomes in individuals born preterm. | ROBIS Low |
| Burkhart, Robert J. (2023) | https://dx.doi.org/10.1097/BPB.0000000000001021 | Is prematurity a risk factor for developmental dysplasia of the hip? A systematic review and meta-analysis | Review Design: Prognostic  Framework: PICOTS  P (Population): preterms; preterms as full population; "preterm" (<37 weeks)  I / Index / Condition: Gestational age  Comparator / Ref.: comparison with full term  Outcome(s): Other: developmental dysplasia of the hip; not reported  Timing: birth; not reported  Setting: gestational prematurity may not be a predisposing risk factor for DDH premature infants had a higher likelihood of having mature hips by the Graf classification. findings suggest that prematuriy of the infant in isolation would not warrant ultrasound assessment of the hip after traditional physical exam maneuvers. | Precheck Critically low |
| Burstein, Or (2021) | https://dx.doi.org/10.1001/jamanetworkopen.2021.3687 | Preterm Birth and the Development of Visual Attention During the First 2 Years of Life: A Systematic Review and Meta-analysis | Review Design: Prognostic  Framework: PICOTS  P (Population): infants born preterm (ie, GA<37 weeks at birth); preterms as full population; "preterm" (<37 weeks)  I / Index / Condition: Gestational age  Comparator / Ref.: comparison with full term  Outcome(s): eyes; Other: visual attention; 0-3 month; 0-12 month; 12-36 month  Timing: birth; first 2 years of life  Setting: Infants born preterm have increased risk for deficits in visual attention, cascading from basic reflexive functions (namely visual-following and latency to fixate) to difficulties in early operations of endogenous attention, such as novelty preference and focused attention.  Early-life markers of attention abnormalities have not been established to date but could provide insights into the pathogenesis of attention abnormalities and could help identify susceptible individuals. | Precheck Critically low |
| Cai, Shirley (2019) | https://dx.doi.org/10.3390/children6120131 | Short- and Long-Term Neurodevelopmental Outcomes of Very Preterm Infants with Neonatal Sepsis: A Systematic Review and Meta-Analysis | Review Design: Prognostic  Framework: PICOTS  P (Population): very preterm infants who had blood culture-proven neonatal sepsis; preterms as full population; <28 weeks; 28 - <32 weeks; VLBW (<1500gr)  I / Index / Condition: neonatal complication (BPD, IVH, PVL, NEC, Sepsis)  Comparator / Ref.: Any other prognostic factor/model; very preterms without sepsis  Outcome(s): neurosensory/neurodevelopmental; Other: starting at 18 months, and had no restriction on the upper limit of follow-up duration  Timing: neonatal period; starting at 18 months, and had no restriction on the upper limit of follow-up duration  Setting: Neonatal sepsis in very preterm infants is associated with increased risk of neurodevelopmental disability. Due to the paucity of longitudinal follow-up data beyond 36 months, the long-term cognitive effect of neonatal sepsis in very preterm infants could not be conclusively determined. | Precheck Critically low |
| Camfferman, Fleur A. (2020) | https://dx.doi.org/10.1038/s41390-020-0777-x | Diagnostic and predictive value of Doppler ultrasound for evaluation of the brain circulation in preterm infants: a systematic review | Review Design: Prognostic  Framework: PICOTS  P (Population): preterm infants (gestational age <37 weeks); preterms as full population; "preterm" (<37 weeks)  I / Index / Condition: Other: Doppler-derived variables in cerebral arteries  Comparator / Ref.: Any other prognostic factor/model; any other Doppler-derived variables in cerebral arteries  Outcome(s): neurosensory/neurodevelopmental; Other: intracranial injury; 12-36 month  Timing: during hospital stay; 12-18 months  Setting: no clear evidence to support the routine use of RI or other arterial Doppler-derived parameters to predict neurological outcome in the preterm infant;  there is some evidence that elevated RI in the ACA and MCA can point to the presence of a hemodynamically significant PDA | Precheck Critically low |
| Casavant, Sharon G. (2019) | https://dx.doi.org/10.1177/1099800418824415 | Allostatic Load and Biomarkers of Stress in the Preterm Infant: An Integrative Review | Review Design: Prognostic  Framework: PICOTS  P (Population): preterm infants exposed to repeated stressful and/or painful procedures in the NICU; preterms as full population; "preterm" (<37 weeks)  I / Index / Condition: Other: biomarkers  Comparator / Ref.: comparison with full term  Outcome(s): Other: markers of stress in later life; school age (5-12 years); not reported  Timing: neonatal period; infancy and childhood  Setting: The interaction of disease with therapeutic interventions may inadvertently increase infant allostatic load. | Precheck Critically low |
| Casavant, Sharon G. (2019) | https://dx.doi.org/10.1016/j.earlhumdev.2019.03.003 | Associations between preterm infant stress, epigenetic alteration, telomere length and neurodevelopmental outcomes: A systematic review | Review Design: Prognostic  Framework: PICOTS  P (Population): preterm infants ex- posed to repeated stressful and/or painful procedures in the NICU; preterms as full population; "preterm" (<37 weeks)  I / Index / Condition: Other: repeated stressful and/or painful procedures  Comparator / Ref.: comparison with full term; Any other prognostic factor/model; different GA  Outcome(s): Other: (1) epigenetic change as a result of repeated painful or stressful experiences, (2) telomere length changes; not reported  Timing: neonatal period; not reported  Setting: Several putative epigenetic markers were identified although there was a paucity of studies related to telomere length. The interaction of disease entity combined with therapeutic interventions intended to treat may inadvertently increase infant allostatic load or ability to adapt to stress. Future research should include not only human studies but leverage newly available large data sets to conduct additional analysis. | Precheck Critically low |
| Cassiano, Rafaela G. M. (2016) | https://dx.doi.org/10.1002/imhj.21563 | PREMATURITY, NEONATAL HEALTH STATUS, AND LATER CHILD BEHAVIORAL/EMOTIONAL PROBLEMS: A SYSTEMATIC REVIEW | Review Design: Prognostic  Framework: PICOTS  P (Population): children born preterm until 12 years of age; preterms as full population; "preterm" (<37 weeks)  I / Index / Condition: Gestational age; neonatal complication (BPD, IVH, PVL, NEC, Sepsis)  Comparator / Ref.: comparison with full term  Outcome(s): behaviour/mental health (include ADHD, autism); 12-36 month; preschool age (4-6 years); school age (5-12 years)  Timing: birth and neonatal period; until the age of 12  Setting: the neonatal health problems associated with prematurity present a negative impact on later child emotional and adapted behavior. | Precheck Critically low |
| Cebeci, Burcu (2022) | https://dx.doi.org/10.1038/s41390-021-01539-x | Brain proton magnetic resonance spectroscopy and neurodevelopment after preterm birth: a systematic review | Review Design: Prognostic  Framework: PICOTS  P (Population): Studies included in this systematic review were composed of variable populations involving both a small number (range 12-43) and a large number of infants (range 65-177) born at or below 32 gestational weeks. The number of infants that participated in the majority of the studies was <30. More than half of the studies had a control group consisting of term babies to compare with the preterm infants; preterms as full population; "preterm" (<37 weeks)  I / Index / Condition: Other: MRI features  Comparator / Ref.: not reported  Outcome(s): Other: various single outcomes; 12-36 month; preschool age (4-6 years); school age (5-12 years); adolescence (13-18)  Timing: Time point of prognostication is age at H-MRS Scan. Differs between shortly after birth to 8,4y. (table 1); not reported  Setting: Preterm infants are at risk of neurodevelopmental impairments. At present, proton magnetic resonance spectroscopy (H-MRS) is used to evaluate brain metabolites in asphyxiated term infants. H-MRS is a potential surrogate end point for neurodevelopment in preterm infants: NAA/Cho ratio in WM at term equivalent age is associated with motor outcome in preterm infants at 18-24 months corrected age. NAA/Cho ratios in the WM were associated with cognitive scores and NAA/Cho ratios in the WM and GM were significantly related to language scores in preterm born infants assessed at 18-24 months corrected age. | Precheck Critically low |
| Chan, E. (2016) | https://dx.doi.org/10.1111/cch.12320 | Long-term cognitive and school outcomes of late-preterm and early-term births: a systematic review | Review Design: Prognostic  Framework: PICOTS  P (Population): late-preterm and early-term births; preterms as subgroup; Other: 34-36 weeks  I / Index / Condition: Gestational age  Comparator / Ref.: comparison with full term  Outcome(s): cognition; Education; Other: beyond 2 years of age  Timing: birth; 2 years of age and older  Setting: The review indicates the importance of careful monitoring and observation for children born at Late Preterm (LPT) or Early Term (ET). This is to identify any challenges they may face in cognitive and school-related areas. | Precheck Critically low |
| Choi, Jae Hong (2022) | https://dx.doi.org/10.3346/jkms.2022.37.e35 | Risk Factors for Severe COVID-19 in Children: A Systematic Review and Meta-Analysis | Review Design: Prognostic  Framework: PICOTS  P (Population): children with COVID-19; preterms as subgroup; "preterm" (<37 weeks)  I / Index / Condition: Gestational age  Comparator / Ref.: comparison with full term  Outcome(s): infections (RSV, ...); 0-12 month; 12-36 month  Timing: birth; first two years of age  Setting: The setting is primarily hospital-based, given that the outcomes measured include ICU admission and invasive mechanical ventilation. The role of the prognostic factors is to identify children at high risk of severe COVID-19, guiding hospital admission and vaccination priorities. Premature infants had a high risk  of severe COVID-19. | Precheck Critically low |
| Christians, Julian K. (2023) | https://dx.doi.org/10.1186/s13293-023-00532-9 | Sex differences in the effects of prematurity and/or low birthweight on neurodevelopmental outcomes: systematic review and meta-analyses | Review Design: Prognostic  Framework: PICOTS  P (Population): humans born premature and/or of low birthweight; preterms as full population; "preterm" (<37 weeks)  I / Index / Condition: Gestational age; Other: sex  Comparator / Ref.: comparison with full term; Any other prognostic factor/model; comparison of the sexes  Outcome(s): behaviour/mental health (include ADHD, autism); cognition; neurosensory/neurodevelopmental; language development; 12-36 month; preschool age (4-6 years); school age (5-12 years); adolescence (13-18)  Timing: birth; 1 year of age or older  Setting: The study aimed to assess whether the effects of prematurity and/or low birthweight on neurodevelopmental outcomes were greater in one sex than the other.  We found no evidence that the sexes differ in their susceptibility to the effects of severe or moderate  prematurity/low birthweight on cognitive function, internalizing traits or externalizing traits. | Precheck Critically low |
| Cogley, Clodagh (2021) | https://dx.doi.org/10.1007/s10578-020-01071-9 | A Systematic Review of the Risk Factors for Autism Spectrum Disorder in Children Born Preterm | Review Design: Prognostic  Framework: PICOTS  P (Population): born less than 37 weeks gestation or with low birth weight (≤ 2500 g), born after January 1st, 1990, diagnosed autism, participants <18 years at assessment for ASD; preterms as full population; "preterm" (<37 weeks)  I / Index / Condition: Gestational age; Other: maternal diabetes  Comparator / Ref.: comparison with full term; Any other prognostic factor/model; male sex, being small for gestational age, and cognitive impairment  Outcome(s): behaviour/mental health (include ADHD, autism); 12-36 month; preschool age (4-6 years); school age (5-12 years); adolescence (13-18)  Timing: birth; >12mo  Setting: The review was intended to synthesize existing literature to identify prenatal, perinatal, and postnatal factors associated with an increased risk of ASD in preterm children. The setting was a systematic review of existing studies, rather than a specific clinical or healthcare setting. | Precheck Critically low |
| Cui, Yuqi (2023) | https://dx.doi.org/10.1111/idh.12651 | The association between low birth weight and/or preterm birth and dental caries -A systematic review and meta-analysis | Review Design: Prognostic  Framework: PICOTS  P (Population): children under 6 years of age with primary dentition, with no restrictions on gender, race, culture, or socio-economic status; preterms as subgroup; "preterm" (<37 weeks)  I / Index / Condition: Gestational age  Comparator / Ref.: comparison with full term  Outcome(s): teeth; 0-12 month; 12-36 month; preschool age (4-6 years); school age (5-12 years)  Timing: birth; until the age of 6  Setting: The current evidence did not suggest a significant association between  LBW and dental caries in children for primary teeth. Children with PTB in high-income  countries had a higher prevalence of primary dental caries. | Precheck Critically low |
| Dahan-Oliel, Noemi (2012) | https://dx.doi.org/10.1016/j.ridd.2012.02.011 | Preterm birth and leisure participation: a synthesis of the literature | Review Design: Prognostic  Framework: PICOTS  P (Population): gestational age <37 weeks and/or birthweight <1500 g; preterms as full population; "preterm" (<37 weeks); VLBW (<1500gr)  I / Index / Condition: Gestational age  Comparator / Ref.: comparison with full term  Outcome(s): Participation in activites; preschool age (4-6 years); school age (5-12 years); adolescence (13-18)  Timing: birth; childhood, adolescence  Setting: In school-age children, no significant differences were found in activity levels between children born prematurely and term born controls. In adolescents, leisure scores in social activities, hobbies and sports were statistically significantly lower compared to controls. In young adults, differences in frequency and intensity of physical activity were reported compared to term controls. Promoting participation in leisure activities should be encouraged at a young age and continued to adulthood by minimizing the gaps between capabilities and the demands of the tasks and the environment. | Precheck Critically low |
| Dalmartello, Michela (2023) | https://dx.doi.org/10.1016/j.earlhumdev.2023.105816 | A systematic review on maternal and perinatal factors influencing breast development | Review Design: Prognostic  Framework: PICOTS  P (Population): female human; preterms as subgroup; "preterm" (<37 weeks)  I / Index / Condition: Gestational age  Comparator / Ref.: not reported  Outcome(s): Other: Breast development, breast onset (attainment of thelarche or B2 Tanner stage), or age at thelarche attainment, or breast development through the different Tanner stages, and type of puberty initiation (thelarche vs pubarche vs synchronous); school age (5-12 years); adolescence (13-18)  Timing: birth; puberty  Setting: Breast development is a key physical marker of puberty onset, and early puberty development is linked to consequences that can reverÂ­berate throughout life. Answering questions about the interconnections of environmental exposures in pre/postnatal period and their impact on puberty represents an important area of multidisciplinary research | Precheck Critically low |
| de Bruijn, Clara Adriana Maria (2023) | https://dx.doi.org/10.1038/s41390-022-02232-3 | Neurodevelopmental consequences of preterm punctate white matter lesions: a systematic review | Review Design: Prognostic  Framework: PICOTS  P (Population): infants born preterm (i.e., born <37 weeks of gestation) who were diagnosed with punctate white matter lesions (PWML) on MRI scans made around the term date; preterms as full population; "preterm" (<37 weeks)  I / Index / Condition: Other: presence of punctate white matter lesions (PWML)  Outcome(s): neurosensory/neurodevelopmental; 12-36 month; school age (5-12 years); adolescence (13-18)  Timing: around the term-equivalent age; at a minimum age of 12 months  Setting: The intended setting is the long-term neurodevelopmental assessment of preterm infants diagnosed with PWML. The role of the prognostic factor (PWML) is to evaluate its influence on neurodevelopmental outcomes in these infants. PWML is common in preterm infants and predictive of adverse NDO, in particular on motor outcomes and less on cognitive and behavioral outcomes. The type and severity of impairments are related to the number and location of PMWL. | Precheck Critically low |
| de Jong, Femke (2012) | https://dx.doi.org/10.1161/HYPERTENSIONAHA.111.181784 | Systematic review and meta-analysis of preterm birth and later systolic blood pressure | Review Design: Prognostic  Framework: PICOTS  P (Population): former preterm or VLBW children or adolescents; preterms as full population; "preterm" (<37 weeks); VLBW (<1500gr)  I / Index / Condition: Gestational age  Comparator / Ref.: comparison with full term  Outcome(s): blood pressure/cardiovascular; preschool age (4-6 years); school age (5-12 years); adolescence (13-18)  Timing: birth; childhood, adolescence, adulthood  Setting: infants who are born preterm or very low birth weight have modestly higher systolic blood pressure later in life and may be at increased risk for developing hypertension and its sequela | Precheck Critically low |
| de Kieviet, Jorrit F. (2012) | https://dx.doi.org/10.1111/j.1469-8749.2011.04216.x | Brain development of very preterm and very low-birthweight children in childhood and adolescence: a meta-analysis | Review Design: Prognostic  Framework: PICOTS  P (Population): children with a gestational age less than 32 weeks and/or a birthweight less than 1500 g; preterms as full population; 28 - <32 weeks; VLBW (<1500gr)  I / Index / Condition: Gestational age  Comparator / Ref.: comparison with full term  Outcome(s): cognition; neurosensory/neurodevelopmental; motor-development (including CP, DCD); language development; Other: brain volume; school age (5-12 years); adolescence (13-18)  Timing: birth; school age and adolescence  Setting: Very preterm/VLBW birth is associated with an overall reduction in brain volume, which becomes evident in equally sized reductions in white and grey matter volumes, as well as in volumes of diverse brain structures throughout childhood and adolescence.  Future studies should extend their scope to complementary neurocognitive domains and imaging techniques to further clarify the underlying mechanism for the widespread developmental problems following very preterm/VLBW birth. | Precheck Critically low |
| de Paula Eduardo, Juliana Arantes Figueiredo (2019) | https://dx.doi.org/10.1016/j.jad.2019.08.069 | Preterm birth as a risk factor for postpartum depression: A systematic review and meta-analysis | Review Design: Prognostic  Framework: PICOTS  P (Population): mothers of preterm children; Other: not applicable, for the participants were adult women; Other: not applicable, for the participants were adult women  I / Index / Condition: Other: preterm birth  Comparator / Ref.: Any other prognostic factor/model; mothers of full-term infants  Outcome(s): Other: postpartum depression; Other: within a year after giving birth  Timing: birth; within one year after childbirth  Setting: importance of maternal mental health care in this target population, as preterm birth experience seem to affect both babies and mothers | Precheck Critically low |
| Dean, Bethan (2021) | https://dx.doi.org/10.1016/j.neubiorev.2021.01.006 | Social cognition following preterm birth: A systematic review | Review Design: Prognostic  Framework: PICOTS  P (Population): Participants were children born preterm (before 36 completed weeks  gestation) who had undergone directly observed testing measures of  social cognition before six years of age.; preterms as full population; "preterm" (<37 weeks)  I / Index / Condition: Gestational age  Comparator / Ref.: comparison with full term  Outcome(s): behaviour/mental health (include ADHD, autism); cognition; Parent-Child-Interaction; Peer relation/social interaction; 0-12 month; 12-36 month; preschool age (4-6 years)  Timing: birth; before the age of 6  Setting: We detected a pattern  of reduced social attention in the first 12 months of life with evidence of reduced performance in social cognitive  tasks later in the preschool years. However, we did not identify a consistent, distinctive preterm social phenotype  in early life. Instead, the interactive behaviour of preterm infants reflects factors from outside the social cognitive  domain, such as attention, language, and socioeconomic status. | Precheck Critically low |
| Depoorter, Antoinette (2018) | https://dx.doi.org/10.1016/j.neubiorev.2018.02.011 | Predicting neurodevelopmental outcome in preterm born infants using auditory event-related potentials: A systematic review | Review Design: Prognostic  Framework: PICOTS  P (Population): prematurely born participants; preterms as full population; "preterm" (<37 weeks)  I / Index / Condition: Other: auditory event-related potentials (AERPs) in children born preterm  Comparator / Ref.: comparison with full term  Outcome(s): Lung; cognition; language development; 0-3 month; 0-12 month; 12-36 month; preschool age (4-6 years); school age (5-12 years)  Timing: infancy and childhood. The AERP measurements were taken at various times, ranging from neonatal age to up to 9 years of age.; at the same time (?)  Setting: The intended setting was clinical, with the role of AERPs being to assess and predict cognitive functioning and language development in children born preterm.  Both prospective and cross-sectional studies reported a relationship between AERPs and cognitive outcome. Our results show that larger amplitudes and shorter latencies of late AERPs are related to better cognitive outcomes. Additional studies are needed to corroborate our findings re- garding this potential use of AERPs in the individual evaluation of preterm born infants. | Precheck Critically low |
| DiFranza, Joseph R. (2012) | https://dx.doi.org/10.1186/1471-2431-12-81 | Systematic literature review assessing tobacco smoke exposure as a risk factor for serious respiratory syncytial virus disease among infants and young children | Review Design: Prognostic  Framework: PICOTS  P (Population): infants and children up to 5 years of age, including studies of children at high risk for serious RSV disease. We defined the high-risk population as patients with prematurity, BPD, or congenital heart disease; preterms as subgroup; <28 weeks; 28 - <32 weeks; Other: <35 weeks  I / Index / Condition: Other: tobacco smoke exposure  Comparator / Ref.: Any other prognostic factor/model; children not defined as high risk, defined at high risk (other causes: BPD, or congenital heart disease)  Outcome(s): infections (RSV, ...); readmission to hospital; 0-12 month; 12-36 month; preschool age (4-6 years)  Timing: birth; up to 5 years of age  Setting: ample evidence that ETS exposure places infants and young children at increased risk of hospitalization for RSV-attributable LRTIs, and increases the severity of illness among children hospitalized for RSV.   The prevention of serious RSV illness provides one more rationale for protecting infants and young children from exposure to tobacco smoke, especially high-risk groups such as premature infants and those with chronicconditions who are considered at increased risk of serious RSV disease | Precheck Critically low |
| Diggikar, Shivashankar (2022) | https://dx.doi.org/10.1002/ppul.26128 | Respiratory infections in children born preterm in low and middle-income countries: A systematic review | Review Design: Prognostic  Framework: PICOTS  P (Population): Preterm infants less than 37 weeks of gestational age (GA) born in LMICs; preterms as full population; "preterm" (<37 weeks)  I / Index / Condition: Gestational age  Comparator / Ref.: comparison with full term; Any other prognostic factor/model; In descriptive studies, there was no comparatorâ€‹  Outcome(s): Lung; readmission to hospital; Other: during the first 2 years of life  Timing: birth; during the first 2 years of life  Setting: studies from LMICs shows that children born preterm are at significantly higher risk of developing any respiratory infection and also highlights a substantial variation in the risk of acute lower respiratory tract infections, RSV infections, and respiratory infections requiring hospitalization or emergency care treatment and a considerable increase in length of hospital stay, which therefore underlines the need for preventive strategies, including RSV immunoprophylaxis | Precheck Critically low |
| Diggikar, Shivashankar (2023) | https://dx.doi.org/10.3389/fped.2023.1055813 | Retinopathy of prematurity and neurodevelopmental outcomes in preterm infants: A systematic review and meta-analysis | Review Design: Prognostic  Framework: PICOTS  P (Population): preterm infants (<37 weeks) with any ROP (type 1 or severe ROP, type 2 or milder ROP, laser or anti-VEGF treatment); preterms as full population; "preterm" (<37 weeks)  I / Index / Condition: neonatal complication (BPD, IVH, PVL, NEC, Sepsis)  Comparator / Ref.: Any other prognostic factor/model; Preterm infants without ROP  Outcome(s): behaviour/mental health (include ADHD, autism); cognition; neurosensory/neurodevelopmental; motor-development (including CP, DCD); language development; Other: between the ages of 18 and 48 months Cognitive impairment (6 months to 21 years) Motor function evaluated above 4 years  Timing: neonatal period; infancy, childhood and adolescence  Setting: Infants with â€œany ROPâ€ had higher risks of cognitive impairment or intellectual disability, cerebral palsy, and behavioural problems. Anti-VEGF treatment increased the risk of moderate cognitive impairment. These results support the association of ROP and anti-VEGF treatment with adverse neurodevelopmental outcomes. | Precheck Critically low |
| Ding, Sharon (2019) | https://dx.doi.org/10.1111/apa.14693 | A meta-analysis of neurodevelopmental outcomes at 4-10 years in children born at 22-25 weeks gestation | Review Design: Prognostic  Framework: PICOTS  P (Population): children born at 22-25 weeks gestation; preterms as full population; <28 weeks  I / Index / Condition: Gestational age  Comparator / Ref.: N/A; comparison with full term  Outcome(s): neurosensory/neurodevelopmental; preschool age (4-6 years); school age (5-12 years)  Timing: birth; 4-10 years of age  Setting: The prognostic factors, primarily gestational age, were used in a clinical research context to predict long-term neurodevelopmental outcomes in extremely preterm infants. This information is crucial for healthcare providers and parents in understanding and anticipating the potential challenges and support needs of these children as they grow. | Precheck Critically low |
| Ding, Yuan (2020) | https://dx.doi.org/10.1007/s12519-019-00327-2 | Risk factors for infantile hemangioma: a meta-analysis | Review Design: Prognostic  Framework: PICOTS  P (Population): patients with infantile hemangioma (IH); preterms as subgroup; "preterm" (<37 weeks)  I / Index / Condition: Gestational age  Outcome(s): Other: infantile hemangioma; not reported  Timing: birth (in case of the risk factor prematurity); not reported  Setting: The study aimed to identify risk factors for IH. The setting is clinical, focusing on understanding the factors that increase the risk of developing IH. | Precheck Critically low |
| Domellof, Erik (2011) | https://dx.doi.org/10.1016/j.neuropsychologia.2011.04.033 | Handedness in preterm born children: a systematic review and a meta-analysis | Review Design: Prognostic  Framework: PICOTS  P (Population): children born preterm and fullterm control children, aged 3-19 years; preterms as full population; "preterm" (<37 weeks)  I / Index / Condition: Gestational age; Other: non-right-handedness (NRH)  Comparator / Ref.: comparison with full term  Outcome(s): Other: handedness; 12-36 month; preschool age (4-6 years); school age (5-12 years); adolescence (13-18)  Timing: birth; 3-18 years of age  Setting: The study aimed to understand the association between preterm birth and the development of NRH, suggesting an early disturbance in the typical development of cerebral asymmetry in preterm children. | Precheck Critically low |
| Duan, Kai (2017) | https://dx.doi.org/10.1007/s12098-016-2248-2 | Safety and Immunogenicity of Pneumococcal Conjugate Vaccine in Preterm Infants: A Meta-Analysis | Review Design: Prognostic  Framework: PICOTS  P (Population): preterm with pneumococcal conjugate vaccination; preterms as full population; "preterm" (<37 weeks); VLBW (<1500gr)  I / Index / Condition: Gestational age  Comparator / Ref.: comparison with full term  Outcome(s): Other: vaccination immunogenicity; 0-12 month; 12-36 month  Timing: birth; infancy  Setting: The meta-analysis suggests that the preterm infants have a great tolerance to PCV-7, PCV-10 or PCV-13. Despite of the difference of antigen content and carrier protein in the PCVs, vaccination with PCV-7, PCV-10 or PCV-13 can elicit robust immunogenicity against seven common serotypes 4, 6B, 9 V, 14, 18C, 19F and 23F, and three additional serotypes 1, 5 and 7F. | Precheck Critically low |
| Edwards, Jessica (2011) | https://dx.doi.org/10.1097/DBP.0b013e31822a396a | Developmental coordination disorder in school-aged children born very preterm and/or at very low birth weight: a systematic review | Review Design: Prognostic  Framework: PICOTS  P (Population): infants at school age (5-18 years) born very preterm (<32 weeks) and/or VLWB (<1500g); preterms as full population; <28 weeks; 28 - <32 weeks; VLBW (<1500gr); ELBW (<1000gr)  I / Index / Condition: Gestational age; Other: birth weight  Comparator / Ref.: comparison with full term  Outcome(s): motor-development (including CP, DCD); school age (5-12 years); adolescence (13-18)  Timing: birth; school age period (5â€“18 years)  Setting: Clinical practice should focus on early identification of and intervention for children with DCD, while research should focus on determining the mechanisms underlying DCD in the preterm population. | Precheck Critically low |
| Edwards, Martin O. (2015) | https://dx.doi.org/10.1002/ppul.23117 | Effect of preterm birth on exercise capacity: A systematic review and meta-analysis | Review Design: Prognostic  Framework: PICOTS  P (Population): preterms with or without BPD; preterms as full population; "preterm" (<37 weeks)  I / Index / Condition: Gestational age; neonatal complication (BPD, IVH, PVL, NEC, Sepsis)  Comparator / Ref.: comparison with full term; Any other prognostic factor/model; subgroups: preterms with or without BPD, without BPD, BPD28, BPD36  Outcome(s): Lung; Other: exercise capacity; Other: (from 5 to 21 years old)  Timing: birth; childhood and adolescence (5-21 years old)  Setting: large amount of heterogeneity in the included studies preterm-born subjects with or without BPD have lower (<13%) exercise capacity than term-born control subjects.  The differences in VO2max are small and it would appear that despite the increased risk of cardio-respiratory problems of preterm-born subjects with or without BPD, they are able to achieve near normal exercise capacity. data suggest continual remodeling of the pulmonary alveolarâ€“capillary apparatus as suggested by recent reports of alveolar development | Precheck Critically low |
| Elmrayed, Seham (2023) | https://dx.doi.org/10.1111/ppe.13002 | Small for gestational age preterm infants and later adiposity and height: A systematic review and meta-analysis | Review Design: Prognostic  Framework: PICOTS  P (Population): preterms with and without SGA (small for gestational age); preterms as full population; "preterm" (<37 weeks)  I / Index / Condition: Gestational age; Other: SGA  Comparator / Ref.: Any other prognostic factor/model; non-SGA  Outcome(s): growth/metabolism; 12-36 month; preschool age (4-6 years); school age (5-12 years); adolescence (13-18)  Timing: birth; >24 months of age  Setting: Compared to their preterm non-SGA peers, preterm infants born SGA  have lower BMI, waist circumference, lean body mass and height in later life. No dif- ferences in adiposity were observed between SGA preterm infants and non-SGA pre- term infants. | ROBIS Low |
| Engeseth, Merete Salveson (2018) | https://dx.doi.org/10.1016/j.prrv.2017.11.001 | Left vocal cord paralysis after patent ductus arteriosus ligation: A systematic review | Review Design: Prognostic  Framework: PICOTS  P (Population): extremely premature infants born at gestational age <28 weeks and/or <1000g birthweight diagnosed with PDA; preterms as full population; <28 weeks; ELBW (<1000gr)  I / Index / Condition: Other: surgical PDA ligation  Outcome(s): Lung; neurosensory/neurodevelopmental; readmission to hospital; Other: left vocal cord paralysis; 0-12 month; 12-36 month  Timing: neonatal period; early childhood  Setting: LVCP is associated with negative outcomes in EP infants. The understanding of long-term outcomes is scarce. | Precheck Critically low |
| Falsaperla, Raffaele (2022) | https://dx.doi.org/10.1684/epd.2021.1379 | Beyond neonatal seizures - epileptic evolution in preterm newborns: a systematic review and meta-analysis | Review Design: Prognostic  Framework: PICOTS  P (Population): preterm newborns; preterms as subgroup; "preterm" (<37 weeks)  I / Index / Condition: Gestational age; neonatal complication (BPD, IVH, PVL, NEC, Sepsis)  Comparator / Ref.: comparison with full term  Outcome(s): Epilepsy; 0-3 month; 0-12 month; 12-36 month; preschool age (4-6 years); school age (5-12 years)  Timing: neonatal period; infancy and childhood  Setting: The study focused on the neonatal and post-neonatal period in a hospital setting (post-NICU follow-up). The role of prognostic factors like gestational age and the presence of neonatal seizures was to assess the risk of developing PNE in this population. | Precheck Critically low |
| Fan, Wei (2020) | https://dx.doi.org/10.1016/j.amepre.2020.03.009 | Immunogenicity of Hepatitis B Vaccine in Preterm or Low Birth Weight Infants: A Meta-Analysis | Review Design: Prognostic  Framework: PICOTS  P (Population): infants born preterm; preterms as full population; "preterm" (<37 weeks)  I / Index / Condition: Gestational age  Comparator / Ref.: comparison with full term  Outcome(s): Other: immune response to HBvac; Other: 1-60 month after vaccination  Timing: birth; childhood  Setting: The intended setting is a comparison of immunogenic response to hepatitis B vaccination in preterm versus full-term infants. The findings suggest an association between preterm birth and lowered immune responses to hepatitis B vaccine. | Precheck Critically low |
| Fenton, Tanis R. (2023) | https://dx.doi.org/10.1111/ppe.12955 | Small for date preterm infants and risk of higher blood pressure in later life: A systematic review and meta-analysis | Review Design: Prognostic  Framework: PICOTS  P (Population): preterm SGA or non-SGA; preterms as full population; "preterm" (<37 weeks)  I / Index / Condition: Other: SGA  Comparator / Ref.: Any other prognostic factor/model; preterm non-SGA  Outcome(s): blood pressure/cardiovascular; 12-36 month; preschool age (4-6 years); school age (5-12 years); adolescence (13-18); Other: <41 years  Timing: birth; age group (age 2-6, 7-18, adult: 19 years or older) greater)  Setting: individuals born preterm SGA do not have higher blood pressure late in life compared to preterm infants who were not SGA | ROBIS High |
| Festante, F. (2019) | https://dx.doi.org/10.1155/2019/5759694 | Parent-Infant Interaction during the First Year of Life in Infants at High Risk for Cerebral Palsy: A Systematic Review of the Literature | Review Design: Prognostic  Framework: PICOTS  P (Population): infants at high risk for neurological impairment and either healthy controls or low-risk populations,; preterms as subgroup; "preterm" (<37 weeks); VLBW (<1500gr)  I / Index / Condition: Gestational age; neonatal complication (BPD, IVH, PVL, NEC, Sepsis)  Comparator / Ref.: comparison with full term; Any other prognostic factor/model; infants without high neurological risks  Outcome(s): Parent-Child-Interaction; 0-3 month; 0-12 month  Timing: birth and neonatal period; first year of life  Setting: The intended setting for these prognostic factors is in neonatal and early childhood care, with a focus on identifying high-risk infants who might benefit from early interventions. The role of these factors is to help predict developmental outcomes and guide interventions that can support infants' developmental needs. The articles represent a high level of heterogeneity in terms of infant neurological risk, infant age, and tools assessing interactive behaviors. Both infant and maternal behaviors within the investigated interactive exchanges were reported to be compromised, leading to subsequent overall impairment of the dyadic patterns. | Precheck Critically low |
| Figueras-Aloy, Josep (2016) | nan | Defining the Risk and Associated Morbidity and Mortality of Severe Respiratory Syncytial Virus Infection Among Preterm Infants Without Chronic Lung Disease or Congenital Heart Disease | Review Design: Prognostic  Framework: PICOTS  P (Population): preterms without CLD or CHD who had RSV; preterms as full population; "preterm" (<37 weeks)  I / Index / Condition: Gestational age; Other: chronological age less than 3 months at the onset of the RSV season, CLD, living with school-age siblings, and exposure to tobacco smokeâ€‹  Comparator / Ref.: comparison with full term; Any other prognostic factor/model; different GA  Outcome(s): Lung; infections (RSV, ...); post-discharge death; readmission to hospital; Other: health care resource utilization; 0-3 month; 0-12 month; 12-36 month  Timing: birth; under 2 years of age  Setting: The intended setting and role of the prognostic factors were to identify and assess risk factors, including biological, environmental, and social, for severe RSV infection requiring hospital admission in preterm infants. Predictive models can identify 32/33â€“35 wGA infants at risk of RSVH (high SOE). Preterm infants, particularly those born at lower wGA, tended to have higher RSV hospitalization (RSVH) rates compared with otherwise healthy term infants (high SOE). | Precheck Critically low |
| FitzGerald, Tara L. (2018) | https://dx.doi.org/10.1093/ptj/pzy050 | Body Structure, Function, Activity, and Participation in 3- to 6-Year-Old Children Born Very Preterm: An ICF-Based Systematic Review and Meta-Analysis | Review Design: Prognostic  Framework: PICOTS  P (Population): 3- to 6-year-old children born VP (<32 weeksâ€™ gestation or birth weight <1500 g); preterms as full population; <28 weeks; 28 - <32 weeks  I / Index / Condition: Gestational age  Comparator / Ref.: comparison with full term  Outcome(s): motor-development (including CP, DCD); Participation in activites; Other: body structure and function, activity,; 12-36 month; preschool age (4-6 years)  Timing: birth; preschool age  Setting: There is increasing awareness of  mild to moderate subsequent motor im- pairment in this population, and appre- ciation that even minor motor impair- ment can have implications for other  areas of functioning, including cogni- tion, academic ability, and behavior.  importance of the  multidisciplinary team in developmen- tal surveillance, and specify the involve- ment of physical therapists. Physical  therapists have an important role in  identifying and treating the motor im- pairments of children born VP, and in  developmental surveillance from infan- cy to preschool age.   preschool-aged children  born VP have poorer motor outcomes  compared with children born FT with- in the ICF-CY domains of body struc- ture and function and activity. However,  participation of children born VP com- pared with their term-born peers at  preschool age is not well described.  Clinically, physical therapists should  consider all ICF domains when assess- ing and treating preschool-aged chil- dren born VP, and advocate for develop- mental surveillance into preschool age | Precheck Critically low |
| Franz, Adelar Pedro (2018) | https://dx.doi.org/10.1542/peds.2017-1645 | Attention-Deficit/Hyperactivity Disorder and Very Preterm/Very Low Birth Weight: A Meta-analysis | Review Design: Prognostic  Framework: PICOTS  P (Population): VP, VLBW, EP, or ELBW; preterms as full population; <28 weeks; 28 - <32 weeks; VLBW (<1500gr); ELBW (<1000gr)  I / Index / Condition: Gestational age; Other: LBW  Comparator / Ref.: comparison with full term  Outcome(s): behaviour/mental health (include ADHD, autism); 12-36 month; preschool age (4-6 years); school age (5-12 years); adolescence (13-18); Other: adult  Timing: birth; childhood, adolescence, adulthood  Setting: robust evidence that VP/VLBW individuals have an increased risk of ADHD both in categorical and dimensional analyses, and there is an even stronger association in the EP/ELBW group.   In terms of clinical applicability, we suggest that premature infants need specific neonatology, pediatric,  and psychiatric prevention and management interventions to minimize the ADHD burden.   Future researchers in this field should clarify specific causal determinants associated with prematurity and LBW that could lead to the development of ADHD | ROBIS Low |
| Geldof, C. J. A. (2012) | https://dx.doi.org/10.1016/j.ridd.2011.08.025 | Visual perception and visual-motor integration in very preterm and/or very low birth weight children: a meta-analysis | Review Design: Prognostic  Framework: PICOTS  P (Population): very preterm born (GA <= 32 weeks) and/or VLBW (BW <= 1500 g) children; preterms as full population; Other: very preterm born (GA <= 32 weeks) and/or VLBW (BW <= 1500 g) children  I / Index / Condition: Gestational age  Comparator / Ref.: comparison with full term; Any other prognostic factor/model; Norm scores for children between 4 and 13 years of age (JLO: 7-14 years)  Outcome(s): eyes; Other: visual-motor integration (Judgment of Line Orientation, Gestalt Closure subtest, Motor-Free Visual Perception Test Revised, Arrow subtest of the Developmental Neuropsychological Assessment battery, Test of Visual Perceptual Skills Revised); school age (5-12 years); adolescence (13-18)  Timing: birth; 6 to16 years  Setting: medium to large-sized impairments in visual perceptive abilities, particularly in visual-spatial perception. In addition, medium-sized visual-motor integration deficits were observed that persist from early childhood into adolescence.  Impairments in visual-motor integration were inversely related to GA and were more pronounced in boys than in girls.  Our findings highlight the importance of extensive follow-up of visual perceptive and visual-motor abilities Future studies should investigate whether visual perceptive and visual-motor integration dysfunctions are associated with, and possibly causal of other impairments observed in very preterm/VLBW children, including motor impairments, cognitive deficits, behavioral and emotional difficulties, and lower academic achievement.  Future research should elucidate underlying mechanisms and focus on prevention and possibilities for remediation | Precheck Critically low |
| Ghaseminejad-Raeini, Amirhossein (2023) | https://dx.doi.org/10.1186/s12887-023-04083-1 | Preterm birth does not increase the risk of developmental dysplasia of the Hip: a systematic review and meta-analysis | Review Design: Prognostic  Framework: PICOTS  P (Population): preterm infants; preterms as full population; "preterm" (<37 weeks)  I / Index / Condition: Gestational age  Comparator / Ref.: comparison with full term  Outcome(s): Other: dysplasia of the hip; 0-3 month  Timing: birth; not reported  Setting: did not find preterm birth to be a significant risk factor for DDH.  Data suggests that female sex and breech presentation are associated with DDH in preterm infants, as they are in term infants. focus the healthcare resources, including ultrasound screening of the newborns, to patients who are at a greater risk for DDH, | Precheck Critically low |
| Gill, Peter J. (2015) | https://dx.doi.org/10.1016/S2213-2600(14)70252-8 | Identification of children at risk of influenza-related complications in primary and ambulatory care: a systematic review and meta-analysis | Review Design: Prognostic  Framework: PICOTS  P (Population): children presenting in primary or ambulatory care with influenza or influenza-like illness; preterms as subgroup; "preterm" (<37 weeks)  I / Index / Condition: Gestational age; Other: BPD  Outcome(s): readmission to hospital; 0-3 month; 0-12 month; 12-36 month; preschool age (4-6 years); school age (5-12 years); adolescence (13-18)  Timing: birth; children aged up to 18 years  Setting: We identified prematurity as a new strong risk factor for infl uenza-related complications in children. Insufficient data for BPD | Precheck Critically low |
| Gladstone, Melissa (2015) | https://dx.doi.org/10.1371/journal.pone.0120566 | Survival, morbidity, growth and developmental delay for babies born preterm in low and middle income countries - a systematic review of outcomes measured | Review Design: Prognostic  Framework: PICOTS  P (Population): prematurely born infant in any low and mid- dle income country; preterms as full population; "preterm" (<37 weeks)  I / Index / Condition: Gestational age  Comparator / Ref.: comparison with full term  Outcome(s): growth/metabolism; cognition; neurosensory/neurodevelopmental; motor-development (including CP, DCD); language development; post-discharge death; Other: morbidity; 0-12 month; 12-36 month; preschool age (4-6 years); school age (5-12 years); adolescence (13-18)  Timing: birth; up to 15 years of age  Setting: To be able to determine the relative contribution of preterm birth to the burden of disease in children under five years in LMIC and to inform the planning of healthcare interventions to address this burden, a renewed understanding of the assessment and documentation of outcomes for babies born preterm is needed | Precheck Critically low |
| Goetschalckx, Elise (2020) | https://dx.doi.org/10.3390/ijerph17062144 | Glomerular Filtration Rate in Former Extreme Low Birth Weight Infants over the Full Pediatric Age Range: A Pooled Analysis | Review Design: Prognostic  Framework: PICOTS  P (Population): ELBW children; preterms as full population; ELBW (<1000gr)  I / Index / Condition: Gestational age  Comparator / Ref.: comparison with full term  Outcome(s): Other: kidney (GFR); school age (5-12 years); adolescence (13-18)  Timing: birth; throughout childhood  Setting: preterm birth as risk factors for impaired GFR | Precheck Critically low |
| Gogou, Maria (2019) | https://dx.doi.org/10.1007/s12519-019-00240-8 | Sleep and prematurity: sleep outcomes in preterm children and influencing factors | Review Design: Prognostic  Framework: PICOTS  P (Population): preterm infants; preterms as full population; "preterm" (<37 weeks)  I / Index / Condition: Gestational age; neonatal complication (BPD, IVH, PVL, NEC, Sepsis); socioeconomic factor  Comparator / Ref.: comparison with full term; Any other prognostic factor/model; children with and with no caffeine intake, preterm children with and without theophylline vs. full-term children, different temperature settings, phototherapy vs. no phototherapy  Outcome(s): Other: sleep; school age (5-12 years); adolescence (13-18)  Timing: birth; school-age, adolescence, young adulthood  Setting: The alterations in sleep patterns are an outcome of prematurity (immaturity of nervous system) as well as of  postnatal factors and comorbidities. Sleep problems in this population of infants seems to be a missing piece of the puzzle  of impaired neurodevelopment. | Precheck Critically low |
| Gotardo, Juliana Wendling (2019) | https://dx.doi.org/10.1371/journal.pone.0223427 | Impact of peri-intraventricular haemorrhage and periventricular leukomalacia in the neurodevelopment of preterms: A systematic review and meta-analysis | Review Design: Prognostic  Framework: PICOTS  P (Population): patients born under 37 weeks GA; preterms as full population; "preterm" (<37 weeks)  I / Index / Condition: neonatal complication (BPD, IVH, PVL, NEC, Sepsis)  Outcome(s): neurosensory/neurodevelopmental; motor-development (including CP, DCD); 12-36 month; preschool age (4-6 years); school age (5-12 years)  Timing: neonatal period; infancy and (early) childhood  Setting: There was no evidence supporting the hypothesis that PIVH causes impairment in neuropsychomotor development in our meta-analysis, but review of newer studies show an increased risk for lower intelligence scores in children with severe lesions, both PIVH and PVL. There is evidence to support the hypothesis that children with any degree of PIVH, especially those born below 1000 grams and those with severe haemorrhage, are at increased risk of developing CP, as well as children with PVL, both cystic and non-cystic. | Precheck Critically low |
| Gou, Xiaoyun (2018) | https://dx.doi.org/10.1136/bmjopen-2017-020735 | Association between bronchopulmonary dysplasia and cerebral palsy in children: a meta-analysis | Review Design: Prognostic  Framework: PICOTS  P (Population): children with bronchopulmonary dysplasia; preterms as full population; <28 weeks; 28 - <32 weeks; 32 - <37 weeks; VLBW (<1500gr)  I / Index / Condition: neonatal complication (BPD, IVH, PVL, NEC, Sepsis)  Outcome(s): motor-development (including CP, DCD); 12-36 month  Timing: during hospitalisation; age at which CP diagnosis can be confirmed; 2 years  Setting: The setting is primarily neonatal and pediatric care, where BPD is recognized as a significant risk factor for CP, thus requiring careful monitoring and management in preterm infants | ROBIS High |
| Guo, Bao-Qiang (2022) | https://dx.doi.org/10.1007/s00787-022-02078-4 | Prevalence of autism spectrum disorder diagnosis by birth weight, gestational age, and size for gestational age: a systematic review, meta-analysis, and meta-regression | Review Design: Prognostic  Framework: PICOTS  P (Population): not reported; preterms as subgroup; <28 weeks; 28 - <32 weeks; "preterm" (<37 weeks); VLBW (<1500gr); Other: low-birth weight (< 2500g), small/appropriate/large- for-gestational-age  I / Index / Condition: Gestational age; Other: birth weight  Comparator / Ref.: comparison with full term; Any other prognostic factor/model; subcategories : very-low-birth weight (<1500g), low-birth weight (< 2500g), normal-birth  weight (2500-4000g), high-birth weight (macroso- mia;>4000g), very-preterm birth (<32 weeks of ges- tation), preterm birth (<37 weeks of gestation),  term birth (≥37 and<42 weeks of gestation), postterm  birth (≥42 weeks of gestation), small/appropriate/large- for-gestational-age  Outcome(s): behaviour/mental health (include ADHD, autism); not reported  Timing: birth; not reported  Setting: The focus was on the prevalence of ASD in relation to birth weight, gestational age, and size for gestational age compared with the reference prevalence (those in normal-birth weight, term, and appropriate-for-gestational-age individuals), the prevalence estimates of ASD diag- nosis in very-low-birth weight, low-birth weight, very preterm, preterm, and small-for-gestational-age individuals increased  significantly. | Precheck Critically low |
| Gusnedi, Gusnedi (2023) | https://dx.doi.org/10.6133/apjcn.202306_32(2).0001 | Risk factors associated with childhood stunting in Indonesia: A systematic review and meta-analysis | Review Design: Prognostic  Framework: PICOTS  P (Population): children under five years in Indonesia; preterms as subgroup; "preterm" (<37 weeks)  I / Index / Condition: Gestational age; socioeconomic factor; other: LBW  Comparator / Ref.: comparison with full term  Outcome(s): growth/metabolism; preschool age (4-6 years)  Timing: birth; <5years  Setting: Preterm birth is associated with stunting in Indonesia | Precheck Critically low |
| Haddad, Summer (2019) | https://dx.doi.org/10.1016/j.midw.2019.01.009 | Sleep in parents of preterm infants: A systematic review | Review Design: Prognostic  Framework: PICOTS  P (Population): parents of preterm infants; Other: not applicable, for the participants were parents; Other: parents of preterm infants  I / Index / Condition: Other: being parents of preterm infants  Comparator / Ref.: Any other prognostic factor/model; not reported; compare sleep quantity and quality between mothers of preterm and term infants  Outcome(s): Other: Parental sleep; Other: during infant hospitalization and following discharge  Timing: birth; first year with the infant  Setting: The studies took place in various settings, including hospitals and homes. The role of prognostic factors like stress, anxiety, and depression in influencing sleep quantity and quality among parents of preterm infants was a key focusâ€‹. Quality and quantity of sleep among parents of preterm infants is inadequate and may negatively influence family health outcomes. | Precheck Critically low |
| Hortensius, Lisa M. (2018) | https://dx.doi.org/10.1542/peds.2018-0609 | Neurodevelopmental Consequences of Preterm Isolated Cerebellar Hemorrhage: A Systematic Review | Review Design: Prognostic  Framework: PICOTS  P (Population): preterm infants diagnosed with isolated cerebellar hemorrhage, specifically infants born before 32 weeks of gestation; preterms as full population; <28 weeks; 28 - <32 weeks  I / Index / Condition: neonatal complication (BPD, IVH, PVL, NEC, Sepsis)  Comparator / Ref.: Any other prognostic factor/model; NDO Versus Size of Cerebellar  Hemorrhage; NDO of Preterm Infants Without  Brain Injury  Outcome(s): behaviour/mental health (include ADHD, autism); cognition; neurosensory/neurodevelopmental; motor-development (including CP, DCD); language development; 12-36 month  Timing: neonatal period; a period extending to at least 12 months of age  Setting: More than one-third of all preterm infants born at <32 weeks GA with isolated cerebellar hemorrhage are severely impaired in the cognitive, motor, language, and behavioral domains. The incidence of severe impairment in ≥ 1 outcome domain is 43% to 75%. Involvement of the vermis and a large bleeding lead to the highest incidence of patients with severe impairment. | Precheck Critically low |
| Huang, Jichong (2016) | https://dx.doi.org/10.1371/journal.pone.0153655 | Prenatal, Perinatal and Neonatal Risk Factors for Intellectual Disability: A Systemic Review and Meta-Analysis | Review Design: Prognostic  Framework: PICOTS  P (Population): not reported; preterms as subgroup; "preterm" (<37 weeks)  I / Index / Condition: Gestational age  Comparator / Ref.: comparison with full term  Outcome(s): cognition; not reported  Timing: risk factor prematurity: birth; infancy through adulthood  Setting: Ten prenatal factors (advanced maternal age, maternal black race, low maternal education, third or more parity, maternal alcohol use, maternal tobacco use, maternal diabetes, maternal hypertension, maternal epilepsy and maternal asthma), one perinatal factor (preterm birth) and two neonatal factors (male sex and low birth weight) were significantly associated with increased risk of ID. | Precheck Critically low |
| Hussain, Sultana Monira (2018) | https://dx.doi.org/10.1186/s13075-018-1627-7 | Could low birth weight and preterm birth be associated with significant burden of hip osteoarthritis? A systematic review | Review Design: Prognostic  Framework: PICOTS  P (Population): preterm and LBW infants; preterms as full population; "preterm" (<37 weeks); Other: LBW  I / Index / Condition: Gestational age  Comparator / Ref.: comparison with full term  Outcome(s): Other: hip bone abnormality, hip bone osteoarthrosis; school age (5-12 years); adolescence (13-18)  Timing: birth; lifelong  Setting: lack of high-quality studies findings suggest that LBW and preterm birth are potential risk factors for hip bone shape abnormalities and hip OA requiring THA in adulthood.  current lack of effective treatment and preventive strategies for hip OA, this is an area where further research is needed to reduce the burden of hip OA (targeted monitoring and early interventions for “at-risk group”) | Precheck Critically low |
| Isayama, Tetsuya (2017) | https://dx.doi.org/10.1542/peds.2017-0266 | Health Services Use by Late Preterm and Term Infants From Infancy to Adulthood: A Meta-analysis | Review Design: Prognostic  Framework: PICOTS  P (Population): late-preterm infants; preterms as full population; Other: 34 to 36 weeks' gestation  I / Index / Condition: Gestational age  Comparator / Ref.: comparison with full term  Outcome(s): readmission to hospital; Other: health service utilization (HSU); 0-3 month; 0-12 month; 12-36 month; preschool age (4-6 years); school age (5-12 years); adolescence (13-18)  Timing: birth; all ages up to 18  Setting: Late-preterm infants had higher rates of various cause-specific HSU than term  infants for jaundice, infection, respiratory problems, asthma, and neurologic and/or mental  health problems during certain periods, including adulthood | Precheck Critically low |
| Jacobsen, Pernille E. (2014) | https://dx.doi.org/10.1111/eos.12094 | Developmental enamel defects in children born preterm: a systematic review | Review Design: Prognostic  Framework: PICOTS  P (Population): children born at a gestational age before 37 wk; preterms as full population; "preterm" (<37 weeks)  I / Index / Condition: Gestational age; Other: birth weight  Comparator / Ref.: comparison with full term; Any other prognostic factor/model; various levels of prematurity or birth weight  Outcome(s): teeth; school age (5-12 years); adolescence (13-18); Other: 3 yr of age because the primary dentition is expected to be fully erupted in children at this age  Timing: birth; primary and secondary teeth  Setting: The aim of the study was to show the association between prematurity (and related factors like low birth weight) and developmental enamel defects in children. The role of these prognostic factors is to identify the likelihood of dental health issues in children born preterm or with low birth weight | Precheck Critically low |
| James, Evlyn (2018) | https://dx.doi.org/10.1186/s12887-017-0976-8 | Preterm birth and the timing of puberty: a systematic review | Review Design: Prognostic  Framework: PICOTS  P (Population): pubertal and post-pubertal adolescents and adults; preterms as full population; "preterm" (<37 weeks)  I / Index / Condition: Gestational age  Comparator / Ref.: comparison with full term  Outcome(s): Other: timing of puberty; school age (5-12 years); adolescence (13-18); Other: onset of puberty  Timing: birth; onset of puberty  Setting: The published evidence does not suggest that being born preterm in itself leads to a significant acceleration in the onset of puberty. This lack of evidence for a substantial effect should prove reassuring for public health purposes, and clinicians counseling parents of infants born preterm | Precheck Critically low |
| Jongbloed-Pereboom, Marjolein (2012) | https://dx.doi.org/10.1016/j.neubiorev.2012.02.005 | Motor learning and working memory in children born preterm: a systematic review | Review Design: Prognostic  Framework: PICOTS  P (Population): Preterm-born children; preterms as full population; "preterm" (<37 weeks)  I / Index / Condition: Gestational age  Comparator / Ref.: comparison with full term  Outcome(s): cognition; motor-development (including CP, DCD); 0-3 month; 0-12 month; 12-36 month; preschool age (4-6 years); school age (5-12 years); adolescence (13-18)  Timing: birth; 0-18 years  Setting: preterm birth affected performance on visual working memory tests. Information regarding motor learning and the role of working memory on the different components of motor learn- ing was not available. | Precheck Critically low |
| Kaempf, Joseph W. (2023) | https://dx.doi.org/10.1136/archdischild-2022-324457 | Change in neurodevelopmental outcomes for extremely premature infants over time: a systematic review and meta-analysis | Review Design: Prognostic  Framework: PICOTS  P (Population): infants born <27  weeks gestation or <1000 g birth weight; preterms as full population; <28 weeks; ELBW (<1000gr)  I / Index / Condition: Gestational age; Other: ELBW  Comparator / Ref.: Any other prognostic factor/model; different cohorts from the same institutions  Outcome(s): neurosensory/neurodevelopmental; 12-36 month  Timing: birth; 18-36 months corrected age  Setting: Higher survival rates unaccompanied by improvement in  neurodevelopment highlight urgency for renewed focus  on the causes of NDI and evidence-based strategies to  reduce brain injury. | Precheck Critically low |
| Kenmoe, Sebastien (2020) | https://dx.doi.org/10.1371/journal.pone.0229357 | Comparison of health care resource utilization among preterm and term infants hospitalized with Human Respiratory Syncytial Virus infections: A systematic review and meta-analysis of retrospective cohort studies | Review Design: Prognostic  Framework: PICOTS  P (Population): preterm infants hospitalized for HRSV infections; preterms as full population; 28 - <32 weeks; "preterm" (<37 weeks)  I / Index / Condition: Gestational age  Comparator / Ref.: comparison with full term  Outcome(s): Lung; infections (RSV, ...); readmission to hospital; not reported  Timing: birth; childhood  Setting: The document suggests that pediatricians, program managers, and policymakers should be aware of the high risk for increased use of medical resources and poor outcomes in early and late PT hospitalized for HRSV infections compared to FT children. Special attention should be paid to PT infants under 2 years, especially in resource-limited areas such as Africa and Southeast Asia, where there is virtually no access to or policies for the administration of HRSV prophylaxis. | Precheck Critically low |
| Kerr-Wilson, C. O. (2012) | https://dx.doi.org/10.1093/pubmed/fdr024 | Meta-analysis of the association between preterm delivery and intelligence | Review Design: Prognostic  Framework: PICOTS  P (Population): Preterm born children; preterms as full population; "preterm" (<37 weeks)  I / Index / Condition: Gestational age  Comparator / Ref.: comparison with full term; Any other prognostic factor/model; comparison of different stages of prematurity to determine whether there is a dose-response relationship  Outcome(s): cognition; preschool age (4-6 years); school age (5-12 years); adolescence (13-18)  Timing: birth; school age  Setting: There is a strong and consistent body of evidence suggesting an association between preterm del ivery and reduced IQ, with evidence of a dose-response relationship with gestational age. | Precheck Critically low |
| King, Brian C. (2021) | https://dx.doi.org/10.1016/j.semperi.2021.151394 | The financial burden on families of infants requiring neonatal intensive care | Review Design: Prognostic  Framework: PICOTS  P (Population): infants requiring NICU care at birth; preterm as subgroup; <28 weeks; 28 - <32 weeks; 32 - <37 weeks; VLBW (<1500gr); ELBW (<1000gr)  I / Index / Condition: Gestational age; Other: NICU care at birth  Outcome(s): Other: financial impact on families; 0-12 month; 12-36 month; preschool age (4-6 years); school age (5-12 years); adolescence (13-18)  Timing: birth; up to a lifetime  Setting: impact of these costs on the care of preterms | Precheck Critically low |
| Knuf, Markus (2023) | https://dx.doi.org/10.1080/21645515.2023.2191575 | Penta- and hexavalent vaccination of extremely and very-to-moderate preterm infants born at less than 34 weeks and/or under 1500 g: A systematic literature review | Review Design: Prognostic  Framework: PICOTS  P (Population): infants born at < 1500g and/or preterm infants born at < 34 weeks; preterms as subgroup; Other: <34 GA, born at < 1500g  I / Index / Condition: Gestational age  Comparator / Ref.: comparison with full term  Outcome(s): infections (RSV, ...); readmission to hospital; 0-3 month; 0-12 month; 12-36 month  Timing: birth; Childhood  Setting: Data from the 14 studies included in the review showed that the immunogenicity and the safety profile of penta- and hexavalent vaccines in preterm infants was generally similar to those seen in full-term infants, with the exception of an increase in cardiorespiratory adverse  events such as apnea, bradycardia and desaturation following vaccination in preterm infants. Despite recommendations of vaccinating preterm infants according to their actual age, and the relatively high  completion rate of the primary immunization schedule, vaccination was often delayed, increasing the  vulnerability of this high-risk population to vaccine-preventable diseases. | Precheck Critically low |
| Kong, Annice H. T. (2018) | https://dx.doi.org/10.1016/j.earlhumdev.2018.09.015 | Background EEG features and prediction of cognitive outcomes in very preterm infants: A systematic review | Review Design: Prognostic  Framework: PICOTS  P (Population): very preterm infants (born ≤ 34 weeks GA);; preterms as full population; Other: preterms born ≤ 34 weeks  I / Index / Condition: Other: EEG features  Comparator / Ref.: Any other prognostic factor/model; EEG description  Outcome(s): cognition; neurosensory/neurodevelopmental; Other: Assessments were carried out at a wide range of times from 4 months to 9 years of age in most cases using an age-appropriate neurodevelopment scale.  Timing: time point of prognostication is age of eeg (from birth till term equivalent age),; not reported  Setting: Although more evidence from further research is needed to draw definitive conclusions, some background EEG features are useful in predicting cognitive outcomes. In particular, a key finding of this meta-analysis was that, across four studies, pooled specificity of the ‘Dysmature/disorganised pattern’ for predicting cognitive outcome was high. This suggests that this abnormal EEG pattern will be useful to inform future outcome prognoses for infants born very preterm. | Precheck Critically low |
| Kotecha, Sarah J. (2022) | https://dx.doi.org/10.1001/jamapediatrics.2022.1990 | Geographical Differences and Temporal Improvements in Forced Expiratory Volume in 1 Second of Preterm-Born Children: A Systematic Review and Meta-analysis | Review Design: Prognostic  Framework: PICOTS  P (Population): preterm-born children, both with and without bronchopulmonary dysplasia (BPD); preterms as full population; "preterm" (<37 weeks)  I / Index / Condition: Gestational age; neonatal complication (BPD, IVH, PVL, NEC, Sepsis)  Comparator / Ref.: comparison with full term; Any other prognostic factor/model;   - All preterm-born participants (including those with and without BPD). - Preterm-born participants without BPD. - All-BPD group (including those with BPD28 and BPD36). - BPD28. - BPD36.  year of birth, age at time of lung function, surfactant usage, and geographical region of birth or residence (region)  Outcome(s): Lung; 12-36 month; preschool age (4-6 years); school age (5-12 years); adolescence (13-18); Other: 3 to 52 years  Timing: birth; 3 to 52 years  Setting: deficits in %FEV1 for preterm-born participants when compared with term-born participants. FEV1 is associated not only with lung health but also with cardiovascular outcomes and all-cause mortality. Decrements were larger for the preterm-born participants who had BPD. Improvements in %FEV1 were noted over time, especially for the BPD groups, possibly due to the introduction of surfactant and improvement in early-life therapies.  Differences were also observed for %FEV1 when geographical areas were compared with Scandinavian countries.  These results emphasize the importance of being aware of the potential deficits when treating preterm-born survivors and of finding suitable treatments | Precheck Critically low |
| Kotecha, Sarah J. (2013) | https://dx.doi.org/10.1136/thoraxjnl-2012-203079 | Effect of preterm birth on later FEV1: a systematic review and meta-analysis |  |  |
| Lacalle, Laura (2023) | https://dx.doi.org/10.3389/fpsyg.2023.1216825 | Intelligence Quotient (IQ) in school-aged preterm infants: A systematic review | Review Design: Prognostic  Framework: PICOTS  P (Population): Preterm children (<37 weeks); preterms as full population; "preterm" (<37 weeks)  I / Index / Condition: Gestational age  Comparator / Ref.: comparison with full term  Outcome(s): cognition; school age (5-12 years)  Timing: birth; school age (6-12 years)  Setting: The results confirm an association between preterm birth and intelligence. The analyzed studies show that preterm children abtained worse IQ results than their full-term peers in school age. | Precheck Critically low |
| Laccetta, Gianluigi (2022) | https://dx.doi.org/10.1177/19714009221102454 | 1H-magnetic resonance spectroscopy and its role in predicting neurodevelopmental impairment in preterm neonates: A systematic review | Review Design: Prognostic  Framework: PICOTS  P (Population): newborns with gestational age at birth <37 weeks which underwent at least one H-MRS scan within 52 weeksâ€™ postmenstrual age and neurodevelopmental assessment within 4 years of age; preterms as full population; preterm (<37 weeks)  I / Index / Condition: Other: MRI features  Comparator / Ref.: not reported  Outcome(s): cognition; neurosensory/neurodevelopmental; motor-development (including CP, DCD); language development; preschool age (4-6 years)  Timing: in two studies out of 4, patients underwent H-MRS scan at two different time points: around 32 weeksâ€™ and 40 weeksâ€™ postmenstrual age. In the studies by Hyodo et al. and Hart et al. patients underwent only H-MRS scan at term-equivalent age.; not reported  Setting: Our systematic review suggests that metabolite ratios at H-MRS could be considered promising prognostic biomarkers of neurodevelopmental impairment. | Precheck Critically low |
| Larsson, Johanna (2022) | https://dx.doi.org/10.3390/children9111695 | The Role of Family Function and Triadic Interaction on Preterm Child Development-A Systematic Review | Review Design: Prognostic  Framework: PICOTS  P (Population): premature infants (gestational age < 37 weeks) and/or infants with low birth weight (<2500 g); preterms as full population; "preterm" (<37 weeks); Other: low birth weight <2500 g  I / Index / Condition: Gestational age  Comparator / Ref.: comparison with full term  Outcome(s): behaviour/mental health (include ADHD, autism); cognition; neurosensory/neurodevelopmental; motor-development (including CP, DCD); Parent-Child-Interaction; 0-3 month; 0-12 month; 12-36 month; preschool age (4-6 years); school age (5-12 years)  Timing: birth; infancy to late childhood  Setting: Quality of family interactions is either equal to or poorer in families with preterm children, compared with families with full-term children. Importantly, the link between quality of family interactions and child development outcome is stronger in preterm children compared with full-term children, regarding both positive and negative influence. Our results highlight the importance of strengthening family interactions in order to promote development in preterm children. | Precheck Critically low |
| Lee, Deborah Mei Xuan (2023) | https://dx.doi.org/10.1007/s11136-022-03311-y | Quality of life of patients and caregivers affected by bronchopulmonary dysplasia: a systematic review | Review Design: Prognostic  Framework: PICOTS  P (Population): caregivers with preterm babies diagnosed with bronchopulmonary dysplasia (BPD) or children/adults who were born premature and diagnosed with BPD; preterms as full population; "preterm" (<37 weeks)  I / Index / Condition: neonatal complication (BPD, IVH, PVL, NEC, Sepsis)  Comparator / Ref.: comparison with full term; Any other prognostic factor/model; preterm without BPD  Outcome(s): Quality of Life; 0-12 month; 12-36 month; school age (5-12 years); adolescence (13-18)  Timing: neonatal period; childhood and adolescence  Setting: The intended setting is a clinical environment, focusing on the impact of BPD on patients' and caregivers' quality of life.The QoL of patients differed by domainsâ€”some were poorer or similar, but none of the QoL domains was better than QoL of healthy controls.  increasing illness acuity negatively affected the QoL of BPD patients. The QoL of BPD patients and their caregivers was  most adversely affected during the immediate post-discharge period and tended to improve with time. The physical QoL of  BPD patients was similar to that of preterm babies without BPD when assessed during late childhood and early adulthood. | Precheck Critically low |
| Li, S. (2014) | https://dx.doi.org/10.1111/obr.12214 | Preterm birth and risk of type 1 and type 2 diabetes: systematic review and meta-analysis | Review Design: Prognostic  Framework: PICOTS  P (Population): preterms; preterms as full population; "preterm" (<37 weeks)  I / Index / Condition: Gestational age  Outcome(s): Other: type 1 or type 2 diabetes; school age (5-12 years); adolescence (13-18); Other: under 15, under 16, under 6, under 19, ...  Timing: birth; childhood and adulthood  Setting: The results suggested that preterm birth was a significant and inde- pendent risk factor for both type 1 and type 2 diabetes. | Precheck Critically low |
| Li, Wanling (2019) | https://dx.doi.org/10.1016/j.yebeh.2019.05.016 | Do premature and postterm birth increase the risk of epilepsy? An updated meta-analysis | Review Design: Prognostic  Framework: PICOTS  P (Population): premature or postterm birth; preterms as subgroup; "preterm" (<37 weeks)  I / Index / Condition: Gestational age  Comparator / Ref.: comparison with full term; Any other prognostic factor/model; postterm  Outcome(s): Epilepsy; 0-3 month; 0-12 month; 12-36 month; preschool age (4-6 years); school age (5-12 years); adolescence (13-18)  Timing: birth; childhood and adulthood  Setting: preterm birth is strongly associated with an increased risk of epilepsy throughout childhood and persisting into adulthood, and the association becomes stronger as GA decreased | Precheck Critically low |
| Liao, Lihong (2020) | https://dx.doi.org/10.3389/fped.2020.00405 | Association of Low Birth Weight and Premature Birth With the Risk of Metabolic Syndrome: A Meta-Analysis | Review Design: Prognostic  Framework: PICOTS  P (Population): general population; preterms as subgroup; "preterm" (<37 weeks)  I / Index / Condition: Gestational age  Comparator / Ref.: comparison with full term  Outcome(s): growth/metabolism; school age (5-12 years); adolescence (13-18); Other: adults  Timing: birth; across childhood and adulthood  Setting: Low birth weight and preterm might be risk factors for metabolic syndrome | Precheck Critically low |
| Lima, Laura Jordana Santos (2021) | https://dx.doi.org/10.1007/s00784-021-04146-6 | Prenatal, perinatal and postnatal events associated with hypomineralized second primary molar: a systematic review with meta-analysis | Review Design: Prognostic  Framework: PICOTS  P (Population): children with hypomineralized second primary molar; preterms as subgroup; not reported  I / Index / Condition: Gestational age  Outcome(s): teeth; Other: hypomineralized second primary molar; 0-3 month; 0-12 month; 12-36 month; preschool age (4-6 years); school age (5-12 years)  Timing: birth; childhood  Setting: The role of these prognostic factors is to assess their association with the likelihood of developing HSPM. Maternal smoking, maternal hypertension, low birth weight, prematurity, delivery complications, need for incu- bation, not breastfeeding, antibiotic use, fever and childhood asthma were associated with HSPM. | Precheck Critically low |
| Linsell, Louise (2015) | https://dx.doi.org/10.1001/jamapediatrics.2015.2175 | Prognostic Factors for Poor Cognitive Development in Children Born Very Preterm or With Very Low Birth Weight: A Systematic Review | Review Design: Prognostic  Framework: PICOTS  P (Population): children born very preterm (VPT) at 32 weeks or less or with very low birth weight (VLBW) of 1250 g or less; preterms as full population; <28 weeks; 28 - <32 weeks; Other: very low birth weight (VLBW) of 1250 g or less  I / Index / Condition: Gestational age; neonatal complication (BPD, IVH, PVL, NEC, Sepsis); socioeconomic factor; Other: sex  Comparator / Ref.: not reported  Outcome(s): cognition; neurosensory/neurodevelopmental; language development; Other: after 18 months  Timing: birth; after 18 months  Setting: The setting and role of prognostic factors were to identify factors that predict poor cognitive development in children born VPT or with VLBW. There was evidence that male sex, nonwhite race/ethnicity, lower level of parental  education, and lower birth weight were predictive of global cognitive impairment in children  younger than 5 years. In older children, only the influence of parental education was sustained.  Male sex was also predictive of language impairment in early infancy, but not in middle childhood.  Gestational age was a poor predictor of cognitive outcome, probably because of a reduced  discriminatory power in cohorts restricted to a narrow gestational age range. | Precheck Critically low |
| Linsell, Louise (2016) | https://dx.doi.org/10.1097/DBP.0000000000000238 | Prognostic Factors for Behavioral Problems and Psychiatric Disorders in Children Born Very Preterm or Very Low Birth Weight: A Systematic Review | Review Design: Prognostic  Framework: PICOTS  P (Population): children born at ≤ 32 weeks gestational age or with birth weight ≤ 1250 g; preterms as full population; <28 weeks; 28 - <32 weeks; Other: birth weight ≤ 1250 g  I / Index / Condition: Gestational age; neonatal complication (BPD, IVH, PVL, NEC, Sepsis); socioeconomic factor  Outcome(s): behaviour/mental health (include ADHD, autism); neurosensory/neurodevelopmental; 12-36 month; preschool age (4-6 years); school age (5-12 years)  Timing: birth; after 18 months of age  Setting: The intended setting is clinical, focusing on predicting long-term behavioral and psychiatric outcomes in children born very preterm or with very low birth weight. This review has identified the need for further  research examining the etiology of disorders of psychological development in the VPT/VLBW population to refine risk prediction and identify targets for intervention. | Precheck Critically low |
| Linsell, Louise (2016) | https://dx.doi.org/10.1111/dmcn.12972 | Prognostic factors for cerebral palsy and motor impairment in children born very preterm or very low birthweight: a systematic review | Review Design: Prognostic  Framework: PICOTS  P (Population): born after 1 January 1990;  <= 32 weeks or birthweight <= 1250g  not a highly select group; preterms as full population; <28 weeks; 28 - <32 weeks; Other: birthweight of 1250g or less  I / Index / Condition: Gestational age; neonatal complication (BPD, IVH, PVL, NEC, Sepsis); Other: sex  Outcome(s): motor-development (including CP, DCD); Other: after 18 months of age  Timing: birth/neonatal; after 18 months of age  Setting: here was strong evidence that intraventricular haemorrhage and periventricular  leukomalacia, and some evidence that the use of postnatal steroids and non-use of antenatal  steroids, were prognostic factors for CP. Male sex and gestational age were of limited use as  prognostic factors for CP in cohorts restricted to ≤ 32 weeks gestation; however, in children older  than 5 years with no major disability, there was evidence that male sex was a predictive factor for  motor impairment. | Precheck Critically low |
| Ma, Defu (2021) | https://dx.doi.org/10.1038/s41430-020-00831-z | Effects of rapid growth on fasting insulin and insulin resistance: a system review and meta-analysis | Review Design: Prognostic  Framework: PICOTS  P (Population): not reported; preterms as subgroup; not reported  I / Index / Condition: Other: rapid growth  Comparator / Ref.: not reported  Outcome(s): growth/metabolism; Other: fasting blood insulin concentration and HOMA-IR; Other: follow up age 1-11years (one study 20y)  Timing: up to 6.6 years of age; childhood and adolescence  Setting: This meta-analysis suggested that rapid growth would result in high insulin and HOMA-IR, especially for full-term infants. However, rapid growth is relatively harmless for subjects who are <6 years old, low birth weight or SGA, and is even protective for preterm subjects. | Precheck Critically low |
| Maconachie, Gail D. E. (2013) | https://dx.doi.org/10.1001/jamaophthalmol.2013.4001 | Risk factors and genetics in common comitant strabismus: a systematic review of the literature | Review Design: Prognostic  Framework: PICOTS  P (Population): children with strabismus; preterms as subgroup; "preterm" (<37 weeks)  I / Index / Condition: Gestational age; Other: birth weight  Comparator / Ref.: Any other prognostic factor/model; different birth weights (e.g., 1000 g to 1500 g vs. more than 1500 g) and different gestational ages (e.g., 33-36 weeks vs. other ranges)  Outcome(s): eyes; 0-12 month; 12-36 month; preschool age (4-6 years); school age (5-12 years); adolescence (13-18)  Timing: birth; infancy through adolescence  Setting: VLBW as a risk factor for strabismus | Precheck Critically low |
| Magalhaes, Rafael C. (2018) | https://dx.doi.org/10.1016/j.ijdevneu.2017.10.006 | Inflammatory molecules and neurotrophic factors as biomarkers of neuropsychomotor development in preterm neonates: A Systematic Review | Review Design: Prognostic  Framework: PICOTS  P (Population): preterm neonates at all gestational ages; preterms as full population; "preterm" (<37 weeks)  I / Index / Condition: Other: inflammatory molecules and neurotrophic factors  Outcome(s): cognition; neurosensory/neurodevelopmental; motor-development (including CP, DCD); 12-36 month; preschool age (4-6 years)  Timing: birth; infancy and childhood  Setting: The intended setting and role of the prognostic factors were to assess the relationship between inflammatory molecules and neurotrophic factors and neuropsychomotor development in preterm neonates. This is a clinical or research setting where these biomarkers could be used to understand and predict developmental outcomes in this population. There is preliminary evidence indicating that circulating inflammatory molecules are associated with motor and cognitive development in preterm neonates, even considering different populations. | Precheck Critically low |
| Martin-Calvo, Nerea (2022) | https://dx.doi.org/10.1111/obr.13380 | Low birth weight and small for gestational age are associated with complications of childhood and adolescence obesity: Systematic review and meta-analysis | Review Design: Prognostic  Framework: PICOTS  P (Population): LBW or SGA; preterms as subgroup; Other: children with low birth weight (LBW) or small for gestational age (SGA)  I / Index / Condition: Other: low birth weight (LBW) or small for gestational age (SGA)  Comparator / Ref.: Any other prognostic factor/model; SGA, AGA  Outcome(s): Other: type 2 diabetes; 12-36 month; preschool age (4-6 years); school age (5-12 years); adolescence (13-18)  Timing: birth; up to adulthood  Setting: Compared with children or adolescents born with adequate size for gestational age, those SGA had 2.33-fold higher risk of T2D (95% confidence interval [CI]: 1.05â€“5.17). Furthermore, LBW and being SGA were associ- ated with 0.20 higher mean homeostasis model assessment of insulin resistance (HOMA-IR) values (95% CI: 0.02-0.38). Given the high prevalence of preterm babies, from a population perspective, these results may be of great importance as they point to the existence of a potentially vulnerable subgroup of children and adoles- cents that could benefit from screening tests and early preventive strategies. | Precheck Critically low |
| Martinez-Nadal, Silvia (2020) | https://dx.doi.org/10.3390/ijerph18010074 | Cognitive and Learning Outcomes in Late Preterm Infants at School Age: A Systematic Review | Review Design: Prognostic  Framework: PICOTS  P (Population): infants at 3 years of age or beyond born as late preterms (34-36 weeks); preterms as full population; "preterm" (<37 weeks); Other: Late Preterm Infants  I / Index / Condition: Gestational age  Comparator / Ref.: comparison with full term  Outcome(s): cognition; Education; preschool age (4-6 years); school age (5-12 years)  Timing: birth; preschool age to school age  Setting: need to implement screening strategies to facilitate early risk detection and minimize the negative effects of this morbidity in childhood | Precheck Critically low |
| Marzola, Enrica (2021) | https://dx.doi.org/10.1007/s00737-020-01057-5 | The role of prenatal and perinatal factors in eating disorders: a systematic review | Review Design: Prognostic  Framework: PICOTS  P (Population): with eating disorder; preterms as subgroup; <28 weeks; 28 - <32 weeks; Other: SGA  I / Index / Condition: Gestational age; Other: SGA  Comparator / Ref.: comparison with full term  Outcome(s): behaviour/mental health (include ADHD, autism); Other: eating disorders: anorexia nervosa (AN) and bulimia nervosa (BN); not reported  Timing: birth; not reported  Setting: The factors that showed a more robust association with AN were higher maternal age, preeclampsia and eclampsia, multiparity, hypoxic complications, prematurity, or being born preterm (< 32 weeks) and small for gestational age or lower birth size. BN was only associated with maternal stress during pregnancy. | Precheck Critically low |
| Mathewson, Karen J. (2017) | https://dx.doi.org/10.1037/bul0000091 | Mental health of extremely low birth weight survivors: A systematic review and meta-analysis | Review Design: Prognostic  Framework: PICOTS  P (Population): participants with a mean age >=5 years  born extremly preterm or ELBW; preterms as full population; ELBW (<1000gr)  I / Index / Condition: Gestational age; socioeconomic factor; Other: ELBW  Comparator / Ref.: comparison with full term  Outcome(s): behaviour/mental health (include ADHD, autism); Peer relation/social interaction; Other: >= 5 years  Timing: birth; childhood, adolescence, adulthood  Setting: Children born at ELBW were reported by parents and teachers to be at significantly greater risk than NBW controls for inattention and hyperactivity, internalizing, and externalizing symptoms. ELBW children were also at greater risk for conduct and oppositional disorders, autistic symptoms, and social difficulties. Risks for parent-reported inattention and hyperactivity, internalizing, and social problems were greater in adolescents born at ELBW. In contrast, ELBW teens self-reported lower inattention, hyperactivity, and oppositional behavior levels than their NBW peers. Depression, anxiety, and social difficulties were elevated in ELBW survivors in adulthood. | Precheck Critically low |
| McBryde, Melinda (2020) | https://dx.doi.org/10.1001/jamanetworkopen.2020.2027 | Academic Outcomes of School-Aged Children Born Preterm: A Systematic Review and Meta-analysis | Review Design: Prognostic  Framework: PICOTS  P (Population): children of school age with preterm birth (5 to 18 years and born during or after 1980); preterms as full population; "preterm" (<37 weeks); Other: LBW  I / Index / Condition: Gestational age  Comparator / Ref.: comparison with full term  Outcome(s): Education; school age (5-12 years); adolescence (13-18)  Timing: birth; school age  Setting: This information could be used by healthcare professionals, educators, and parents to provide early interventions or additional educational support to preterm-born children who are at risk of academic difficulties. | Precheck Critically low |
| McGowan, Jennifer E. (2011) | https://dx.doi.org/10.1542/peds.2010-2257 | Early childhood development of late-preterm infants: a systematic review | Review Design: Prognostic  Framework: PICOTS  P (Population): Late-Preterm Infants; preterms as full population; Other: 34 to 36 weeksâ€™ gestation  I / Index / Condition: Gestational age  Comparator / Ref.: comparison with full term; Any other prognostic factor/model; very preterm  Outcome(s): growth/metabolism; behaviour/mental health (include ADHD, autism); cognition; neurosensory/neurodevelopmental; motor-development (including CP, DCD); language development; Education; 12-36 month; preschool age (4-6 years); school age (5-12 years)  Timing: birth; 1-7 years  Setting: Evidence suggests that LPIs are at increased risk of adverse developmental outcomes and academic difficulties up to 7 years of age in comparison to term infants | Precheck Critically low |
| Moore, Gregory P. (2013) | https://dx.doi.org/10.1001/jamapediatrics.2013.2395 | Neurodevelopmental outcomes at 4 to 8 years of children born at 22 to 25 weeks' gestational age: a meta-analysis | Review Design: Prognostic  Framework: PICOTS  P (Population): preterm infant survivors born between 22 and 25 weeks gestational age; preterms as full population; <28 weeks  I / Index / Condition: Gestational age  Comparator / Ref.: comparison with full term  Outcome(s): neurosensory/neurodevelopmental; preschool age (4-6 years); school age (5-12 years)  Timing: birth; 4 to 8 years  Setting: The study was set in a clinical context, aiming to understand the neurodevelopmental outcomes of extremely preterm infants based on their gestational age at birth. All extremely preterm infant survivors have a substantial likelihood of developing moderate to severe impairment. There was a statistically significant absolute decrease in moderate to severe impairment between each week of gestation. | Precheck Critically low |
| Moreira, Rafaela S. (2014) | https://dx.doi.org/10.1016/j.jped.2013.05.010 | Effect of preterm birth on motor development, behavior, and school performance of school-age children: a systematic review | Review Design: Prognostic  Framework: PICOTS  P (Population): preterm infants; preterms as full population; <28 weeks; 28 - <32 weeks; 32 - <37 weeks  I / Index / Condition: Gestational age; Other: birth weight, classification of birth weight in relation to gestational age  Comparator / Ref.: comparison with full term  Outcome(s): behaviour/mental health (include ADHD, autism); motor-development (including CP, DCD); Education; school age (5-12 years)  Timing: birth; school age  Setting: preterm as a risk factor regarding problems in behavior, school performance, motor performance at (pre-)school age | Precheck Critically low |
| Mukerji, Amit (2015) | https://dx.doi.org/10.1542/peds.2015-0944 | Periventricular/Intraventricular Hemorrhage and Neurodevelopmental Outcomes: A Meta-analysis | Review Design: Prognostic  Framework: PICOTS  P (Population): preterm infants (under 34 weeks completed gestational age); preterms as full population; Other: under 34 weeks completed gestational age  I / Index / Condition: neonatal complication (BPD, IVH, PVL, NEC, Sepsis)  Comparator / Ref.: Any other prognostic factor/model; no PIVH, mild PIVH, severe PIVH  Outcome(s): cognition; neurosensory/neurodevelopmental; post-discharge death; 12-36 month; preschool age (4-6 years); school age (5-12 years); adolescence (13-18)  Timing: birth; (early) childhood through adolescence  Setting: Increasing grades of PIVH may be associated with adverse long-term neurodevelopmental outcomes, and  mild PIVH alone may independently have an impact compared with no PIVH.  Severe PIVH is associated with a worse outcome compared with both mild PIVH and no PIVH. Neonatologists, follow-up clinicians, parents, and teachers must be cautious in their assessment of infants and children with a history of mild PIVH because these children may need additional resources to maximize their potential. | Precheck Critically low |
| Murray, E. (2015) | https://dx.doi.org/10.1111/1471-0528.13435 | Differential effect of intrauterine growth restriction on childhood neurodevelopment: a systematic review | Review Design: Prognostic  Framework: PICOTS  P (Population): children with IUGR; preterms as subgroup; not reported  I / Index / Condition: Gestational age; Other: intrauterine growth restriction (IUGR)  Comparator / Ref.: Any other prognostic factor/model; non-IUGR children, IUGR with FCR with IUGR without FCR  Outcome(s): behaviour/mental health (include ADHD, autism); cognition; neurosensory/neurodevelopmental; motor-development (including CP, DCD); language development; Other: sleep; 0-3 month; 0-12 month; 12-36 month; preschool age (4-6 years); school age (5-12 years)  Timing: perinatal; up to 12 years of age  Setting: To assess the impact of IUGR on various neurodevelopmental domains. IUGR increases the risk of neurodevelopmental impairment during childhood differentially across domains. IUGR children born preterm or with evidence of fetal circulatory redistribution are more severely affected. | Precheck Critically low |
| Murray, Sarah R. (2017) | https://dx.doi.org/10.12688/wellcomeopenres.12783.1 | Long term cognitive outcomes of early term (37-38 weeks) and late preterm (34-36 weeks) births: A systematic review | Review Design: Prognostic  Framework: PICOTS  P (Population): children born at late preterm (34-36 weeks) and early term (37-38 weeks) births; preterms as subgroup; Other: late preterm (34-36 weeks)  I / Index / Condition: Gestational age  Comparator / Ref.: comparison with full term  Outcome(s): cognition; 12-36 month; preschool age (4-6 years); school age (5-12 years); adolescence (13-18)  Timing: birth; childhood to adolescence  Setting: The role of the prognostic factor (gestational age) is to evaluate its impact on long-term cognitive outcomes in these children.  Children born at 39-41 weeks have higher cognitive outcome scores compared to those born at early term (37-38 weeks). For children born late preterm, the data is scarce and when compared to full term (37-42 weeks) did not show any difference in IQ scores. | Precheck Critically low |
| Myrhaug, Hilde Tinderholt (2019) | https://dx.doi.org/10.1542/peds.2018-0933 | Survival and Impairment of Extremely Premature Infants: A Meta-analysis | Review Design: Prognostic  Framework: PICOTS  P (Population): preterms born at 22-27 weeks GA; preterms as full population; <28 weeks  I / Index / Condition: Gestational age  Comparator / Ref.: Any other prognostic factor/model; comparison of different gestational ages  Outcome(s): neurosensory/neurodevelopmental; 12-36 month  Timing: birth; between 18 to 36 months of age  Setting: The study aimed to provide a comprehensive summary of survival rates and risks of impairment for extremely premature infants in high-income countries. The prognostic factors were used to assess the likelihood of survival and neurodevelopmental outcomes in this specific population group. Survival without impairment was substantially lower for children born at ,25 weeks GA than for those born later. | Precheck Critically low |
| Nalbandyan, Marine (2021) | https://dx.doi.org/10.1002/bdr2.1904 | Nongenetic risk factors for infantile cataracts: Systematic review of observational studies | Review Design: Prognostic  Framework: PICOTS  P (Population): children with infantile cataracts; preterms as subgroup; "preterm" (<37 weeks); VLBW (<1500gr)  I / Index / Condition: Gestational age; Other: birth weight  Comparator / Ref.: not reported  Outcome(s): eyes; not reported  Timing: birth; childhood  Setting: preterm birth and VLBW as a risk factor for infantile cataracts | Precheck Critically low |
| Neel, M. L. M. (2018) | https://dx.doi.org/10.1111/cch.12561 | Parenting style impacts cognitive and behavioural outcomes of former preterm infants: A systematic review | Review Design: Prognostic  Framework: PICOTS  P (Population): children were assessed at ages that ranged from 0-18 years,  although all examined at least some child outcomes at less than two years per our inclusion criteria.; preterms as full population; "preterm" (<37 weeks)  I / Index / Condition: Other: parenting style  Outcome(s): behaviour/mental health (include ADHD, autism); cognition; Other: <7 years  Timing: <2 years; childhood  Setting: This systematic review examines eight axes of parenting style and their associations with cognitive and behavioral outcomes of former preterm children in early childhood. For behavioral measures, strong evidence indicates that parenting styles incorporating responsivity and warmth are associated with improved outcomes, while rejection is associated with worse outcomes and autonomy support and coercion do not appear to have any association with positive or negative outcomes. For cognitive measures, the only strong evidence favors parental responsivity. Weaker evidence supports positive associations between parental demandingness, structure and autonomy support and improved child outcomes. | Precheck Critically low |
| Nicholls, Alice (2016) | 10.1080/10833196.2016.1250032 | Can the Prechtl method for the qualitative assessment of general movements be used to predict neurodevelopmental outcome, at eighteen months to three years, of infants born preterm? | Review Design: Prognostic  Framework: PICOTS  P (Population): participants born before 37 weeks gestational age; preterms as full population; "preterm" (<37 weeks)  I / Index / Condition: Other: GMA  Outcome(s): cognition; neurosensory/neurodevelopmental; motor-development (including CP, DCD); 12-36 month  Timing: term-20 weeks corrected age; not reported  Setting: In conclusion, evidence suggests that the Prechtl method for the qualitative assessment of general movements, during the writhing and fidgety period, can be used to predict the neurodevelopmental outcome in the motor domain, at eighteen months to three years, in infants born preterm.  The Prechtl assessment was found to be more predictive  of severe neurological impairments compared to minor  neurological impairments. | Precheck Critically low |
| Occhi-Alexandre, Ingrid Gomes Perez (2020) | https://dx.doi.org/10.1111/ipd.12610 | Prevalence of dental caries in preschool children born preterm and/or with low birth weight: A systematic review with meta-analysis of prevalence data | Review Design: Prognostic  Framework: PICOTS  P (Population): preschool children in the primary dentition phase, specifically those born preterm and/or with low birth weight (LBW); preterms as subgroup; "preterm" (<37 weeks)  I / Index / Condition: Gestational age  Comparator / Ref.: comparison with full term  Outcome(s): teeth; preschool age (4-6 years)  Timing: birth; primary dentition phase (preschool age)  Setting: association between preterm birth and dental caries | Precheck Critically low |
| Ou-Yang, Mei-Chen (2020) | https://dx.doi.org/10.1371/journal.pone.0232238 | Accelerated weight gain, prematurity, and the risk of childhood obesity: A meta-analysis and systematic review | Review Design: Prognostic  Framework: PICOTS  P (Population): preterm infants; preterms as full population; "preterm" (<37 weeks)  I / Index / Condition: Gestational age; Other: small/appropriate for gestational age  Comparator / Ref.: comparison with full term; Any other prognostic factor/model; preterm-SGA  Outcome(s): growth/metabolism; preschool age (4-6 years); school age (5-12 years); adolescence (13-18)  Timing: birth; Childhood and adolescence  Setting: In conclusion, accelerated weight gain at infancy among preterm children may be a critical contributor to obesity in later life. Establishing optimal growth trajectories and timely referral to health care providers may be of clinical importance | Precheck Critically low |
| Paes, Bosco (2016) | nan | Defining the Risk and Associated Morbidity and Mortality of Severe Respiratory Syncytial Virus Infection Among Infants with Chronic Lung Disease | Review Design: Prognostic  Framework: PICOTS  P (Population): children with CLD / BPD with RSV infection; preterms as subgroup; <28 weeks; 28 - <32 weeks; 32 - <37 weeks; ELBW (<1000gr)  I / Index / Condition: Gestational age; neonatal complication (BPD, IVH, PVL, NEC, Sepsis)  Comparator / Ref.: Any other prognostic factor/model; children without BPD/CLD, other high risk populations  Outcome(s): infections (RSV, ...); post-discharge death; readmission to hospital; Other: healthcare resource utilization (ICU admission, oxygen therapy, mechanical ventilation); 0-3 month; 0-12 month; 12-36 month  Timing: time point of RSV infection; duration of RSV infection  Setting: Severe RSV infection in infants and young children with CLD/BPD poses a significant health burden in Western countries. Further studies focussing on the burden of  RSV infection in this well-recognized population at high risk for severe disease are needed to help improve outcomes and plan allocation of healthcare resources. | Precheck Critically low |
| Paquette, Katryn (2019) | https://dx.doi.org/10.1371/journal.pone.0210366 | Cancer risk in children and young adults born preterm: A systematic review and meta-analysis | Review Design: Prognostic  Framework: PICOTS  P (Population): preterms; preterms as full population; "preterm" (<37 weeks)  I / Index / Condition: Gestational age  Comparator / Ref.: comparison with full term  Outcome(s): Other: incidence of childhood malignancy; Other: age at diagnosis 28 days - <19years  Timing: birth; infancy into (young) adulthood  Setting: The intended setting is observational, where the prognostic factor (preterm birth) is used to predict the likelihood of developing cancer. | Precheck Critically low |
| Pascal, Aurelie (2018) | https://dx.doi.org/10.1111/dmcn.13675 | Neurodevelopmental outcome in very preterm and very-low-birthweight infants born over the past decade: a meta-analytic review | Review Design: Prognostic  Framework: PICOTS  P (Population): VPT or VLBW infants w; preterms as full population; <28 weeks; 28 - <32 weeks; VLBW (<1500gr)  I / Index / Condition: Gestational age  Comparator / Ref.: not reported  Outcome(s): cognition; neurosensory/neurodevelopmental; motor-development (including CP, DCD); 0-12 month; 12-36 month; preschool age (4-6 years)  Timing: birth; up to 6 years  Setting: Even though neonatal intensive care has improved over recent decades, there is still a wide range of neurodevelopmental disabilities resulting from VPT and VLBW births. However, pooled prevalences of CP have diminished over the years. | Precheck Critically low |
| Paulsen, Megan E. (2021) | https://dx.doi.org/10.1159/000517951 | Long-Term Outcomes after Early Neonatal Hyperglycemia in VLBW Infants: A Systematic Review | Review Design: Prognostic  Framework: PICOTS  P (Population): VLBW (Very Low Birth Weight) infants who were either less than 32 weeks gestational age or weighed less than 1500 grams.; preterms as full population; <28 weeks; 28 - <32 weeks; VLBW (<1500gr)  I / Index / Condition: Other: early neonatal hyperglycemia (blood glucose concentration >150 mg/dL or 8.3 mmol/L during the first 28 days of life)  Comparator / Ref.: Any other prognostic factor/model; normoglycemic/standard glycemic control/non-insulin treatment groups.  Outcome(s): blood pressure/cardiovascular; growth/metabolism; neurosensory/neurodevelopmental; motor-development (including CP, DCD); Other: at least 1 post-NICU follow-up (long-term outcomes from 4-month corrected GA to 7 years old)  Timing: during the first 28 days of life; from 4-month corrected gestational age to 7 years old  Setting: The study aimed to understand the long-term effects of early hyperglycemia on VLBW infants after discharge from the neonatal intensive care unit. Some studies found differences in growth, metabolic  health, and neurodevelopment outcomes between VLBW  preterm infants with hyperglycemia and without hyperglycemia, while other studies found no differences between  groups. The overall graded quality of evidence was low. | Precheck Critically low |
| Perez Tarazona, S. (2018) | https://dx.doi.org/10.1016/j.aller.2017.02.004 | Bronchopulmonary dysplasia as a risk factor for asthma in school children and adolescents: A systematic review | Review Design: Prognostic  Framework: PICOTS  P (Population): patients with a diagnosis of bronchopulmonary dysplasia (BPD) in preterm infants (PTIs) with a gestational age of under 32 weeks and/or with a weight at birth of less than 1500 g born between 1977 and 2005; preterms as full population; 28 - <32 weeks; VLBW (<1500gr)  I / Index / Condition: Gestational age; neonatal complication (BPD, IVH, PVL, NEC, Sepsis)  Outcome(s): Lung; preschool age (4-6 years); school age (5-12 years); adolescence (13-18)  Timing: perinatal (BPD diagnosis); childhood and adolescence  Setting: It cannot be argued that BPD, as an independent factor of prematurity, increases the risk of asthma defined by clinical parameters in school-children and adolescents.  The most frequently used criterion for defining BPD was the need for oxygen (O2) with a corrected GA of 36 weeks. | Precheck Critically low |
| Petrou, Stavros (2020) | https://dx.doi.org/10.1007/s40273-019-00865-7 | Preference-Based Health-Related Quality of Life Outcomes Associated with Preterm Birth: A Systematic Review and Meta-analysis | Review Design: Prognostic  Framework: PICOTS  P (Population): born preterm (<37 weeksâ€™ gestation) or low birth-weight (<2500 g), or their parents, carers or siblings; preterms as subgroup; preterm (<37 weeks); other: LBW  I / Index / Condition: Gestational age  Comparator / Ref.: comparison with full term  Outcome(s): Quality of Life; preschool age (4-6 years); school age (5-12 years); adolescence (13-18)  Timing: birth; childhood and adolescence  Setting: All studies  reporting health utility values for individuals born preterm or at low birthweight and a control group of individuals born at  full term or normal birthweight reported lower utility values in the study groups, regardless of age at assessment, respond- ent type or valuation method. | Precheck Critically low |
| Radaelli, Graciane (2023) | https://dx.doi.org/10.1055/s-0042-1758866 | Motor and cognitive outcomes of neonates with low birth weight in Brazil: a systematic review and meta-analysis | Review Design: Prognostic  Framework: PICOTS  P (Population): low birth weight (LBW), including preterm neonates as defined by the WHO: LBW (< 2500 g), very LBW (< 1500 g), and extremely LBW (< 1000 g); preterms as subgroup; VLBW (<1500gr); ELBW (<1000gr); Other: LBW (< 2500 g)  I / Index / Condition: Gestational age; Other: birth weight (BW)  Comparator / Ref.: comparison with full term  Outcome(s): cognition; neurosensory/neurodevelopmental; motor-development (including CP, DCD); 0-3 month; 0-12 month; 12-36 month; preschool age (4-6 years); school age (5-12 years)  Timing: birth; infancy and childhood  Setting: The intended setting of the prognostic factors is to understand the impact of low birth weight and gestational age on the cognitive and motor development of children in Brazil. The role of these factors is to assess how they influence the long-term neurodevelopmental outcomes in this population. The results of the present study reinforce that impaired motor and cognitive functions can be a significant long-term outcome of LBW. The lower the gestational age at delivery, the higher the risk of impairment in those domains. | Precheck Critically low |
| Ramaswamy, Viraraghavan Vadakkencherry (2021) | https://dx.doi.org/10.1371/journal.pone.0255352 | ELBW and ELGAN outcomes in developing nations-Systematic review and meta-analysis | Review Design: Prognostic  Framework: PICOTS  P (Population): ELGANs (born at less than 28 weeks of gestation) and / or ELBW neonates (birth weight of less than 1000 grams) from a LMIC; preterms as full population; <28 weeks; ELBW (<1000gr)  I / Index / Condition: Gestational age; socioeconomic factor; Other: birth weight  Comparator / Ref.: N/A; Any other prognostic factor/model; for primary outcome (survival until discharge)  three income levels: low income (LI), lower middle-income (LMI) and upper middle-income (UMI) survival between two time periods: epoch 1: 2000-2009 and epoch 2: 2010-2019 geographical regions neonates with varying baseline sickness.  Outcome(s): neurosensory/neurodevelopmental; 12-36 month  Timing: birth; at 24 months of age  Setting: The intended setting is Low and Middle-Income Countries (LMICs), focusing on the morbidity and mortality among ELBW and ELGAN. Mortality and morbidity amongst ELBW and ELGANs is still a significant burden in LMICs. | Precheck Critically low |
| Rath, Chandra Prakash (2021) | https://dx.doi.org/10.1136/archdischild-2019-318207 | Diffuse excessive high signal intensity on term equivalent MRI does not predict disability: a systematic review and meta-analysis | Review Design: Prognostic  Framework: PICOTS  P (Population): preterm infants <37 weeks; preterms as full population; "preterm" (<37 weeks)  I / Index / Condition: Other: diffuse excessive high signal intensity (DEHSI) on term equivalent age MRI (at 37 to 42 weeks with a maximum range of 3 weeks earlier or 4 months later)  Outcome(s): neurosensory/neurodevelopmental; Other: follow up ≥ 1 year of corrected age (18months - 13 years)  Timing: at term equivalent age, around 37 to 42 weeks of gestational age; ≥ 1year corrected age  Setting: The study was set in a clinical context, evaluating the role of DEHSI on TEA-MRI as a prognostic tool for predicting long-term developmental outcomes in preterm infants. | Precheck Critically low |
| Rees, Philippa (2022) | https://dx.doi.org/10.1542/peds.2022-057442 | Preterm Brain Injury and Neurodevelopmental Outcomes: A Meta-analysis | Review Design: Prognostic  Framework: PICOTS  P (Population): preterm children born at <37 weeks gestation with a diagnosis of intracranial hemorrhage or white matter injury; preterms as full population; "preterm" (<37 weeks)  I / Index / Condition: Gestational age; neonatal complication (BPD, IVH, PVL, NEC, Sepsis); Other: white matter injury (WMI)  Comparator / Ref.: Any other prognostic factor/model; preterm infants without brain injury  Outcome(s): behaviour/mental health (include ADHD, autism); cognition; neurosensory/neurodevelopmental; motor-development (including CP, DCD); language development; 0-3 month; 0-12 month; 12-36 month  Timing: neonatal period; up to 3 y of age  Setting: The study is set in a clinical context, with the prognostic factors (IVH and WMI) being used to predict neurodevelopmental outcomes in preterm infants. The focus is on understanding how these brain injuries in preterm infants impact their development up to the age of 3 years. Mild IVH, severe IVH, and WMI are associated with adverse neurodevelopmental outcomes. | Precheck Critically low |
| Rees, Philippa (2023) | https://dx.doi.org/10.1136/bmjpo-2022-001810 | School-age outcomes of children after perinatal brain injury: a systematic review and meta-analysis | Review Design: Prognostic  Framework: PICOTS  P (Population): Children with a diagnosis of brain injury occurring at or around the  time of birth (including during the neonatal period) as defined by the  DHSC (including those with any white matter injury but not including  those with isolated seizures) infants with moderate to severe HIE born in  the post-therapeutic hypothermia era (ie, where infants received  therapeutic hypothermia); preterms as subgroup; not reported  I / Index / Condition: Gestational age; neonatal complication (BPD, IVH, PVL, NEC, Sepsis)  Comparator / Ref.: Any other prognostic factor/model; children without perinatal brain injury  Outcome(s): Epilepsy; behaviour/mental health (include ADHD, autism); cognition; neurosensory/neurodevelopmental; motor-development (including CP, DCD); language development; Education; preschool age (4-6 years); school age (5-12 years); adolescence (13-18)  Timing: neonatal period; between 5 and 18 years of age  Setting: The intended setting and role of the prognostic factors are to assess and predict the long-term neurodevelopmental outcomes of children who have suffered perinatal brain injuries. The study aims to provide insights into the impacts of these injuries during the critical school-age period. Studies reported an increased risk of persisting neurodevelopmental impairment at school age after neonatal meningitis. Cognitive impairment and special educational needs were highlighted after moderate-to-severe hypoxic-ischaemic encephalopathy. However, there were limited comparative studies providing school-aged outcome data across neurodevelopmental domains and few rovided adjusted data. Findings were further limited  by the heterogeneity of studies. | Precheck Critically low |
| Rees, Philippa (2023) | https://dx.doi.org/10.1111/dmcn.15713 | Childhood outcomes after low-grade intraventricular haemorrhage: A systematic review and meta-analysis | Review Design: Prognostic  Framework: PICOTS  P (Population): school-age children (between 5â€“18 years) born preterm at less than 37weeks' gestation or with a birthweight of less than 2500 g  after low-grade intraventricular haemorrhage (IVH) - Infants; preterms as full population; "preterm" (<37 weeks); Other: birthweight of less than  2500 g  I / Index / Condition: neonatal complication (BPD, IVH, PVL, NEC, Sepsis); Other: IVH grades 1 and 2  Comparator / Ref.: Any other prognostic factor/model; preterm without IVH  Outcome(s): cognition; neurosensory/neurodevelopmental; motor-development (including CP, DCD); language development; Education; preschool age (4-6 years); school age (5-12 years); adolescence (13-18)  Timing: neonatal; school-age  Setting: provides further evidence that low-grade IVH is unlikely to be a benign pathology (as previously thought) by highlighting that it has a measurable impact on children's cognitive and motor development at school  findings, including the outstanding gaps in our knowledge, should inform counselling of parents of infants born preterm with low-grade IVH on the neonatal unit. importance of ongoing quality improvement initiatives to prevent IVH amongst infants born preterm on the neonatal unit in addition to reiterating the importance of neurodevelopmental follow-up and support for this population. | Precheck Critically low |
| Ritchie, Kirsten (2015) | https://dx.doi.org/10.1111/dmcn.12783 | Social development of children born very preterm: a systematic review | Review Design: Prognostic  Framework: PICOTS  P (Population): sample included children born VPT (gestation ≤ 33wks and/or birthweight ≤ 1500g), children were born after 1990, children aged 0 to 17 years; preterms as full population; Other: <33 wks  I / Index / Condition: Gestational age  Comparator / Ref.: comparison with full term  Outcome(s): Peer relation/social interaction; 0-12 month; 12-36 month; preschool age (4-6 years); school age (5-12 years); adolescence (13-18)  Timing: birth; childhood and adolescence (0-17 years)  Setting: Children born VPT have poorer social competence. These difficulties emerge early and persist throughout childhood. | Precheck Critically low |
| Rocha, Nelci Adriana Cicuto Ferreira (2020) | https://dx.doi.org/10.1177/1367493519864742 | Impact of mother-infant interaction on development during the first year of life: A systematic review | Review Design: Prognostic  Framework: PICOTS  P (Population): child participants who were less than 12 months of age; not reported; not reported  I / Index / Condition: Other: quality of motherâ€“infant interaction  Outcome(s): Peer relation/social interaction; 0-12 month  Timing: birth; first year of life  Setting: Prematurity, infant age, multiples births, maternal anxiety, maternal opioid exposure, history of foster care, and criminal record were the only factors found to mediate the relationship between motherâ€“infant interaction and social, cognitive, and language development. The quality of the interactions between a mother and infant can both positively and negatively influence cognitive, language, and social outcomes during the first year of life. | Precheck Critically low |
| Sabri, Mohammad Reza (2021) | https://dx.doi.org/10.4103/jrms.JRMS_869_20 | The associations of low birth weight with primary hypertension in later life: A systematic review and meta-analysis | Review Design: Prognostic  Framework: PICOTS  P (Population): children with LBW mean age â‰¤18 years; preterms as subgroup; Other: LBW  I / Index / Condition: Gestational age; Other: low birth weight  Comparator / Ref.: Any other prognostic factor/model; comparison with NBW, subgroup analysis of preâ€‘ and fullâ€‘term babies  Outcome(s): blood pressure/cardiovascular; Other: â‰¤18 years  Timing: birth; <=18years  Setting: The review aims to understand the association between low birth weight and the development of essential hypertension in children. "Although findings of the correlation between BW and EHTN have conflicted." Subgroup analysis was performed on the pre- and full-term babies. there is a difference between full- and pre-term groups according to systolic/diastolic blood pressure. | Precheck Critically low |
| Sandoval, Carolina Cruvinel (2022) | https://dx.doi.org/10.1080/21622965.2021.1915145 | The impact of preterm birth on the executive functioning of preschool children: A systematic review | Review Design: Prognostic  Framework: PICOTS  P (Population): children born preterm (<37 weeks of GA) at the preschool age (2-6 years old; preterms as full population; "preterm" (<37 weeks)  I / Index / Condition: Gestational age  Comparator / Ref.: comparison with full term  Outcome(s): Other: executive functioning; 12-36 month; preschool age (4-6 years)  Timing: birth; preschool age  Setting: The study is set in a research context, examining the impact of prematurity on the executive functioning of preschool-aged children. The role of the prognostic factor (prematurity) is to assess its influence on the development of executive functioning in this age group.  In comparison to children born full-term, preschool children born preterm exhibit executive functioning deficits in the dimensions of the global index, inhibitory control, cognitive flexibility, working memory, and plan- ning/executive functioning. These findings are independent of the degree of prematurity at birth. Since executive functioning has many complex components, future studies should assess the dimensions of executive functioning separately in preschool-aged children born preterm, rather than as a single measure. | Precheck Critically low |
| Sangla, Ananya (2021) | https://dx.doi.org/10.1136/bmjopen-2020-047770 | Effects of prematurity on long-term renal health: a systematic review | Review Design: Prognostic  Framework: PICOTS  P (Population): preterm infants; preterms as full population; "preterm" (<37 weeks)  I / Index / Condition: Gestational age  Comparator / Ref.: comparison with full term  Outcome(s): blood pressure/cardiovascular; Other: kidney function, CKD; 0-12 month; 12-36 month; preschool age (4-6 years); school age (5-12 years); adolescence (13-18)  Timing: birth; up to adulthood  Setting: The intended setting is clinical, focusing on the long-term renal health of individuals born preterm. Prematurity is likely linked to increased  risk of kidney dysfunction and high BP in childhood and  into early adulthood. Premature birth conferred a twofold  increased risk of CKD and extremely premature birth  conferred a threefold increased risk of CKD. | Precheck Critically low |
| Schappin, Renske (2013) | https://dx.doi.org/10.1371/journal.pone.0054992 | Rethinking stress in parents of preterm infants: a meta-analysis | Review Design: Prognostic  Framework: PICOTS  P (Population): parents of preterm infants (<37 weeks and/or <2500 g) admitted to the NICU,; preterms as full population; "preterm" (<37 weeks)  I / Index / Condition: Gestational age; Other: LBW  Comparator / Ref.: comparison with full term; Any other prognostic factor/model; no-treatment control-group parents of term infants (>37 weeks and/or >2500 g);  mother vs. father age of child, related factors (sex, GA and birth weight, birth year, maternal age, education)  Outcome(s): Other: parental stress levels; Other: not applicable  Timing: birth; not reported  Setting: The results indicate that parents of preterm-born children experience only slightly more stress than parents of term-born children, with small effect sizes. Furthermore, mothers have slightly more stress than fathers, but these effect sizes are also small. Parents report more stress for infants with lower gestational ages and lower birth weights. There is a strong effect for infant birth year, with decreasing parental stress from the 1980s onward, probably due to increased quality of care for preterm infants. | Precheck Critically low |
| Serati, Marta (2017) | https://dx.doi.org/10.1111/jcpp.12779 | Research Review: The role of obstetric and neonatal complications in childhood attention deficit and hyperactivity disorder - a systematic review | Review Design: Prognostic  Framework: PICOTS  P (Population): birth up until 12 years of age; preterms as subgroup; "preterm" (<37 weeks); Other: LBW, VLBW, ELBW, very preterm, extreme preterm  I / Index / Condition: Gestational age; Other: birth weight  Comparator / Ref.: comparison with full term  Outcome(s): behaviour/mental health (include ADHD, autism); Other: <12years  Timing: birth; infancy and childhood  Setting: The intended setting for these prognostic factors included clinical and research settings. The role of these factors was to evaluate the risk and development of ADHD in children based on preterm births and associated neonatal complicationsâ€‹â€‹. Among perinatal complications, available data indicate low birth weight (LBW) (Cohen’s d effect size range: 0.31-1.64-small effect size) and preterm birth (PB) (range d: 0.41â€“0.68) as the most important factors associated with a future diagnosis of ADHD. PB and LBW children should be carefully monitored for an early diagnosis of ADHD limiting the impact of the disease in life span. | Precheck Critically low |
| Shi, Linan (2020) | https://dx.doi.org/10.1042/BSR20200870 | Relationship between preterm, low birth weight and early childhood caries: a meta-analysis of the case-control and cross-sectional study | Review Design: Prognostic  Framework: PICOTS  P (Population): subjects were younger than or equal to 6 years old + premature or LBW; preterms as subgroup; "preterm" (<37 weeks); Other: LBW  I / Index / Condition: Gestational age  Comparator / Ref.: comparison with full term  Outcome(s): teeth; Other: <= 6 years  Timing: birth; <= 6 years  Setting: This meta-analysis indicated that preterm increased the risk of ECC significantly; however, LBW was not a risk factor for ECC. | Precheck Critically low |
| Shi, Ting (2015) | https://dx.doi.org/10.7189/jogh.05.020416 | Risk factors for respiratory syncytial virus associated with acute lower respiratory infection in children under five years: Systematic review and meta-analysis | Review Design: Prognostic  Framework: PICOTS  P (Population): children with a diagnosis of ALRI and laboratory confirmed RSV illnes <5years; preterms as subgroup; "preterm" (<37 weeks); Other: LBW, Gestational age <33 weeks  I / Index / Condition: Gestational age  Outcome(s): infections (RSV, ...); Other: <5years  Timing: birth; younger than 5 y  Setting: We identified 20 studies (3 were unpublished data) with  “good quality” that investigated 18 risk factors for RSV-associated ALRI in children younger than five years old. Among them, 8 risk  factors were significantly associated with RSV-associated ALRI.  The meta-estimates of their odds ratio (ORs) with corresponding  95% confidence intervals (CI) are prematurity 1.96 (95% CI 1.44- 2.67), low birth weight 1.91 (95% CI 1.45-2.53), ... | Precheck Critically low |
| Siqueira, Fernando C. M. (2019) | https://dx.doi.org/10.1016/j.msksp.2018.10.001 | Are perinatal factors associated with musculoskeletal pain across the lifespan? A systematic review with meta-analysis | Review Design: Prognostic  Framework: PICOTS  P (Population): preterm individuals; preterms as subgroup; "preterm" (<37 weeks)  I / Index / Condition: Gestational age  Comparator / Ref.: not reported  Outcome(s): Other: musculoskeletal pain; school age (5-12 years); adolescence (13-18); Other: up to 45years  Timing: birth; from childhood to adulthood  Setting: Musculoskeletal pain is associated with a complex interaction between diverse risk factors Also related to those comorbidities are perinatal factors, which in turn may impact on muscu- loskeletal pain. | Precheck Critically low |
| Slattery, Justine (2012) | https://dx.doi.org/10.1111/j.1469-8749.2012.04318.x | Early sucking and swallowing problems as predictors of neurodevelopmental outcome in children with neonatal brain injury: a systematic review | Review Design: Prognostic  Framework: PICOTS  P (Population): infants diagnosed with peri-, pre-, or early postnatal brain injury and â„or infants born very preterm (<32wks) with very low birthweight (<1500g), at risk of neonatal brain injury;; preterms as subgroup; <28 weeks; 28 - <32 weeks; VLBW (<1500gr)  I / Index / Condition: Other: early sucking and swallowing problems  Outcome(s): neurosensory/neurodevelopmental; 0-12 month; 12-36 month  Timing: perinatal; infancy and early childhood  Setting: currently insufficient evidence to clearly determine the relation between early sucking and swallowing problems and neonatal brain injury, may predict later neurodevelopment outcome. early sucking and swallowing problems do affect a consistent proportion of infants (35â€“48%) with varied aetiologies of neonatal brain injury and that perinatal comorbidities, in particular respiratory disease, increase the likelihood of early sucking and swallowing difficulties in these infant | Precheck Critically low |
| Somhovd, Mikael J. (2012) | https://dx.doi.org/10.1111/j.1469-8749.2012.04407.x | Anxiety in adolescents born preterm or with very low birthweight: a meta-analysis of case-control studies | Review Design: Prognostic  Framework: PICOTS  P (Population): adolescents aged between 11 and 20 years. These adolescents were either born very preterm (<32 weeks of gestation) or with very low birthweight (VLBW; <1500g); preterms as full population; <28 weeks; 28 - <32 weeks; VLBW (<1500gr)  I / Index / Condition: Gestational age  Comparator / Ref.: comparison with full term  Outcome(s): behaviour/mental health (include ADHD, autism); school age (5-12 years); adolescence (13-18)  Timing: birth; adolescents older than 11 years  Setting: children with VLBWâ„VPB have a higher incidence of clinically significant anxiety levels. The mechanisms are likely to involve biological vulnerability factors as well as environmental factors known to increase anxiety. Hence, care should be taken to meet the immediate needs of the child and parents in the dramatic context of preterm birth as well as their long-term needs; distinction between studies on children and adolescents in this context is important as adolescence is a crucial period when considering developmental psychopathology | Precheck Critically low |
| Stipdonk, Lottie W. (2016) | https://dx.doi.org/10.1111/dmcn.13151 | Auditory brainstem maturation in normal-hearing infants born preterm: a meta-analysis | Review Design: Prognostic  Framework: PICOTS  P (Population): normal-hearing preterm; preterms as full population; "preterm" (<37 weeks)  I / Index / Condition: Gestational age  Comparator / Ref.: comparison with full term  Outcome(s): ear-nose-throat; Other: at term age  Timing: birth; at term age  Setting: The intended setting is clinical, focusing on understanding the impact of prematurity on auditory brainstem maturation. The prognostic factors intended to assess the differences in auditory brainstem maturation between normal-hearing preterm and term-born infants. | Precheck Critically low |
| Stipdonk, Lottie W. (2018) | https://dx.doi.org/10.1371/journal.pone.0196607 | Language outcome related to brain structures in school-aged preterm children: A systematic review | Review Design: Prognostic  Framework: PICOTS  P (Population): school-aged preterm children (6-17 years); preterms as full population; "preterm" (<37 weeks)  I / Index / Condition: Gestational age  Comparator / Ref.: comparison with full term; Any other prognostic factor/model; brain volume or fractional anisotropy of a brain  (structurestructural MRI (T1- and T2-weighted sequences) or DTI)  Outcome(s): language development; Other: 6-17 years  Timing: birth; 6-17 years  Setting: The relations between oral language, verbal fluency and/or written language and MRI/DTI measurements of white mat- ter, gray matter, cerebellum, corpus callosum and/or the fasciculi are presented. Oral lan- guage skills and verbal fluency appear to be related to the corpus callosum. Oral language skills are also related to the uncinate fasciculus. There seems to be no clear relation between cerebellar development and verbal fluency skills. Not one single brain area is responsible for atypical language development, but several brain areas and their connec- tions are essential. | Precheck Critically low |
| Stipdonk, Lottie W. (2018) | https://dx.doi.org/10.1371/journal.pone.0203298 | Correction: Language outcome related to brain structures in school-aged preterm children: A systematic review |  |  |
| Su, Yingying (2021) | https://dx.doi.org/10.1111/jcpp.13358 | Research Review: Developmental origins of depression - a systematic review and meta-analysis | Review Design: Prognostic  Framework: PICOTS  P (Population): individuals with a clear diagnosis for major depression or depressive symptoms; preterms as subgroup; "preterm" (<37 weeks); Other: under 26 wGA  I / Index / Condition: Gestational age  Outcome(s): behaviour/mental health (include ADHD, autism); Other: major depression across the life span  Timing: birth; major depression across the life span  Setting: The meta-analysis found 12 prenatal, perinatal, and postnatal characteristics were associated with an increased risk of depression in offspring: low birth weight, premature birth, small gestational age, maternal education, socioeconomic status, having younger parents (<20 years), having older parents (≥ 35 years), maternal smoking, paternal smoking, maternal stress, maternal anxiety, and prenatal depression. | Precheck Critically low |
| Sun, Tong (2023) | https://dx.doi.org/10.1007/s12519-023-00701-1 | Risk of asthma in preterm infants with bronchopulmonary dysplasia: a systematic review and meta-analysis | Review Design: Prognostic  Framework: PICOTS  P (Population): preterm infants with bronchopulmonary dysplasia (BPD); preterms as full population; "preterm" (<37 weeks)  I / Index / Condition: Gestational age; neonatal complication (BPD, IVH, PVL, NEC, Sepsis)  Comparator / Ref.: Any other prognostic factor/model; preterms without BPD  Outcome(s): Lung; 12-36 month; preschool age (4-6 years); school age (5-12 years); adolescence (13-18)  Timing: birth; pre-adulthood  Setting: preterm infants with BPD have a much higher risk of developing asthma | Precheck Critically low |
| Taine, Marion (2018) | https://dx.doi.org/10.1111/ppe.12468 | Early postnatal growth and neurodevelopment in children born moderately preterm or small for gestational age at term: A systematic review | Review Design: Prognostic  Framework: PICOTS  P (Population): children born moderately preterm or small for gestational age at term; preterms as subgroup; Other: 32-36 weeks gestational age, birthweight between 1500 and 2500 g, SGA at term  I / Index / Condition: Other: early postnatal growth (EPG)  Outcome(s): cognition; neurosensory/neurodevelopmental; 0-12 month; 12-36 month; preschool age (4-6 years); school age (5-12 years); adolescence (13-18)  Timing: before age 3 years; infancy to adulthood  Setting: Overall, EPG was positively associated with neurodevelopmental outcome, especially Intelligence Quotient (IQ) when available. In this relationship, the first 6 months of life might be a critical period. Analysis of the few articles investigating the shape of the relationships revealed a non-linear association, with a plateau for IQ with higher weight gain, which suggests a possible ceiling effect further studies are needed to better explore the shape of the relationship (linear or non-linear), critical time windows and consider the perinatal context and early feeding mode Given its potential role, HCâ€™s growth deserves to be more systematically assessed in relation with later neurodevelopment. | Precheck Critically low |
| Taylor, Greig D. (2017) | https://dx.doi.org/10.1038/sj.ebd.6401219 | Molar incisor hypomineralisation | Review Design: Prognostic  Framework: PICOTS  P (Population): children with molar incisor hypomineralization; preterms as subgroup; "preterm" (<37 weeks)  I / Index / Condition: Gestational age  Outcome(s): teeth; preschool age (4-6 years); school age (5-12 years)  Timing: birth; when permanent molar erupt  Setting: There was little evidence of an association between MIH and perinatal factors such as prematurity, low birthweight, cesarean delivery and birth complications | Precheck Critically low |
| Telles, Fernando (2020) | https://dx.doi.org/10.1542/peds.2020-0146 | Changes in the Preterm Heart From Birth to Young Adulthood: A Meta-analysis | Review Design: Prognostic  Framework: PICOTS  P (Population): preterm individuals born at <37 weeks; preterms as full population; "preterm" (<37 weeks)  I / Index / Condition: Gestational age  Comparator / Ref.: comparison with full term  Outcome(s): blood pressure/cardiovascular; 0-3 month; 0-12 month; 12-36 month; preschool age (4-6 years); school age (5-12 years); adolescence (13-18)  Timing: birth; birth to young adulthood  Setting: The study was conducted in a research setting, aiming to determine the long-term impact of preterm birth on cardiac remodeling and function. The role of the prognostic factor (preterm birth) was to assess its effect on cardiac health across different developmental stages up to young adulthood. ONS: Preterm-born individuals have morphologic and functional cardiac impairments across developmental stages. These changes may make the preterm heart more vulnerable to secondary insults, potentially underlying their increased risk of early heart failure. | Precheck Critically low |
| Tinnion, Robert (2014) | https://dx.doi.org/10.1136/archdischild-2013-304615 | Preterm birth and subsequent insulin sensitivity: a systematic review | Review Design: Prognostic  Framework: PICOTS  P (Population): preterm infants; preterms as full population; "preterm" (<37 weeks); VLBW (<1500gr)  I / Index / Condition: Gestational age  Comparator / Ref.: comparison with full term  Outcome(s): growth/metabolism; Other: Insulin sensitivity; 12-36 month; preschool age (4-6 years); school age (5-12 years); adolescence (13-18)  Timing: birth; (pre)school age, adolescence  Setting: In infancy and early childhood there is a measurable association between IS and preterm birth. In later childhood and adulthood the strength of this association reduces, and current body composition becomes the variable most strongly associated with IS. | Precheck Critically low |
| Tunon-Dominguez, Isabel (2022) | https://dx.doi.org/10.3389/fped.2022.883218 | The influence between gestational age and postural control, a systematic review | Review Design: Prognostic  Framework: PICOTS  P (Population): children born preterm (<37 gestational weeks) without diseases and neurological sequela; preterms as full population; "preterm" (<37 weeks)  I / Index / Condition: Gestational age  Comparator / Ref.: comparison with full term  Outcome(s): motor-development (including CP, DCD); Other: Table1: 1month - 10 years (one study 17years)  Timing: birth; infancy, childhood and adolescence  Setting: All the studies found show a poorer postural control by the group of children born preterm compared to the group of children born at term and one study indicating more limited postural control with higher prematurity. | Precheck Critically low |
| Twilhaar, E. Sabrina (2018) | https://dx.doi.org/10.1136/archdischild-2017-312916 | Academic performance of children born preterm: a meta-analysis and meta-regression | Review Design: Prognostic  Framework: PICOTS  P (Population): preterm children; preterms as full population; "preterm" (<37 weeks)  I / Index / Condition: Gestational age; neonatal complication (BPD, IVH, PVL, NEC, Sepsis)  Outcome(s): cognition; school age (5-12 years)  Timing: birth; school age  Setting: Results are therefore applicable to the current  population of preterm children. It should be noted that the  included studies mostly comprised cohorts of extremely and  very preterm children. These results may be less applicable to  moderately/late preterm children.28 Academic performance is  a pre-eminent measure of outcome, given its strong relation  with important life outcomes.5 6 Despite influential advances  in neonatal healthcare, preterm children show considerable  academic difficulties. Given the increasing number of preterm  children and the substantial individual, social and economic  consequences of academic difficulties in these children,7 29 there  is a need to develop strategies that will improve outcomes after  preterm birth. Less than 10 studies  reported the incidence of sepsis, meningitis, NEC, postnatal  corticosteroids use and maternal education level. The role of  these factors could therefore not be assessed. | Precheck Critically low |
| Twilhaar, E. Sabrina (2018) | https://dx.doi.org/10.1001/jamapediatrics.2017.5323 | Cognitive Outcomes of Children Born Extremely or Very Preterm Since the 1990s and Associated Risk Factors: A Meta-analysis and Meta-regression | Review Design: Prognostic  Framework: PICOTS  P (Population): infants born EP/VP (<32 weeksâ€™ gestational age [GA]) and/or with extremely low (<1000 g) or very low (<1500 g) birth weight;; preterms as full population; 28 - <32 weeks; VLBW (<1500gr); ELBW (<1000gr)  I / Index / Condition: Gestational age; neonatal complication (BPD, IVH, PVL, NEC, Sepsis); socioeconomic factor  Comparator / Ref.: comparison with full term  Outcome(s): cognition; school age (5-12 years); adolescence (13-18)  Timing: birth and neonatal period; >5years  Setting: This meta-analysis was conducted to determine the role of perinatal and demographic factors in the long-term cognitive outcomes of EP/VP children. It highlighted the significant impact of factors like BPD on cognitive outcomes, suggesting a focus on reducing the incidence of BPD could be beneficial for improving long-term cognitive outcomes in EP/VP childrenâ€‹. | Precheck Critically low |
| Van de Pol, Caroline (2020) | https://dx.doi.org/10.1007/s00431-019-03552-z | Growth patterns and body composition in former extremely low birth weight (ELBW) neonates until adulthood: a systematic review | Review Design: Prognostic  Framework: PICOTS  P (Population): former extremely low birth weight (ELBW) infants; preterms as full population; ELBW (<1000gr)  I / Index / Condition: Gestational age  Comparator / Ref.: Any other prognostic factor/model; control NBW (i.e., 2500-4000 g) infants  Outcome(s): growth/metabolism; 0-3 month; 0-12 month; 12-36 month; preschool age (4-6 years); school age (5-12 years); adolescence (13-18); Other: over 18 years  Timing: birth; after hospital discharge throughout infancy and adulthood  Setting: ELBW infants attain lower growth parameters than NBW control patients after hospital discharge throughout childhood and adolescence.  Although there seems to be a certain degree of catch-up growth for all growth parameters, differences in anthropometric parameters continue to exist with advancing age.  lack of studies investigating body composition in ELBW infants. There is some evidence that they might have an aberrant body composition. Associations between body composition and cardiovascular diseases are suggested | Precheck Critically low |
| van Dokkum, Nienke H. (2021) | https://dx.doi.org/10.1542/peds.2021-050414 | Neonatal Stress, Health, and Development in Preterms: A Systematic Review | Review Design: Prognostic  Framework: PICOTS  P (Population): preterm infants; preterms as full population; "preterm" (<37 weeks)  I / Index / Condition: Other: neonatal stressors related to NICU stay  Comparator / Ref.: comparison with full term  Outcome(s): behaviour/mental health (include ADHD, autism); cognition; neurosensory/neurodevelopmental; motor-development (including CP, DCD); Other: at or after discharge  Timing: birth; childhood  Setting: The setting is the NICU (Neonatal Intensive Care Unit), and the role of the prognostic factors is to evaluate the impact of neonatal stress (like skin-breaking procedures, maternal separation) on various health and developmental outcomes in preterm infants. Neonatal stress has a profound impact on the health and development of preterm infants, and physicians involved in their treatment and follow-up should be aware of this fact. | Precheck Critically low |
| van Hasselt, Tim J. (2023) | https://dx.doi.org/10.1186/s12887-023-04150-7 | Children born preterm admitted to paediatric intensive care for bronchiolitis: a systematic review and meta-analysis | Review Design: Prognostic  Framework: PICOTS  P (Population): children 0-18 years of age admitted to  PICU with RSV and/or bronchiolitis from the year  2000 onwards, taking place in high-income countries as  defined by the World Bank; preterms as full population; "preterm" (<37 weeks)  I / Index / Condition: Gestational age  Comparator / Ref.: comparison with full term  Outcome(s): infections (RSV, ...); Other: 0-18years  Timing: birth; 0-18years  Setting: The setting of the study is the Pediatric Intensive Care Unit (PICU), with the prognostic factor being the status of being born preterm. The study aims to determine the risk of requiring invasive mechanical ventilation and the risk of mortality within PICU for preterm-born children compared to those born at term. Preterm-born children make up a significant and over-represented group within the children admitted to PICU with bronchiolitis. They are at higher risk of requiring invasive respiratory support than children born at term, but no significant increased risk of mortality was observed. | Precheck Critically low |
| van Houdt, Carolien A. (2019) | https://dx.doi.org/10.1111/dmcn.14213 | Executive function deficits in children born preterm or at low birthweight: a meta-analysis | Review Design: Prognostic  Framework: PICOTS  P (Population): children born preterm/low birthweight; preterms as subgroup; "preterm" (<37 weeks); Other: with low birthweight (<2500g)  I / Index / Condition: Gestational age  Comparator / Ref.: comparison with full term  Outcome(s): Other: executive functions: working memory, inhibition, cognitive flexibility; preschool age (4-6 years); school age (5-12 years); adolescence (13-18)  Timing: birth; >4 years of age  Setting: The role of the prognostic factors is to assess the impact of prematurity (specifically, gestational age) on executive functions in children as they grow older. Children born preterm/low birthweight since 1990 perform half a SMD below term-born peers on executive function, which does not seem to improve with more recent advances in medical care or with increasing age. | Precheck Critically low |
| van Lieshout, Pascal (2017) | https://dx.doi.org/10.1016/j.neuro.2016.03.021 | Onset factors in cerebral palsy: A systematic review | Review Design: Prognostic  Framework: PICOTS  P (Population): individuals affected by cerebral palsy (CP); preterms as subgroup; not reported  I / Index / Condition: Gestational age; Other: LBW, chorioamnionitis of the mother, Preeclampsia,  Outcome(s): motor-development (including CP, DCD); not reported  Timing: birth; not reported  Setting: The intended setting and role of the prognostic factors are primarily in the context of understanding the onset and risk factors associated with cerebral palsy. Selected studies indicated that lower gestational age was associated with the onset of CP. | Precheck Critically low |
| van Noort-van der Spek, Inge L. (2012) | https://dx.doi.org/10.1542/peds.2011-1728 | Language functions in preterm-born children: a systematic review and meta-analysis | Review Design: Prognostic  Framework: PICOTS  P (Population): children born preterm (gestational age â‰¤ 37 weeks); preterms as full population; "preterm" (<37 weeks)  I / Index / Condition: Gestational age  Comparator / Ref.: comparison with full term  Outcome(s): language development; 12-36 month; preschool age (4-6 years); school age (5-12 years)  Timing: birth; from 3 to 12 years of age  Setting: While growing up, preterm-born children have increasing difficulties with complex language function. | Precheck Critically low |
| van 't Westende, Charlotte (2022) | https://dx.doi.org/10.1111/dmcn.15133 | Neonatal quantitative electroencephalography and long-term outcomes: a systematic review | Review Design: Prognostic  Framework: PICOTS  P (Population): includes infants with a postconceptional age (PCA) below 46 weeks; preterms as subgroup; not reported  I / Index / Condition: Other: quantitative electroencephalogram (EEG),  conventional and amplitude-integrated EEG  Outcome(s): cognition; neurosensory/neurodevelopmental; motor-development (including CP, DCD); Other: after at least 2 years  Timing: neonatal; >2years  Setting: significant associations between neonatal quantitative EEG measures and long-term outcomes. amplitude, background pattern, and power spectrum-related measures are associated with long-term outcomes in several domains, including the cognitive and motor domains.  Our findings underscore that quantitative EEG analysis has the potential to improve neonatal care since it can provide new prognostic information in infants at risk. | Precheck Critically low |
| van Vliet, Elvira O. G. (2013) | https://dx.doi.org/10.1001/jamapediatrics.2013.1199 | Perinatal infections and neurodevelopmental outcome in very preterm and very low-birth-weight infants: a meta-analysis | Review Design: Prognostic  Framework: PICOTS  P (Population): very preterm and very low-birth-weight (VLBW) infants, specifically those born at â‰¤32 weeks of gestation or with a birth weight of â‰¤1500 g; preterms as full population; <28 weeks; 28 - <32 weeks; VLBW (<1500gr)  I / Index / Condition: neonatal complication (BPD, IVH, PVL, NEC, Sepsis)  Comparator / Ref.: Any other prognostic factor/model; very preterm/VLBW infants without perinatal infections  Outcome(s): neurosensory/neurodevelopmental; motor-development (including CP, DCD); not reported  Timing: perinatal period; not reported  Setting: The results of this meta-analysis highlight the additional deteriorating effect of infections on neurodevelopment impairments in very preterm/VLBW infants and stress the clinical importance of the prevention of perinatal infections. | Precheck Critically low |
| Van't Hooft, Janneke (2015) | https://dx.doi.org/10.1186/s13643-015-0058-7 | Predicting developmental outcomes in premature infants by term equivalent MRI: systematic review and meta-analysis | Review Design: Prognostic  Framework: PICOTS  P (Population): infants born at a gesta- tional age ≤ 32 weeks and/or birth weight ≤1500 g; preterms as full population; 28 - <32 weeks; VLBW (<1500gr)  I / Index / Condition: Other: MRI result at term equivalent age  Outcome(s): behaviour/mental health (include ADHD, autism); cognition; neurosensory/neurodevelopmental; motor-development (including CP, DCD); language development; Other: ≥ 18 months postnatal age  Timing: term equivalent age; ≥ 18 months postnatal age  Setting: The intended setting of the prognostic factors (MRI results) is in clinical practice for evaluating the long-term developmental outcomes of very preterm or low-birth-weight infants. The role of these prognostic factors is to help predict developmental outcomes based on MRI findings at term equivalent age. his study shows that presence of moderate/severe WMA on MRI around term equivalent age can predict CP and motor function in very preterm or low-birth-weight infants with moderate sensitivity and specificity. Its ability to predict other long-term outcomes such as neurocognitive and behavioral impairments is limited. | Precheck Critically low |
| Vieira, Martina Estevam Brom (2016) | https://dx.doi.org/10.1007/s11136-016-1259-9 | Quality of life of individuals born preterm: a systematic review of assessment approaches | Review Design: Prognostic  Framework: PICOTS  P (Population): individuals born preterm; preterms as full population; "preterm" (<37 weeks)  I / Index / Condition: Gestational age  Comparator / Ref.: comparison with full term; psychometric properties of the QoL instruments (table 1)  measures of self-reported QoL and parental measures of QoL,  factors associated with QoL in preterm individuals (table 2)  Outcome(s): Quality of Life; adolescence (13-18); Other: 1month-12years, adult  Timing: birth; infancy to adolescence  Setting: There was a high rate of parental report to assess QoL in studies of children. Adolescent and adult studies most often assessed QoL through self-report.  Parents of children who were born preterm reported worse QoL for their children compared with parents of children born full term. Teenagers and adults who were born preterm self-reported more positive out- comes in their QoL.  The main risk factors associated with worse QoL in children who were born preterm were congenital malformations, mechanical ventilation during the neonatal phase, cognitive impairments, behavioral problems, physical disabilities, low family income, and black race. We recommend that QoL assessments in children born preterm should consider both parent report and self- report | Precheck Critically low |
| Villamor-Martinez, Eduardo (2019) | https://dx.doi.org/10.3389/fphys.2019.00800 | Cerebellar Hemorrhage in Preterm Infants: A Meta-Analysis on Risk Factors and Neurodevelopmental Outcome | Review Design: Prognostic  Framework: PICOTS  P (Population): infants with cerebellar hemorrhage born preterm and LBW; preterms as full population; "preterm" (<37 weeks)  I / Index / Condition: Gestational age; neonatal complication (BPD, IVH, PVL, NEC, Sepsis); Other: LBW,  maternal, obstetric, or perinatal risk factors  Comparator / Ref.: Any other prognostic factor/model; infants without CBH  Outcome(s): behaviour/mental health (include ADHD, autism); cognition; neurosensory/neurodevelopmental; motor-development (including CP, DCD); not reported  Timing: perinatal; not reported  Setting: The review is focused on assessing the long-term neurological impacts of cerebellar hemorrhage in a specific population of preterm infants, making it a vital resource for understanding and improving outcomes in this vulnerable group. | Precheck Critically low |
| Visser, Simone S. M. (2021) | https://dx.doi.org/10.1016/j.smrv.2021.101447 | The relationship between preterm birth and sleep in children at school age: A systematic review | Review Design: Prognostic  Framework: PICOTS  P (Population): school-aged children born preterm; preterms as full population; "preterm" (<37 weeks)  I / Index / Condition: Gestational age  Comparator / Ref.: comparison with full term  Outcome(s): Other: sleep; school age (5-12 years)  Timing: birth; school age  Setting: The overall conclusion is that prematurity is associated with earlier bedtimes and a lower sleep quality, in particular more nocturnal awakenings and more non- rapid eye movement stage 2 sleep. | Precheck Critically low |
| Walker, Emma J. (2019) | https://dx.doi.org/10.1016/j.vaccine.2019.02.031 | Completeness and timeliness of diphtheria-tetanus-pertussis, measles-mumps-rubella, and polio vaccines in young children with chronic health conditions: A systematic review | Review Design: Prognostic  Framework: PICOTS  P (Population): Children born preterm, with LBW or chronic health condition; preterms as subgroup; "preterm" (<37 weeks)  I / Index / Condition: Gestational age  Outcome(s): Other: timeliness of vaccinations; 0-12 month; 12-36 month; preschool age (4-6 years)  Timing: birth; childhood  Setting: Preterm infants were the most commonly-reported special population and polio and DTP were the most frequently-reported vaccines. We found that estimates of coverage and timeliness of routine childhood vaccinations in these special populations were highly variable, ranging from 40% to 100% and 12% to 81%, respectively, across the vaccines and population group | Precheck Critically low |
| Walton, Kathryn (2022) | https://dx.doi.org/10.1093/advances/nmac017 | Eating Behaviors, Caregiver Feeding Interactions, and Dietary Patterns of Children Born Preterm: A Systematic Review and Meta-Analysis | Review Design: Prognostic  Framework: PICOTS  P (Population): Infants and children born preterm (<37 weeks of gestation) and aged 6 mo postnatal or corrected age to 7 y at the time of data col lection; preterms as full population; "preterm" (<37 weeks)  I / Index / Condition: Gestational age  Comparator / Ref.: comparison with full term  Outcome(s): Other: oromotor eating skills, eating behaviors, dietary patterns; 0-12 month; 12-36 month; preschool age (4-6 years); school age (5-12 years)  Timing: birth; 6 months up to 7 years of age  Setting: The study found that children born before the full term (preterm children) have more problems with eating skills and behavior when they are babies and young kids, compared to children born at full term. The study also suggests that preterm children often have more difficult and stressful feeding times with their parents or caregivers, which is linked to higher stress levels in mothers. | Precheck Critically low |
| Wang, Chengzhong (2017) | https://dx.doi.org/10.1097/MD.0000000000006696 | Prenatal, perinatal, and postnatal factors associated with autism: A meta-analysis | Review Design: Prognostic  Framework: PICOTS  P (Population): children diagnosed with autism; preterms as subgroup; "preterm" (<37 weeks)  I / Index / Condition: Gestational age  Comparator / Ref.: comparison with full term  Outcome(s): behaviour/mental health (include ADHD, autism); not reported  Timing: birth; not reported  Setting: gestational age <=36 weeks is associated with an increased risk for autism (RR=1.31, 95% CI: 1.16, 1.48; P<.001) | Precheck Critically low |
| Wang, D. (2011) | https://dx.doi.org/10.3310/hta15050 | Palivizumab for immunoprophylaxis of respiratory syncytial virus (RSV) bronchiolitis in high-risk infants and young children: a systematic review and additional economic modelling of subgroup analyses | Review Design: Prognostic  Framework: PICOTS  P (Population): children with RSV infection; preterms as subgroup; 28 - <32 weeks; 32 - <37 weeks  I / Index / Condition: Other: immunoprophylaxis with palivizumab  Comparator / Ref.: comparison with full term  Outcome(s): Other: cost-effectiveness; 0-12 month; 12-36 month; preschool age (4-6 years)  Timing: birth; up to 5 years  Setting: Prophylaxis with palivizumab does not represent good value for money based on the current UK incremental cost-effectiveness ratio (ICER) threshold of Â£30,000/QALY when used unselectively in children without CLD/CHD or children with CLD or CHD. | Precheck Critically low |
| Wang, Yang-Feng (2018) | https://dx.doi.org/10.1080/10245332.2017.1396056 | Gestational age and childhood leukemia: A meta-analysis of epidemiologic studies | Review Design: Prognostic  Framework: PICOTS  P (Population): preterm or postterm infants; preterms as subgroup; "preterm" (<37 weeks)  I / Index / Condition: Gestational age  Outcome(s): Other: ALL, AML, childhood leukemia; not reported  Timing: birth; childhood  Setting: Our results suggest that preterm infants have an elevated risk of developing AML | Precheck Critically low |
| Wong, Hilary S. (2013) | https://dx.doi.org/10.1007/s10995-012-1183-8 | Nature or nurture: a systematic review of the effect of socio-economic status on the developmental and cognitive outcomes of children born preterm | Review Design: Prognostic  Framework: PICOTS  P (Population): children born in or after 1990 and before 37 completed weeks of gestation; preterms as full population; "preterm" (<37 weeks); VLBW (<1500gr); ELBW (<1000gr)  I / Index / Condition: socioeconomic factor  Outcome(s): cognition; 12-36 month; preschool age (4-6 years); school age (5-12 years)  Timing: birth; childhood  Setting: Maternal education below high school level was associated with severe cognitive deficiency. SES appears to confound the association between preterm birth and cognitive deficit and should be adjusted for in studies reporting cognitive outcome. | Precheck Critically low |
| Wu, Xiaoyan (2020) | https://dx.doi.org/10.1080/14767058.2018.1527310 | Association of molar incisor hypomineralization with premature birth or low birth weight: systematic review and meta-analysis | Review Design: Prognostic  Framework: PICOTS  P (Population): infants with premature birth and low birth weight; preterms as subgroup; <28 weeks; 28 - <32 weeks; 32 - <37 weeks; VLBW (<1500gr); ELBW (<1000gr)  I / Index / Condition: Gestational age; Other: low birth weight  Comparator / Ref.: comparison with full term  Outcome(s): teeth; preschool age (4-6 years); school age (5-12 years); adolescence (13-18)  Timing: birth; childhood and adolescence  Setting: Premature birth and low birth weight increase the prevalence of MIH. Over time, MIH will aggravate, and its adverse consequences will become severe. An appropriate dental care strategy is urgently needed. | Precheck Critically low |
| Xiao, Dongqiong (2018) | https://dx.doi.org/10.1371/journal.pone.0208302 | Maternal chorioamnionitis and neurodevelopmental outcomes in preterm and very preterm neonates: A meta-analysis | Review Design: Prognostic  Framework: PICOTS  P (Population): infants with and without maternal chorioamnionitis exposure; preterms as full population; 28 - <32 weeks  I / Index / Condition: Other: maternal chorioamnionitis  Outcome(s): neurosensory/neurodevelopmental; motor-development (including CP, DCD); 12-36 month  Timing: in utero; 18-24 months  Setting: This study suggests that maternal chorioamnionitis may affect mental development in pre-term and very preterm neonates, and that maternal clinical chorioamnionitis may affect motor development in offspring | Precheck Critically low |
| Xing, Lu (2019) | https://dx.doi.org/10.1097/MD.0000000000018229 | Is chorioamnionitis associated with neurodevelopmental outcomes in preterm infants? A systematic review and meta-analysis following PRISMA | Review Design: Prognostic  Framework: PICOTS  P (Population): preterm infants with maternal chorioamnionitis; preterms as full population; "preterm" (<37 weeks)  I / Index / Condition: Other: maternal chorioamnionitis  Outcome(s): cognition; neurosensory/neurodevelopmental; motor-development (including CP, DCD); language development; 0-12 month; 12-36 month; preschool age (4-6 years)  Timing: in utero; short-term (<12 months), mid-term (12-36 months), long-term (>36 months)  Setting: In preterm infants, chorioamnionitis might be a risk factor for performance and verbal intelligence quotient impairment and severe cognitive deficits, and clinical CA might be a risk factor for overall psychomotor and language deficits | Precheck Critically low |
| Xu, Shan (2022) | https://dx.doi.org/10.3389/fped.2022.975340 | Relationship between preterm, low birth weight, and development defects of enamel in the primary dentition: A meta-analysis | Review Design: Prognostic  Framework: PICOTS  P (Population): preterm and LBW infants <6 year of age; preterms as subgroup; "preterm" (<37 weeks); VLBW (<1500gr); Other: LBW  I / Index / Condition: Gestational age; Other: LBW, VLBW  Comparator / Ref.: comparison with full term  Outcome(s): teeth; 0-12 month; 12-36 month; preschool age (4-6 years)  Timing: birth; less than or equal to 6 years old  Setting: This meta-analysis demonstrated that both PT and LBW especially VLBW are associated with a higher risk of DDE in the primary dentition. PT and LBW are both related to the occurrence of EHP. However, the relationship between PT, LBW, and enamel opacity has not been verified. | Precheck Critically low |
| Zhao, Mengwen (2016) | https://dx.doi.org/10.1038/srep38853 | SGA as a Risk Factor for Cerebral Palsy in Moderate to Late Preterm Infants: a System Review and Meta-analysis | Review Design: Prognostic  Framework: PICOTS  P (Population): moderate or late premature infants; preterms as full population; 32 - <37 weeks  I / Index / Condition: Other: SGA  Comparator / Ref.: Any other prognostic factor/model; non-SGA  Outcome(s): motor-development (including CP, DCD); not reported  Timing: birth; not reported  Setting: In moderate to late premature infants, SGA is a convenient and reliable predictor for CP. More studies are needed to explore the underlying mechanisms. | Precheck Critically low |
| Zhu, Tingting (2018) | https://dx.doi.org/10.1016/j.jaad.2017.12.015 | Association of very preterm birth with decreased risk of eczema: A systematic review and meta-analysis | Review Design: Prognostic  Framework: PICOTS  P (Population): very preterm birth; preterms as full population; 28 - <32 weeks; 32 - <37 weeks  I / Index / Condition: Gestational age  Comparator / Ref.: comparison with full term  Outcome(s): Other: eczema; 12-36 month; preschool age (4-6 years); school age (5-12 years)  Timing: birth; childhood  Setting: The available evidence suggests an association between very preterm birth and a decreased risk of eczema. | Precheck Critically low |
| Adama, Esther Abena (2016) | 10.1016/j.jnn.2015.07.006 | Parents' experiences of caring for preterm infants after discharge from Neonatal Intensive Care Unit: A meta-synthesis of the literature | Review Design: Qualitative  Framework: CoCoPop  P (Population): Parents (mothers and fathers) of preterm infants  Context (CoCoPop): After discharge from the Neonatal Intensive Care Unit (NICU) | Precheck Critically low |
| Lopez, Greta L. (2012) | nan | Transition of premature infants from hospital to home life | Review Design: Qualitative  Framework: PICO  P (Population): premature infants and their parents; preterms as full population; "preterm" (<37 weeks)  I / Index / Condition: Other: transition home  Comparator / Ref.: not reported  Outcome(s): Other: components of transition home programs; not reported | Precheck Critically low |
| Øberg, G. K. (2023) | 10.3389/fpsyg.2023.1172578 | A systematic synthesis of qualitative studies on parents' experiences of participating in early intervention programs with their infant born preterm | Review Design: Qualitative  Framework: PICO  P (Population): mothers or fathers of infants born preterm (gestational age <37 weeks at birth); preterms as full population; "preterm" (<37 weeks)  I / Index / Condition: 02a=parent-child centered; early intervention programm  Comparator / Ref.: not reported  Outcome(s): Parent-Child-Interaction; not reported | Precheck Critically low |
| Orton, J (2024) | https://dx.doi.org/10.1002/14651858.CD005495.pub5 | Early developmental intervention programmes provided post hospital discharge to prevent motor and cognitive impairment in preterm infants | Review Design: Interventional  Framework: PICO  P (Population): Preterm infants, "preterm" (<37 weeks), preterm as full population  I / Index / Condition: Early developmental intervention programmes (initiated within the first 12 months of life, possibly beginning in hospital but must include a post-discharge component; can include therapies such as physiotherapy, occupational therapy, parent-infant relationship enhancement, infant stimulation, etc.).  Comparator / Ref.: Standard follow-up care typically provided by the institution or community service.   Outcome(s): Motor and cognitive development outcomes, assessed at three different age ranges: Infancy (0 to <3 years), preschool age (3 to <5 years), school age (5 to <18 years) | AMSTAR High |
